# Orthogonal protection of saccharide polyols through solvent-free one-pot sequences based on regioselective silylations

**DOI:** 10.3762/bjoc.12.271

**Published:** 2016-12-14

**Authors:** Serena Traboni, Emiliano Bedini, Alfonso Iadonisi

**Affiliations:** 1 Department of Chemical Sciences, University of Naples Federico II, Via Cinthia 4, 80126, Naples, Italy

**Keywords:** carbohydrates, one-pot synthesis, regioselective protection, silyl protecting group, solvent-free reaction

## Abstract

*tert*-Butyldimethylsilyl (TBDMS) and *tert*-butyldiphenylsilyl (TBDPS) are alcohol protecting groups widely employed in organic synthesis in view of their compatibility with a wide range of conditions. Their regioselective installation on polyols generally requires lengthy reactions and the use of high boiling solvents. In the first part of this paper we demonstrate that regioselective silylation of sugar polyols can be conducted in short times with the requisite silyl chloride and a very limited excess of pyridine (2–3 equivalents). Under these conditions, that can be regarded as solvent-free conditions in view of the insolubility of the polyol substrates, the reactions are faster than in most examples reported in the literature, and can even be further accelerated with a catalytic amount of tetrabutylammonium bromide (TBAB). The strategy proved also useful for either the selective TBDMS protection of secondary alcohols or the fast per-*O*-trimethylsilylation of saccharide polyols. In the second part of the paper the scope of the silylation approach was significantly extended with the development of unprecedented “one-pot” and “solvent-free” sequences allowing the regioselective silylation/alkylation (or the reverse sequence) of saccharide polyols in short times. The developed methodologies represent a very useful and experimentally simple tool for the straightforward access to saccharide building-blocks useful in organic synthesis.

## Introduction

The application of an orthogonal set of protecting groups represents a typical issue in organic synthesis in the elaboration of highly functionalized molecules such as carbohydrates, and lengthy multistep procedures are often needed to this aim [1–2]. Silyl groups are widely applied in organic chemistry in orthogonal protection strategies owing to their stability to a broad range of conditions and feasible removal under conditions compatible with many other used alcohol protecting groups [1–3]. For this reason, silyl protecting groups are often serving as temporary protecting groups with polyol and saccharide substrates. The most robust and adopted silyl protecting groups are featuring the presence of hindered substituents at silicon such as in *tert*-butyldimethylsilyl (TBDMS) and *tert*-butyldiphenylsilyl (TBDPS) groups. Their bulkiness allows in many cases regioselective silyl protection of primary alcohols. Commonly, *O*-silylation is performed by exposing the alcohol to a suitably substituted silyl chloride in the presence of a base, a catalyst (often coinciding with the base) and an aprotic solvent [2–9]. The use of more expensive silyl triflates is also reported, especially when poorly reactive alcohols have to be protected [2,10–13].

A regioselective silylation of polar saccharide polyols is typically performed with the appropriate silyl chloride in the presence of a high boiling solvent such as DMF or pyridine, often in the presence of a nucleophilic catalyst (more frequently imidazole and DMAP) [14–21]. The protection generally takes several hours and the work-up is burdened by necessary removal of the high boiling solvent. A good regioselective control was also reported in an alternative silylation approach based on a dehydrogenative mechanism in which expensive trialkyl silanes were used as silylating agents [22]. Very recently, Vogel and co-workers reported an original strategy based on unusual silylating agents such as silyl methallylsulfinates; this approach proved very high-yielding under neutral conditions, but the preliminary synthesis of the requisite reagent relied on a non-trivial two-step procedure starting from the corresponding silyl chloride [23].

The regioselective silylation of secondary saccharide alcohols can also be achieved either taking advantage of the inherent difference of reactivity among the hydroxy functions [24–32] or exploiting the activation effect of boron complexes [33].

Over the last years, we have addressed our interest towards the development of solvent-free protocols aimed at regioselective protection of highly functionalized saccharide substrates. This effort led to a simple protocol for the selective benzylation of primary saccharide alcohols [34], the first catalytic tin-mediated procedure for regioselective benzylation/allylation of hydroxy groups incorporated into vicinal diols [35], and three alternative acetalation protocols [36]. Besides the avoided use of solvents, these approaches appear advantageous owing to their experimental ease, the reactions being performed under air by simply mixing the requisite reagents.

In this paper, we wish to report the extension of the scope of the solvent-free strategies to the silylation reaction, and the feasible incorporation of this step into unprecedented one-pot, fully solvent-free sequences yielding orthogonally protected saccharide building-blocks under simple experimental conditions and in short times.

## Results and Discussion

In preliminary experiments, methyl mannopyranoside (**1**) was selected as the model substrate and exposed to TBDMSCl in the presence of a slight or moderate excess of several bases (Table 1).

**Table 1:** Regioselective silylation of **1** under solvent free conditions^a^.

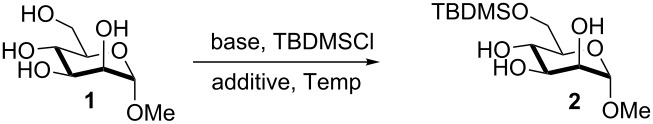				

Entry	Base (equiv)	Additive (equiv)	Temperature, time	Isolated yield

1	DIPEA (5)	–	50 °C, 5 h	<15
2	DIPEA (5)	TBAB (0.3)	50 °C, 5.5 h	49
3	DIPEA (5)	TBAB (0.3), Bu_2_SnO (0.1)	50 °C, 5.5 h	52
4	DIPEA (5)	TBAI (0.3), Bu_2_SnO (0.1)	50 °C, 5.5 h	48
5	TEA (5)	TBAB (0.3)	50 °C, 5.5 h	53
6	pyridine (5)	TBAB (0.3)	50 °C, 1 h	89
7	pyridine (2.5)	TBAB (0.3)	rt, 1 h	80
8	pyridine (2.2)	TBAB (0.1)	rt, 1.5 h	84
9	pyridine (2.2)	–	rt, 1.5 h	70

^a^General conditions: substrate, base, additive, TBDMSCl (1.2 equiv for entries 1–6, 1.1 equiv for entries 7–9).

In all cases 6-*O*-silylated derivative **2** was obtained as the main product, but yields and rates were strongly dependent on the adopted base: pyridine (Table 1, entries 6–9) gave much better results than tertiary amines (Table 1, entries 1–5) that, on the other hand, had previously performed better than pyridine in the tin-catalyzed solvent-free regioselective benzylation or allylation of sugars [35]. The silylation rate was not appreciably influenced by tin catalysis (compare entries 2 and 3 in Table 1), although a previous report described the stoichiometric use of stannylene acetals in the regioselective silylation of saccharide primary alcohols [37]. At this stage, it should be noted that pyridine is frequently employed as the solvent for silylations, but the conditions herein described can be referred to as “solvent-free” because of the very limited amount of pyridine used, that is by far not sufficient for dissolution of the polar polyol substrates. Interestingly, reactions with pyridine were found to give slightly improved yields (within comparable times) on using a catalytic amount of TBAB (compare in Table 1 entries 6–8 with entry 9), which may be accounted for by a possible role of the bromide ion in the activation of the silylating agent as also suggested by literature [38]. Taking into account several parameters such as the used amount of pyridine and TBAB, the reaction yield and its length, we elected conditions of Table 1, entry 8 as the optimized conditions. Notably, the conversion of **1** to **2** under these conditions took a much shorter time than previously reported by using the same silylating agent [14–15,18]. With optimized conditions in hand, the TBDMS regioselective installation was tested on a range of saccharide building-blocks, and good yields were achieved in short times with several polyols (Table 2, entries 1–3, 5 and 8).

**Table 2:** Regioselective silylation of saccharide primary alcohols under solvent-free conditions^a^.

Entry	Substrate	Pyridine/silylating agent/TBAB (equivalents)^b^	Time (h)	Product, isolated yield

1	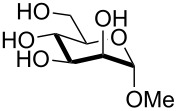 **1**	pyridine/TBDMSCl/TBAB (2.2:1.1:0.1)	1.5	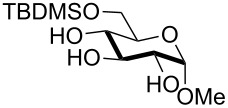 **2**, 84%
2	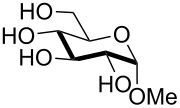 **3**	pyridine/TBDMSCl/TBAB (2.2:1.1:0.1)	1.5	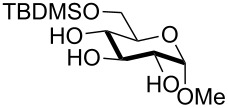 **10**, 83%
3	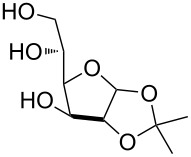 **4**	pyridine/TBDMSCl/TBAB (2.2:1.1:0.1)	1.5	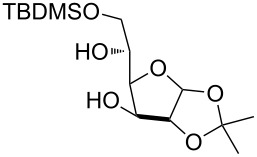 **11**, 86%
4	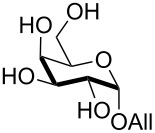 **5**	pyridine/TBDMSCl/TBAB (2.2:1.1:0.1)	1.5	low conversion
5	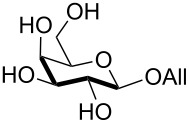 **6**	pyridine/TBDMSCl/TBAB (3.0:2.0:0.1)	2.5	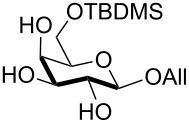 **12**, 82%
6	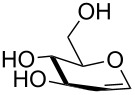 **7**	pyridine/TBDMSCl/TBAB (2.2:1.1:0.1)	1.5	complex mixture
7	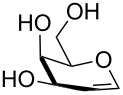 **8**	pyridine/TBDMSCl/TBAB (2.2:1.1:0.1)	1.5	complex mixture
8^c^	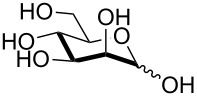 **9**	pyridine/TBDMSCl/TBAB (2.2:1.1:0.1)	2	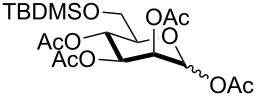 **13**, 73% (α/β 1:2)
9	**1**	pyridine/TBDPSCl/TBAB (2.2:1.1:0.2)	3	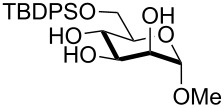 **14**, 68%
10	**1**	pyridine/TBDPSCl/TBAB (3.0:1.1:0.2)	3	**14**, 92%
11	**3**	pyridine/TBDPSCl/TBAB (3.0:1.1:0.2)	3	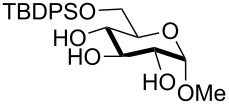 **15**, 84%
12	**4**	pyridine/TBDPSCl/TBAB (3.0:1.1:0.2)	2	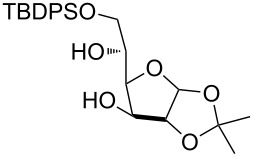 **16**, 98%
13	**7**	pyridine/TBDPSCl/TBAB (3.0:1.1:0.2)	5	very sluggish

^a^General conditions: polyol substrate, pyridine, silylating agent (TBDMSCl or TBDPSCl), and TBAB at rt. See pertinent entries for stoichiometric ratios. ^b^With respect to the polyol substrate. ^c^The crude silylation mixture was acetylated in situ (direct addition of pyridine and acetic anhydride), prior to purification of the product.

Interestingly, *galacto*-configured substrates exhibited a peculiar reactivity with the reaction outcome depending on their anomeric configuration. In accordance with yields obtained by Lee and Taylor with similar substrates under standard conditions [14], only allyl β-galactoside (**6**) was silylated in a good yield (Table 2, entry 5), whereas the corresponding α-anomer was almost quantitatively recovered (Table 2, entry 4). As will be shown below, partial protection of allyl α-galactoside in itinere can render this substrate much more reactive towards these silylation conditions. High-yielding silylation of allyl β-galactoside (**6**) required the employment of a higher stoichiometric excess of both pyridine and TBDMSCl (Table 2, entry 5), exhibiting an extent of conversion apparently ruled by an equilibrium-like control.

Among the screened polyols in Table 2, glycals **7** and **8** were the only substrates to exhibit a lower regioselectivity, the silylation rate being comparable at the primary (O-6) and the allylic (O-3) position, to provide a mixture of products (Table 2, entries 6 and 7). A similar competitive reactivity was very recently described also with the above mentioned method based on silyl methallylsulfinates [23]. On the other hand, the method herein proposed was found compatible with a reducing sugar such as D-mannose, which was converted in a good yield into the corresponding 6-*O*-silylated product **13**, isolated after in situ peracetylation (Table 2, entry 8).

The scope of the TBAB-catalyzed silyl protection under solvent-free conditions was next examined for the regioselective attachment of TBDPS (Table 2, entries 9–13), a commonly used silyl protecting group bulkier than TBDMS and more resistant to acidic conditions. Optimization on mannoside **1** indicated that in this case a moderate increase of the pyridine excess (3.0 instead of 2.2 equivalents) is beneficial for the achievement of a higher yield while maintaining the use of a minimal excess of the silylating agent (Table 2, entries 9 and 10). As with TBDMS protection of **6** (Table 2, entry 5), in this case a sort of steady state was observed when adopting less than three equivalents of pyridine, with coexistence of the reagent and the product.

The TBDPS selective protection of polyols **3** and **4** proceeded in high yields (Table 2, entries 11 and 12), and expectedly the reactions took slightly longer times than for TBDMS protection. The only disappointing result observed in attempted TPDPS protections was the poor yield recorded with glucal **7** (Table 2, entry 13), a surprising outcome which is somehow consistent with the very slow rate observed for the same reaction under standard conditions [39–40]. As already observed for the synthesis of **2**, the herein described conditions for regioselective TBDMS and TBDPS protections entail shorter reaction times than most of the reported protocols in the literature on monosaccharide polyols [14–18,20,41–53]; comparable silylation rates were indeed be found in a few examples, often involving relatively less polar thioaryl glycosides as the substrates and DMF as the solvent [19,54–59]. Having established the scope of TBAB-catalyzed mono silylations with a minimal excess of pyridine, some effort was devoted to ascertain the feasible exploitation of a similar strategy to either regioselective di-*O*-silylations or the protection of secondary alcohols in absence of primary ones (Table 3).

**Table 3:** Solvent-free regioselective silylations committing secondary alcohols^a^.

Entry	Substrate	Time (h)	Product, isolated yield

1	**1**	4	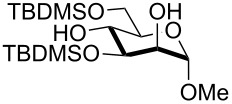 **17**, 72%
2	**3**	4.5	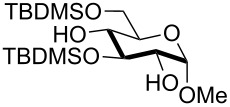 **18**, 60%
3	**8**	5	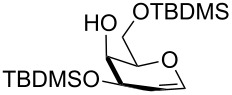 **19**, 56%
4	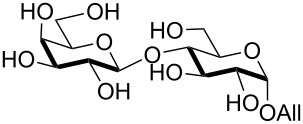 **20**	1	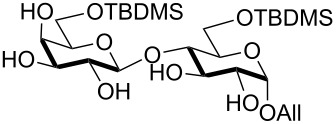 **21**, 56%
5	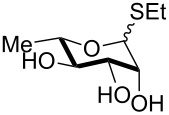 **22** (α:β ca. 2.5)	6	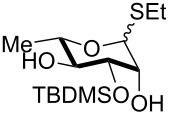 **23** (α:β ca. 4), 75%
6	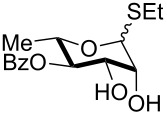 **24** (α:β ca. 2.5)	6	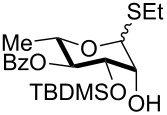 **25** (α:β ca. 4), 50% (95%)^b^

^a^General conditions (entries 1–3): pyridine (5 equiv), TBDMSCl (2.5 equiv), TBAB (0.3 equiv), 50 °C. For entry 4: pyridine (6 equiv), TBDMSCl (3.5 equiv), TBAB (0.3 equiv), rt. For entries 5 and 6: pyridine (3 equiv), TBDMSCl (1.5 equiv), TBAB (0.15 equiv), 50 °C. ^b^In parenthesis is indicated the conversion yield.

As a matter of fact, upon doubling the stoichiometric amount of TBAB, pyridine and TBDMSCl, the regioselective synthesis of di-*O*-TBDMS derivatives was achieved at 50 °C in satisfying yields from glycosides **1** and **3** (Table 3, entries 1 and 2), and glycal **8** (entry 3).

Double silylation at primary positions of the disaccharide lactoside **20** [60] also proved feasible at room temperature within short times (Table 3, entry 4). Model substrates devoid of a primary alcohol were also examined; thioethyl rhamnoside **22** (as an anomeric mixture) was 3-*O*-silylated in a good yield, and the anomeric composition of the products revealed the higher reactivity of the α-anomer (Table 3, entry 5). The same regioselectivity was also found starting from the corresponding 4-*O*-benzoylated precursor **24** [61] (Table 3, entry 6) and a satisfying yield and an excellent conversion were observed in spite of the increased hindrance and deactivation of the O-3 hydroxy group due to the adjacent electron-withdrawing benzoyl group. As already described above in Table 1 for mono-silylations, tin catalysis did not affect the double silylation processes as evidenced by the reaction of Table 3, entry 1 that in the presence of 0.1 equiv of Bu_2_SnO gave **17** in the same yield within the same time. Not unexpectedly, secondary alcohols exhibited a recalcitrant reactivity towards TBDPSCl under these solvent-free conditions, and the di-*O*-silylation process resulted in limited synthetic usefulness (data not shown).

**Scheme 1:**
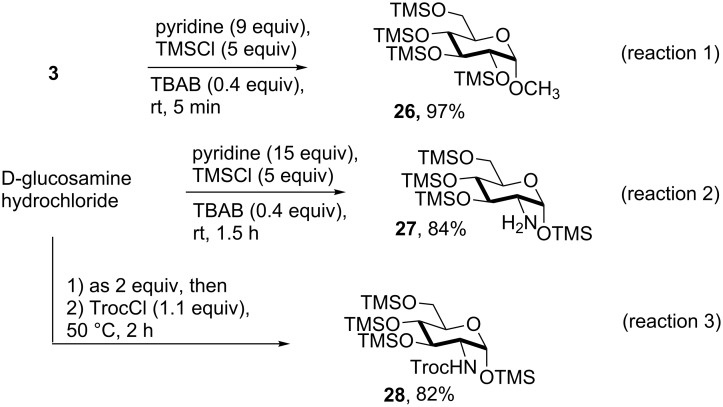
Multiple *O*-trimethylsilylations of saccharide compounds.

The scope of the solvent-free conditions was further examined in the synthesis of per-*O*-trimethylsilylated derivatives (Scheme 1), widely used precursors in one-pot strategies for orthogonal protection of carbohydrates [62–67].

Application of the TBAB-catalyzed protocol on glucoside **3** gave product **26** in high yield within a few minutes (Scheme 1, reaction 1). The method proved also applicable to glucosamine hydrochloride, although in this case a higher excess of pyridine was needed for the conversion to occur (Scheme 1, reaction 2). Consistent with previous literature reports [68–72], silylation left unaltered the amino functionality which could be protected in situ with a Troc group without isolation of **27** [69], as shown in the one-pot, two-step sequence in Scheme 1, reaction 3.

Owing to the importance of the one-pot functional diversification of carbohydrates in modern organic synthesis [62–67,73–76], the scope of the solvent-free silylation approaches herein introduced was next extended to the development of fully solvent-free one-pot sequences leading to the sequential alkylation/silylation of saccharide polyols with high regiocontrol. For this purpose, we tried to combine the previously reported tin-catalyzed procedure for benzylation/allylation of saccharide secondary alcohols [35] with the present protocol for silylation of primary alcohols. Some experiments were carried out on methyl mannoside (**1**) in order to establish which order of steps (alkylation/silylation or the reverse sequence) might be higher yielding. Initial experiments indicated that the silylation step was not apparently effective when performed after the tin-catalyzed 3-*O*-benzylation of mannoside (**1**, Table 4, entry 1). A competitive reaction of residual benzyl bromide from the first step with pyridine may account for this result. Indeed, on repeating the experiment suitably increasing the amount of pyridine in the second step (from 2.2 to 5 equivalents), the desired product **29** was obtained in a satisfying 61% yield within a few hours (Table 4, entry 2). It should be outlined that this result is especially relevant taking into account that the solvent-free 3-*O*-benzylation alone (the first step of the sequence) occurs in a comparable yield under tin catalysis [35]. The reverse protection sequence (6-*O*-silylation/3-*O*-benzylation) was found to be less effective in terms of yields, apparently because of the partial loss of the TBDMS group in the second step where relatively forced thermal conditions are needed (Table 4, entry 3). This latter one-pot sequence was instead much more rewarding when the more robust TBDPS group was installed first (Table 4, entries 5 and 6). Interestingly, this latter group was satisfyingly stable under the especially forced conditions (a reaction temperature of 90 °C) required to carry out the tin-mediated 2-*O*-benzylation of a *gluco*-substrate (Table 4, entry 6). In addition, the TBDPS installation also proceeded in satisfying yields after a preliminary allylation (Table 4, entry 4) or benzylation step (Table 4, entry 8).

**Table 4:** One-pot, regioselective protection of sugar polyols with silyl and alkyl groups^a^.

Entry	Substrate	First step conditions (equiv)	Second step conditions (equiv)	Product, isolated yield

1	**1**	DIPEA (2.5), Bu_2_SnO (0.1), TBAB (0.3), BnBr (4), 70 °C, 3.5 h	pyridine (2.2), TBDMSCl (2.0), rt, 3 h	low silylation yields
2	**1**	DIPEA (2.5), Bu_2_SnO (0.1), TBAB (0.3), BnBr (4), 70 °C, 3.5 h	pyridine (5.0), TBDMSCl (2.0), rt, 3 h	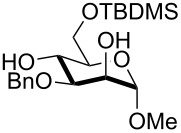 **29**, 61%
3	**1**	pyridine (2.2), TBDMSCl (1.1), TBAB (0.3), rt, 1.5 h	DIPEA (3.5), Bu_2_SnO (0.1), BnBr (5.5), 70 °C, 4.5 h	**29**, 39%
4	**1**	DIPEA (4), Bu_2_SnO (0.1), TBAB (0.3), AllBr (8), 90 °C, 3.5 h	pyridine (5.0), TBDPSCl (1.5), rt, 3.5 h	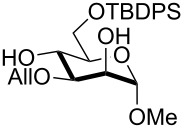 **30**, 59%
5	**1**	pyridine (3.0), TBDPSCl (1.1), TBAB (0.3), rt, 3 h	DIPEA (2.5), Bu_2_SnO (0.1), BnBr (6), 80 °C, 4 h	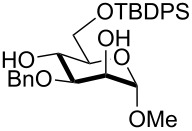 **31**, 62%
6	**3**	pyridine (3.0), TBDPSCl (1.1), TBAB (0.3), rt, 3 h	DIPEA (2.5), Bu_2_SnO (0.2), BnBr (7), 90 °C, 4 h	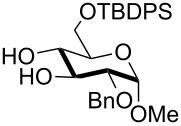 **32**, 42%
7	**8**	DIPEA (2.5), Bu_2_SnO (0.1), BnBr (2), TBAB (0.3) 70 °C, 2.5 h	pyridine (3.5), TBDMSCl (2.0), TBAB (0.3), rt, 1 h	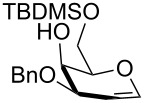 **33**, 69%
8	**8**	DIPEA (2.5), Bu_2_SnO (0.1), BnBr (2), TBAB (0.3), 70 °C, 2.5 h	pyridine (3.5), TBDPSCl (1.5), rt, 2.5 h	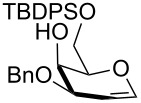 **34**, 75%
9	**5**	DIPEA (2.5), Bu_2_SnO (0.1), BnBr (4), TBAB (0.3), 70 °C, 2 h	pyridine (6), TBDMSCl (2), rt, 1.5 h	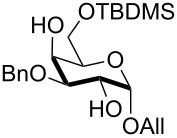 **35**, 57%

^a^General conditions: upon completion of the first step (see times in pertinent entries), the temperature was modified according to conditions of the second step, and the requisite reagents added.

In the case of glycal **8**, the application of a benzylation/silylation sequence resulted in very good yields with both silylating agents (Table 4, entries 7 and 8), although the 6-*O*-silylation alone had been low yielding in the initial set of experiments (Table 2, entries 6, 7 and 13). Comparison of these data seems to indicate that the silylation step is much more effective on less polar, partially protected polyols. As a further evidence of this trend, the 3-*O*-benzylation/6-*O*-silylation sequence was succesfully applied to access α-galactoside **35** (Table 4, entry 9), although, as shown earlier in Table 2, entry 4, direct silylation of the same precursor was quite unfruitful.

## Conclusion

In the first part of this paper we have introduced a very simple approach to carry out the selective TBDMS or TBDPS protection of carbohydrate polyols taking advantage of reactions performed in the presence of a very limited amount of pyridine (3 equivalents or less for the hydroxy group to be protected). The method can indeed be regarded as a solvent-free approach because of the limited stoichiometric amount of the base that is not sufficient to dissolve the highly polar saccharide substrates. Under these conditions, a catalytic role played by TBAB was also evidenced. Besides the limited amount of the used base, the proposed method is endowed with further practical advantages such as the experimental ease (all reaction herein reported were conducted in air by simply mixing the reagents), and high reaction rates, often comparing favourably with literature examples, in spite of the poor solubility of the starting substrates in the reaction medium. The scope of the silylation approach was also extended to secondary alcohols and to the per-*O*-trimethylsilylation of saccharides.

In the second part of the paper, we have explored the feasible combination of the silylation methodology here introduced with a previously developed tin-catalyzed methodology for regioselective alkyl protection of carbohydrates. The merging of both methods led to unprecedented one-pot and fully solvent-free synthetic sequences, providing a straightforward and experimentally simple access to saccharide building-blocks orthogonally protected with a silyl at the primary position and a benzyl (or an allyl) group at well defined and predicatable secondary positions.

## Supporting Information

File 1 Experimental and analytical data.
