# Supplementary material for: Orthogonal protection of saccharide polyols through solvent-free one-pot sequences based on regioselective silylations
Source: Beilstein J Org Chem. 2016 Dec 14;12:2748–56. doi: 10.3762/bjoc.12.271 (PMC5238545; doi:10.3762/bjoc.12.271)
Supplement: File 1 — Experimental and analytical data. [file Beilstein_J_Org_Chem-12-2748-s001.pdf]

Supporting Information

for

Orthogonal protection of saccharide polyols through solvent-free one-pot sequences based on regioselective silylations

Serena Traboni, Emiliano Bedini and Alfonso Iadonisi*

Address: Department of Chemical Sciences, University of Naples Federico II, Via Cinthia 4, 80126, Naples (Italy)

Email: Alfonso Iadonisi - iadonisi@unina.it

*Corresponding author

Experimental and analytical data

Experimental section

Typical procedure for the regioselective mono-*O*-silylation: To a mixture of the polyol substrate (0.5–1 mmol), TBAB and the silylating agent, pyridine was added under air (see Tables 1 and 2 for stoichiometric proportions). The mixture was kept under stirring at room temperature until consumption of the starting material as revealed by TLC analysis (1.5–3 hours). The mixture was concentrated under vacuum and then submitted to silica-gel flash cromatography (eluents: ethyl acetate or ethyl acetate/hexane mixtures) to afford the mono-*O*-silylated products in the yields indicated in Tables 1 and 2.

Regioselective double silylation of monosaccharide polyols or silylation of secondary carbinols: To a mixture of the polyol substrate (0.5–1 mmol), TBAB and the silylating agent, pyridine was added under air (see Table 3 for stoichiometric proportions). The mixture was kept under stirring at 50 °C. Upon completion of the reaction (1–6 hours), the mixture was concentrated under vacuum and then submitted to silica-gel flash chromatography (eluents: hexane/ethyl acetate mixtures) to afford the di-*O*-silylated products in the yields indicated in Table 3. The regiochemistry of the double silylation was determined by acetylation of the isolated products (2:1v/v pyridine/acetic anhydride, overnight, rt) and subsequent NMR analysis.

One-pot synthesis of orthogonally protected building-blocks (silylation–alkylation sequence): Upon completion of the mono-O-silylation step (see above for the procedure), to the mixture were

sequentially added under air DIPEA, benzyl bromide, and Bu_2SnO, and the vessel placed on an oil bath at the suitable temperature (see Table 4 for stoichiometric proportions and the temperature of the second step). The mixture was kept under stirring until TLC analysis indicated optimal conversion. The reaction vessel was cooled to rt and the mixture was diluted with DCM. The organic phase was washed with aqueous NaOH and the aqueous phase re-extracted with DCM. The combined organic phases were dried with anhydrous sodium sulfate and concentrated under vacuum. Flash chromatography (eluents: hexane/ethyl acetate mixtures) provided pure products in the yields indicated in Table 4.

One-pot synthesis of orthogonally protected building-blocks (alkylation-silylation sequence): To a mixture of the substrate (0.5–1 mmol), Bu_2SnO and TBAB, were sequentially added under air DIPEA and benzyl (or allyl) bromide (see Table 4 for stoichiometric proportions). The mixture was kept under stirring at 70 °C (or 90 °C for regioselective allylation) until TLC analysis indicated optimal conversion. The flask was cooled to rt and then pyridine and the silylating agent were sequentially added. On completion of the reaction, the mixture was diluted with DCM. The organic phase was washed with aqueous NaOH and the aqueous phase was re-extracted with DCM. Combined organic phases were dried with anhydrous sodium sulfate and concentrated under vacuum. Flash chromatography (eluents: hexane/ethyl acetate mixtures) provided pure products in the yields indicated in Table 4.

Spectral data

Methyl 6-*O*-*tert*-butyldimethylsilyl-α-D-mannopyranoside (2).[1-3]

^{1}H NMR (400 MHz, $CDCl_3$) δ 4.67 (s, 1H, H-1), 4.25 (bs, exchangeable, 1H), 4.02 (bs, exchangeable, 1H), 3.90-3.80 (overlapped signals, 3H), 3.76 (bdd, *J* = 2.8 and 9.6 Hz, 1H, H-3), 3.69 (t, *J* = 9.6 Hz, 1H, H-4), 3.53 (m, 1H, H-5), 3.34 (s, 3H, $-OCH_3$), 0.89 (s, 9H, *t*-butyl protons), 0.09 (s, 6H, $-Si(CH_3)_2$) ppm. ^{13}C NMR (100 MHz, $CDCl_3$) δ 100.7 (C-1), 71.7, 71.4 (C-3, C-5), 70.3 (C-2), 69.5 (C-4), 64.3 (C-6), 54.7 ($-OCH_3$), 25.8 ($-SiC(\mathit{C}H_3)_3$), 18.2 ($-Si\mathit{C}(CH_3)_3$), - 5.46 (-Si $(CH_3)_2$) ppm . Anal. Calcd. for $C_{13}H_{28}O_6Si$: C, 50.62; H, 9.15. Found: C, 50.75; H, 9.10.MALDI-MS $[M + Na]^+$ calcd. for ($C_{13}H_{28}O_6Si$)331.16, found 331.30.

Methyl 6-*O*-*tert*-butyldimethylsilyl-α-D-glucopyranoside (10). [4-6]

^{1}H NMR (400 MHz, $CDCl_3$) δ 5.32 (bs, exchangeable, 1H), 4.69 (d, *J* = 3.6 Hz, 1H, H-1),4.61 (bs, exchangeable, 1H), 4.55 (bs, exchangeable, 1H), 3.87 (bd, *J* = 10.2 Hz, 1H, H-6a), 3.75 (dd, *J* = 5.6 and 10.2 Hz, 1H, H-6b), 3.71 (t, *J* = 9.6 Hz, 1H, H-3), 3.55-3.45 (overlapped signals, 2H), 3.37 (s,

3H, -OCH_3), 3.49 (t, *J* = 9.6 Hz, 1H, H-4), 0.88 (s, 9H, *t*-butyl protons), 0.06 (s, 6H, -$Si(CH_3)_2$) ppm. ^{13}C NMR (100 MHz, $CDCl_3$): δ = 99.3 (C-1), 74.1 (C-3), 71.9, 71.7 (C-5, C-2), 70.8 (C-4), 63.3 (C-6), 54.9 (-OCH_3), 25.9 (-SiC(CH_3)$_3$), 18.3 (-Si*C*(CH_3)$_3$), -5.3 (-Si $(CH_3)_2$) ppm. Anal. Calcd. for $C_{13}H_{28}O_6Si$: C, 50.62; H, 9.15. Found: C, 50.55; H, 9.15.MALDI-MS [M + Na]$^+$ calcd. for ($C_{13}H_{28}O_6Si$) 331.16, found 331.05.

1,2-*O*-Isopropylidene-6-*O*-*tert*-butyldimethylsilyl-α-D-glucofuranose (11).[7]

^{1}H NMR (400 MHz, $CDCl_3$) δ 5.90 (d, *J* = 3.2 Hz, 1H, H-1), 4.49 (d, *J* = 3.2 Hz, 1H, H-2), 4.30 (d, *J* = 2.0 Hz, 1H, H-3), 4.04 (dd, *J* = 2.0 and 6.8 Hz, 1H, H-4), 3.97 (m, 1H, H-5), 3.82 (dd, *J* = 3.6 and 10.0 Hz, 1H, H-6a), 3.71 (dd, *J* = 4.8 and 10.0 Hz, 1H, H-6b), 3.19 (bs, exchangeable), 1.49 and 1.32 (2 x s, 6H, isopropylidene methyls), 1.09 (s, 9H, t-butyl protons), 0.06 (s, 6 H, -$Si(CH_3)_2$) ppm. ^{13}C NMR (100 MHz, $CDCl_3$): δ 111.6 (isopropylidene quaternary C), 104.8 (C-1), 85.0 (C-2), 79.5 (C-4), 75.3 (C-3), 69.9 (C-5), 64.0 (C-6), 26.6, 26.1 (isopropylidene CH_3), 25.7 (-SiC(*C*H_3)$_3$), 18.2 (-Si*C*(CH_3)$_3$), -5.5 (-Si $(CH_3)_2$) ppm. Anal. Calcd. for $C_{15}H_{30}O_6Si$: C, 53.86; H, 9.04. Found: C, 53.80; H, 9.15. MALDI-MS [M + Na]$^+$ calcd. for ($C_{15}H_{30}O_6Si$)357.40, found 357.30.

Allyl 6-*O*-*tert*-butyldimethylsilyl-β-D-galactopyranoside (12).[8]

^{1}H NMR (400 MHz, $CDCl_3$) δ 6.00-5.90 (m, 1H, -C*H*=CH_2), 5.28 (bd, *J* = 17.2 Hz, 1H, -C*H*=C$H_a$$H_b$), 5.17 (bd, *J* = 10.4 Hz, 1H, -C*H*=CH_aH_b), 4.34 (dd, *J* = 5.2 and 12.4 Hz, 1H, H-6a), 4.24 (d, *J* = 7.6 Hz, 1H, H-1), 4.11 (dd, *J* = 6.4and 12.4 Hz , 1H, H-6b), 3.94 (bs, 1H, H-4), 3.80-3.60 (m, 2 H, -CH_2CH=CH_2), 3.70 (bt, *J* = 8.8 Hz, 1H, H-2), 3.56 (m, 1H, H-3), 3.43 (m, 1H, H-5), 0.87 (s, 9H, t-butyl protons), 0.06(s, 6 H, -$Si(CH_3)_2$) ppm. ^{13}C NMR (100 MHz, $CDCl_3$) δ 134.0 (CH_2=*C*H-), 117.9 (*C*H_2=CH-), 101.9 (C-1), 75.0 (C-5), 73.7 (C-3), 71.2 (C-2), 70.0 (CH_2=CH*C*H_2-), 68.7 (C-4), 62.2 (C-6), 25.8 (-SiC(*C*H_3)$_3$), 18.2 (-Si*C*(CH_3)$_3$), -5.4 (-Si $(CH_3)_2$) ppm. Anal. Calcd. for $C_{15}H_{30}O_6Si$: C, 53.86; H, 9.04. Found: C, 53.70; H, 9.10. MALDI-MS [M + Na]$^+$ calcd. for ($C_{15}H_{30}O_6Si$) 357.40, found 357.50.

6-*O*-*tert*-Butyldimethylsilyl-1,2,3,4-tetra-*O*-acetyl-α/β-D-mannopyranose (13). (α/β 1:2.1): ^{1}H NMR (400 MHz, $CDCl_3$) δ 6.03 (d, *J* = 1.6 Hz , 1 H, H-1α), 5.80 (bs, 1H, H-1β), 5.40 (d, *J* = 2.4 Hz, 1H, H-2β),5.35 (t, *J* = 9.6 Hz , 1H, H-4α), 5.29 (dd, *J* = 1.6 and 2.4 Hz, 1H, H-2α), 5.28 (t, *J* = 10.0 Hz, 1H, H-4β), 5.18 (dd, *J* = 2.4 and 10.0 Hz, 1H, H-3β), 3.72 (m, 1H, H-5α), 3.80-3-65 (m, 4H), 3.57 (m, 1H, H-5β); 2.13, 2.10, 2.04, 1.99 (x3), 1.96 (x2) (5 x s, 24 H, acetyl methyls), 0.85 (s, 18 H, *t*-butyl protons), 0.01 and – 0.01 (2 x s, 12 H, 2 x -$Si(CH_3)_2$) ppm. ^{13}C NMR (100 MHz, $CDCl_3$): δ 170.4, 169.8, 169.3, 168.3 (*C*OCH_3), 90.5 (C-1 α), 90.2 (C-1 β), 75.7 (C-5 β), 73.2 (C-5

α), 70.9 (C-3 β), 69.0 (C-2 α), 68.4 (C-2 β), 68.3 (C-4 β), 65.8, 65.7 (C-3 α, C-4 α), 62.1 (C-6 β), 61.8 (C-6 α), 25.6 (-SiC(*C*H_3)$_3$), 20.7-20.4 (CO*C*H_3), 18.1 (-Si*C*(CH_3)$_3$), -5.5 (-Si (CH_3)$_2$) ppm. Anal. Calcd. for $C_{20}H_{34}O_{10}Si$: C, 51.93; H, 7.41. Found: C, 51.80; H, 7.45. MALDI-MS $[M + Na]^+$ calc. for ($C_{20}H_{34}O_{10}Si$) 485.18, found 485.45.

Methyl 6-*O*-*tert*-butyldiphenylsilyl-α-D-mannopyranoside (14).[9-10]

^{1}H NMR (400 MHz, $CDCl_3$) δ 7.80-7.30 (aromatic H), 4.65 (s, 1H, H-1), 3.93 (dd, *J* = 2.8 and 8.8 Hz, 1H, H-3), 3.90-3.80 (overlapped signals, 2H), 3.80-3.70 (overlapped signals, 2H), 3.61 (m, 1H), 3.29 (s, 3H, -OCH_3), 1.05 (s, 6H, -Si(CH_3)$_2$) ppm. ^{13}C NMR (100 MHz, $CDCl_3$): δ 135.7, 132.9, 129.9, 127.6 (aromatic signals), 100.6 (C-1), 71.7, 71.1 (C-3 and C-5), 70.3 (C-2), 69.8 (C-4), 65.0 (C-6), 54.7 (-OCH_3), 26.7 (-SiC(*C*H_3)$_3$), 19.1 (-Si*C*(CH_3)$_3$) ppm. Anal. Calcd. for $C_{23}H_{32}O_6Si$: C, 63.86; H, 7.46. Found: C, 63.95; H, 7.45. MALDI-MS $[M + Na]^+$ calcd. for ($C_{23}H_{32}O_6Si$) 455.19, found 455.05.

Methyl 6-*O*-*tert*-butyldiphenylsilyl-α-D-glucopyranoside (15).[6, 11, 12]

^{1}H NMR (400 MHz, $CDCl_3$): δ 7.80-7.30 (aromatic H), 4.80 (bs, exchangeable, 1H), 4.70 (d, *J* = 3.6 Hz, 1H, H-1), 4.00-3.85 (overlapped signals, 2H), 3.82 (dd, *J* = 5.4 and 10.8 Hz, 1H, H-6b), 3.76 (t, *J* = 9.2 Hz, 1H, H-3), 3.64 (m, 1H, H-5), 3.55-3.43 (overlapped signals, 2H), 3.34 (s, 3H), 1.04 (s, 9H, -Si(CH_3)$_2$) ppm. ^{13}C NMR (100 MHz, $CDCl_3$): δ 135.7, 133.2, 129.6, 127.7 (aromatic signals), 99.1 (C-1), 74.3 (C-3), 72.0 (C-2), 71.5 (C-5), 71.1 (C-4), 64.0 (C-6), 54.8 (-OCH_3), 26.8 (-SiC(*C*H_3)$_3$), 19.1 (-Si*C*(CH_3)$_3$) ppm. Anal. Calcd. for $C_{23}H_{32}O_6Si$: C, 63.86; H, 7.46. Found: C, 63.75; H, 7.50. MALDI-MS $[M + Na]^+$ calcd. for ($C_{23}H_{32}O_6Si$) 455.19, found 455.40.

1,2-*O*-Isopropylidene-6-*O*-*tert*-butyldiphenylsilyl-α-D-glucofuranose (16).[13]

^{1}H NMR (400 MHz, $CDCl_3$): δ 7.80-7.30 (aromatic H), 5.96 (d, *J* = 3.2 Hz, 1H, H-1), 4.54 (d, *J* = 3.2 Hz, 1H, H-2),4.40 (d, *J* = 1.6Hz, 1H, H-3),4.21 (dd, *J* = 1.6 and 6.4 Hz, 1H, H-4), 4.12 (m, 1H, H-5), 3.91 (dd, *J* = 4.0 and 10.4 Hz, 1H, H-6a), 3.85 (dd, *J* = 5.2 and 10.0 Hz, 1H, H-6b), 1.44 and 1.28 (2 x s, 6H), 0.89 (s, 9H), 0.06 (s, 6H, -Si(CH_3)$_2$) ppm. ^{13}C NMR (100 MHz, $CDCl_3$): δ 135.5, 132.2, 129.8, 127.8 (aromatic signals), 111.4 (isopropylidene quaternary C), 104.8 (C-1), 85.0 (C-2), 79.2 (C-4), 75.4 (C-3), 70.2 (C-5), 64.8 (C-6), 26.7 (-SiC(*C*H_3)$_3$), 26.7, 26.1 (isopropylidene CH_3), 19.1 (-Si*C*(CH_3)$_3$) ppm. Anal. Calcd. for $C_{25}H_{34}O_6Si$: C, 65.47; H, 7.47. Found: C, 65.67; H, 7.35. MALDI-MS $[M + Na]^+$ calcd. for ($C_{25}H_{34}O_6Si$) 481.20, found 481.30.

Methyl 3,6-di-*O-tert*-butyldimethylsilyl-α-D-mannopyranoside (17).[14, 15]

^{1}H NMR (400 MHz, $CDCl_3$):δ 4.71 (s, 1H, H-1), 3.86 (d, *J* = 5.6 Hz, 2H, H_2-6), 3.83 (dd, *J* = 3.6 and 9.2 Hz, 1H, H-3), 3.74 (bd, *J* = 2.0 Hz, 1 H, H-2), 3.70 (t, *J* = 9.2 Hz, 1H, H-4), 3.55 (m, 1H, H-5), 3.36 (s, 3H, 1-OCH_3), 2.70 (bs, exchangeable, 1H), 2.57 (bs,exchangeable, 1H), 0.91 and 0.90 (2 x s, 18 H, *t*-butyl protons); 0.15, 0.13, 0.09 (x2) (3 x s, 18H, 2 x -$Si(CH_3)_2$) ppm. ^{13}C NMR (100 MHz, $CDCl_3$): δ 100.0 (C-1), 72.9 (C-3), 71.0, 70.4 (x2) (C-2, C-4, C-5), 64.9 (C-6), 54.8 (-OCH_3), 25.8, 25.7 (2 x -SiC($C$$H_3$)$_3$), 18.1 (2 x -Si$C$($CH_3$)$_3$) -4.5, -4.9, -5.5 (x2) (-Si (CH_3)$_2$) ppm. Anal. Calcd. for $C_{19}H_{42}O_6Si_2$: C, 53.99; H, 10.01. Found: C, 53.80; H, 9.95. MALDI-MS $[M + Na]^+$ calcd. for ($C_{19}H_{42}O_6Si_2$) 445.24, found 445.45.

Methyl 2,6-di-*O-tert*-butyldimethylsilyl-α-D-glucopyranoside (18).[16]

^{1}H NMR (400 MHz, $CDCl_3$): δ 4.60 (d, *J* = 3.2 Hz, 1H, H-1), 3.85-3.82 (overlapped signals, 2H), 3.80 (t, *J* = 9.2 Hz, 1H, H-3), 3.60 (m, 1H, H-5), 3.54 (dd, *J* = 3.2 and 9.2 Hz, 1H, H-2), 3.51 (t, *J* = 9.2 Hz, 1H, H-4), 3.38 (s, 3H), 0.90 (s, 18H), 0.14, 0.11, 0.10 (x2) (3 x s, 12 H) ppm. ^{13}C NMR (100 MHz, $CDCl_3$): δ 99.8 (C-1), 73.9 (C-3), 73.4 (C-2), 71.8 (C-4), 70.5 (C-5), 64.0 (C-6), 55.2 (-OCH_3), 25.9, 25.8 (2 x -SiC($C$$H_3$)$_3$), 18.3, 18.1 (2 x -Si$C$($CH_3$)$_3$), -4.6, -5.4 (-Si ($CH_3$)$_2$) ppm. Anal. Calcd. for $C_{19}H_{42}O_6Si_2$: C, 53.99; H, 10.01. Found: C, 53.95; H, 10.05. MALDI-MS $[M + Na]^+$ calc. for ($C_{19}H_{42}O_6Si_2$) 445.24, found 445.10.

3,6-Di-*O-tert*-butyldimethylsilyl-D-galactal (19).[17]

^{1}H NMR (400 MHz, $CDCl_3$): δ 6.45 (d, *J* = 6.0 Hz, 1H, H-1), 4.63 (d, *J* = 6.0 Hz, 1H, H-2), 4.57 (d, *J* = 2.4 Hz, 1H), 4.20-3.95 (overlapped signals, 4H), 2.82 (s, exchangeable 1H), 1.03 and 1.02 (2 x s, 18H, *t*-butyl protons), 0.24 and 0.21 (2 x s, 12H, 2 x -$Si(CH_3)_2$) ppm. ^{13}C NMR (100 MHz, $CDCl_3$): δ 144.2 (C-1), 102.2 (C-2), 76.8 (C-5), 65.0, 64.8 (C-3, C-4), 61.9 (C-6), 25.9, 25.8 (2 x -SiC($C$$H_3$)$_3$), 18.3, 18.0 (2 x -Si$C$($CH_3$)$_3$), -4.6, -4.9, -5.3, -5.4 (-Si (CH_3)$_2$) ppm. Anal. Calcd. for $C_{18}H_{38}O_4Si_2$: C, 57.70; H, 10.22. Found: C, 57.75; H, 10.35. MALDI-MS $[M + Na]^+$ calc. for ($C_{18}H_{38}O_4Si_2$) 397.22, found 397.45.

Allyl 6´-*O-tert*-butyldimethylsilyl-β-D-galacatopyranosyl-(1→4)-6-*O-tert*-butyldimethylsilyl-β-D-glucopyranoside (21). $[\alpha]_D^{25}$+41 (*c* 1.0, $CHCl_3$);^{1}H NMR (400 MHz, $CDCl_3$): δ 6.00-5.90 (m, 1H, -C*H*=CH_2), 5.41 (bd, *J* = 17.6 Hz, 1H, -CH=CH_aH_b), 5.41 (bd, *J* = 10.4 Hz, 1H,-CH=CH_aH_b), 4.98 (d, *J* = 3.2 Hz, 1H, H-1), 4.47 (d, *J* = 7.6 Hz, 1H, H-1′), 4.26 (dd, *J* = 6.0 and 12.8 Hz, 1H, –C*Ha*HbCH=CH_2), 4.13 (dd, *J* = 6.0 and 12.8 Hz, 1H, –CHa*Hb*CH=CH_2), 4.09 (bs, 1H, H-4′), 4.00-3.70 (overlapped signals), 1.00 (s, 18H, *t*-butyl protons), 0.18 (s, 9H, -$Si(CH_3)_2$) ppm. ^{13}C NMR

(100 MHz, $CDCl_3$): δ 133.7 (CH_2=*C*H), 117.8 (*C*H_2=CH), 103.1 (C-1′), 98.9 (C-1), 79.0 (C-4), 75.1 (C-5′), 73.8 (C-3′), 72.3, 72.2, 71.3, 70.7 (C-2, C-3, C-5, C-2′), 68.4, 68.3 (C-4′ and CH_2=CH*C*H_2-), 62.4, 61.7 (C-6, C-6′), 25.8 (-SiC(*C*H_3)$_3$), 18.3 (-Si*C*(CH_3)$_3$), -5.28 (-Si (CH_3)$_2$) ppm. Anal. Calcd. for $C_{27}H_{54}O_{11}Si_2$: C, 53.09; H, 8.91. Found: C, 53.15; H, 8.80. MALDI-MS $[M + Na]^+$ calcd. for ($C_{27}H_{54}O_{11}Si_2$) 633.31, found 633.15.

Ethyl 3-*O*-*tert*-butyldimethylsilyl-α/β-L-1-thio-rhamnopyranoside (23).

(α/β ca 4:1).^{1}H NMR (400 MHz, $CDCl_3$): signals of prevalent α-anomer at δ 5.30 (s, 1H, H-1), 4.02 (m, 1H, H-5), 3.89 (bs, 1H, H-2), 3.79 (dd, *J* = 3.2 and 8.8 Hz, 1H, H-3), 3.53 (t, *J* = 8.8 Hz, 1H, H-4), 2.75-2.55 (m, 2H, -C*H_2*CH_3), 1.31 (t, *J* = 5.6 Hz, 3 H, -CH_2C*H_3*), 0.92-0.89 (overlapped signals), 0.15 and 0.13 (2 x s, 6H, -Si(CH_3)$_2$) ppm. ^{13}C NMR (100 MHz, $CDCl_3$): δ 83.0 (C-1), 74.0, 73.6, 73.2 (C-2, C-3, C-4), 68.0 (C-5), 25.7 (-SiC(*C*H_3)$_3$), 24.9 (-S*C*$H_2$$CH_3$), 18.1 (-Si*C*($CH_3$)$_3$), 17.5 (C-6), 14.8 (-S$CH_2$*C*$H_3$), -4.6, -4.7 (-Si($CH_3$)$_2$) ppm. Anal. Calcd. for $C_{14}H_{28}O_4SSi$: C, 52.13; H, 9.38. Found: C, 52.30; H, 9.30. MALDI-MS $[M + Na]^+$ calcd. for ($C_{14}H_{28}O_4SSi$) 345.15, found 345.35.

Ethyl 4-*O*-benzoyl-3-*O*-*tert*-butyldimethylsilyl-α/β-L-1-thio-rhamnopyranoside (25).

(α:β 4:1).^{1}H NMR (400 MHz, $CDCl_3$): signals of prevalent α-anomer at δ = 8.00-7.30 (aromatic Hs, H), 5.38 (s, 1H, H-1), 5.28 (t, *J* = 8.8 Hz, 1H, H-4), 4.25 (m, 1H, H-5), 4.08 (dd, *J* = 2.4 and 9.2 Hz, 1H, H-3), 3.97 (bs, 1H, H-2), 2.75-2.50 (m, 2H, -C*H_2*CH_3), 1.31 (t, *J* = 7.2 Hz, 3H, -CH_2C*H_3*), 1.22 (d, *J* = 6.4 Hz, 3H, H_3-6), 0.77 (s, 9H, t-butyl protons), 0.04, -0.1 (2 x s, 6H, -Si(CH_3)$_2$) ppm.^{13}C NMR (100 MHz, $CDCl_3$):δ = 135.5, 133.1, 129.7, 128.4 (aromatic signals), 82.9 (C-1), 74.5, 73.1, 71.2 (C-2, C-3, C-4), 66.6 (C-5), 25.4 (-SiC(*C*H_3)$_3$), 24.9 (-S*C*$H_2$$CH_3$), 18.1 (-Si*C*($CH_3$)$_3$), 17.3 (C-6), 14.8 (-S$CH_2$*C*$H_3$), -4.6, -4.7 (-Si($CH_3$)$_2$) ppm. Anal. Calcd. for $C_{21}H_{34}O_5SSi$: C, 59.12; H, 8.05. Found: C, 59.00; H, 8.15. MALDI-MS $[M + Na]^+$ cald. for ($C_{21}H_{34}O_5SSi$) 449.18, found 449.05.

Methyl 2,3,4,6-tetra-*O*-trimethylsilyl-α-D-glucopyranoside (26).[18,19]

^{1}H NMR (400 MHz, $CDCl_3$): δ 4.68 (d, *J* = 3.6 Hz, 1H, H-1), 3.85-3.80 (overlapped signals, 2 H), 3.76 (dd, *J* = 4.8 and 11.6 Hz, 1H, H-6a), 3.60-3.50 (overlapped signals, 3H), 3.41 (s, 3H, -OCH_3); 0.24, 0.23 (x2), 0.20 (3 x s, 12H, 4 x -Si(CH_3)$_3$). ^{13}C NMR (100 MHz, $CDCl_3$): δ = 99.6 (C-1), 75.2 (C-3), 73.9 (C-2), 72.1 (C-4), 71.9 (C-5), 62.1 (C-6), 54.4 (-OCH_3), 1.2, 0.8, 0.4 -0.3 (4 x -Si(CH_3)$_3$). Anal. Calcd. for $C_{19}H_{46}O_6Si_4$: C, 47.26; H, 9.60. Found: C, 47.05; H, 9.70.

2-Amino-2-deoxy-1,3,4,6-tetra-*O*-trimethylsilyl-α-D-glucopyranose (27).[20-24]

^{1}H NMR (400 MHz, $CDCl_3$): δ 5.05 (d, *J* = 3.2 Hz, 1H, H-1), 3.70-3.55 (overlapped signals, 3H), 3.47 (t, *J* = 8.8 Hz, 1H, H-3), 3.43 (t, *J* = 8.8 Hz, 1H, H-4), 2.47 (dd, *J* = 3.2 and 8.8 Hz, 1H, H-2), 0.14, 0.11, 0.09, 0.03 (4 x s, 12H, 4 x -$Si(CH_3)_3$) ppm. ^{13}C NMR (100 MHz, $CDCl_3$): δ 94.5 (C-1), 77.5 (C-3), 72.7 (C-5), 71.9 (C-4), 61.9 (C-6), 57.3 (C-2), 1.2, 0.7, -0.2, -0.4 (4 x -$Si(CH_3)_3$) ppm. Anal. Calcd. for $C_{18}H_{45}NO_5Si_4$: C, 46.22; H, 9.69. Found: C, 46.10; H, 9.80.

2-Deoxy-2-trichloroethoxycarbonylamino-1,3,4,6-tetra-*O*-trimethylsilyl-α-D-glucopyranose (28).[21]

^{1}H NMR (400 MHz, $CDCl_3$): δ 5.08 (d, *J* = 2.4 Hz, 1H, H-1), 4.87 (d, *J* = 9.2 Hz, 1H, -NHTroc), 4.81 and 4.59 (2 x d, AB, *J* = 12.0 Hz, 2 H, -C$H_2$$CCl_3$), 3.75-3.50 (overlapped signals, 6H), 0.16, 0.14, 0.13, 0.08 (4 x s, 12H, 4 x -$Si(CH_3)_3$) ppm. ^{13}C NMR (100 MHz, $CDCl_3$): δ 154.1 (carbamate CO), 92.5 (C-1), 74.7 (-O*C*H_2CCl_3), 73.8, 72.4, 71.9 (C-3, C-4, C-5), 61.6 (C-6), 56.8 (C-2), 0.9, 0.7, -0.2, -0.3 (4 x -$Si(CH_3)_3$) ppm. Anal. Calcd. for $C_{21}H_{46}Cl_3NO_7Si_4$: C, 39.21; H, 7.21. Found: C, 39.20; H, 7.10.

Methyl 3-O-benzyl-6-*O*-*tert*-butyldimethylsilyl-α-D-mannopyranoside (29).[25-27]

^{1}H NMR (400 MHz, $CDCl_3$): δ 7.40-7.20 (aromatic H), 4.71 (s, 1H, H-1), 4.67 (s, 2H, -CH_2Ph), 3.94 (bs, 1H, 2-H), 3.90-3.84 (overlapped signals, 3H), 3.66 (dd, *J* = 2.8 and 8.8 Hz, H-3), 3.58 (m, 1H, H-5), 3.34 (s, 3H, -OCH_3), 0.90 (s, 9H, *t*-butyl protons), 0.08 (s, 6H, -$Si(CH_3)_2$). ^{13}C NMR (100 MHz, $CDCl_3$): δ 137.9, 128.5, 127.9, 127.8 (aromatic signals), 100.3 (C-1), 79.4 (C-3), 72.0 (-CH_2Ph), 71.0 (C-5), 68.6 (C-4), 67.7 (C-2), 64.4 (C-6), 54.7 (-OCH_3), 25.8 (-SiC(*C*H_3)$_3$), 18.2 (-Si*C*$(CH_3)_3$), -5.5 (-$Si(CH_3)_2$) ppm. Anal. Calcd. for $C_{20}H_{34}O_6Si$: C, 60.27; H, 8.60. Found: C, 60.45; H, 8.50. MALDI-MS $[M + Na]^+$ calcd. for ($C_{20}H_{34}O_6Si$) 421.20, found 421.55.

Methyl 3-*O*-allyl-6-*O*-*tert*-butyldiphenylsilyl-α-D-mannopyranoside (30).[28]

^{1}H NMR (400 MHz, $CDCl_3$): δ 7.85-7.30 (aromatic H), 6.00-5.90 (m, 1H, -C*H*=CH_2), 5.33 (bd, *J* = 17.2 Hz, 1H, -CH=CH_aH_b), 5.22 (bd, *J* = 10.4 Hz, 1H, -CH=CH_aH_b), 4.75 (s, 1H, H-1), 4.20-4.10 (m, 2H, -CH_2CH=CH_2), 4.00 (bs, 1H, H-2), 3.99-3.85 (overlapped signals, 3H), 3.67 (m, 1H, H-5), 3.62 (dd, *J* = 2.8 and 8.8 Hz, 1H, H-3), 3.34 (s, 3H, -OCH_3), 1.07 (s, 9H, *t*-butyl protons) ppm. ^{13}C NMR (100 MHz, $CDCl_3$): δ 135.5, 134.4, 129.6, 127.6 (aromatic), 132.0 (CH_2=*C*H-), 117.6 (*C*H_2=CH-), 100.3 (C-1), 79.0 (C-3), 71.2 (C-5), 70.7 (-*C*H_2CH=CH_2), 68.3 (C-4), 67.6 (C-2), 64.9 (C-6), 54.6 (-OCH_3), 26.7 (-SiC(*C*H_3)$_3$), 19.1 (-Si*C*$(CH_3)_3$) ppm. Anal. Calcd. for $C_{26}H_{36}O_6Si$: C,

66.07; H, 7.68. Found: C, 66.25; H, 7.55. MALDI-MS $[M + Na]^+$ calcd. for ($C_{26}H_{36}O_6Si$) 495.22, found 495.30.

Methyl 3-*O*-benzyl-6-*O*-*tert*-butyldiphenylsilyl-α-D-mannopyranoside (31).[29]

^{1}H NMR (400 MHz, $CDCl_3$): δ 7.90-7.25(aromatic H), 4.88 (s, 1H, H-1), 4.84 (s, 2H, -CH_2Ph), 4.13 (bs, 1H, H-2), 4.10-4.03 (overlapped signals, 3H), 3.90-3.75 (overlapped signals, 2H), 3.48 (s, 3H, -OCH_3), 2.93 (bs, exchangeable, 1H), 2.61 (bs, exchangeable, 1H), 1.22 (s, 9 H, *t*-butyl protons) ppm. ^{13}C NMR (100 MHz, $CDCl_3$):δ = 137.9, 135.6, 133.0, 128.7, 128.5, 127.9, 127.7 (aromatic signals), 100.2 (C-1), 79.5 (C-3), 72.0 (-CH_2Ph), 71.1 (C-5), 68.5 (C-4), 67.7 (C-2), 64.9 (C-6), 54.7 (-OCH_3), 26.7 (-SiC(*C*H_3)$_3$), 19.1 (-Si*C*(CH_3)$_3$) ppm. Anal. Calcd. for $C_{30}H_{38}O_6Si$: C, 68.93; H, 7.33. Found: C, 68.75; H, 7.40. MALDI-MS $[M + Na]^+$ calcd. for ($C_{30}H_{38}O_6Si$) 545.23, found 545.40.

Methyl 2-*O*-benzyl-6-*O*-*tert*-butyldiphenylsilyl-α-D-glucopyranoside (32).[27]

^{1}H NMR (400 MHz, $CDCl_3$): δ 7.85-7.25 (aromatic H), 4.85 and 4.82 (2 x d, AB, *J* = 12.0 Hz, 2H, -CH_2Ph), 4.76 (d, *J* = 3.2 Hz, 1H, H-1), 4.07 (t, *J* = 9.2 Hz, 1H, H-3), 4.04 (dd, *J* = 3.2 and 10.4 Hz, 1H, H-6a), 3.97 (dd, *J* = 5.2and 10.4 Hz, 1H, H-6b), 3.78 (m, 1H, H-5), 3.66 (t, *J* = 9.2 Hz, 1H, H-4), 3.48 (dd, *J* = 3.2 and 9.2 Hz, 1H, H-2), 3.48 (s, 3H, OCH_3), 3.20 (bs, exchangeable, 1H), 1.20 (s, 9H, *t*-butyl protons) ppm. ^{13}C NMR (100 MHz, $CDCl_3$): δ 137.9, 135.5, 133.1, 129.6, 128.4, 128.0, 127.9, 127.6 (aromatic signals), 97.4 (C-1), 79.2 (C-2), 73.0 (-CH_2Ph), 72.9 (C-3), 71.6 (C-4), 70.6 (C-5), 64.3 (C-6), 54.9(-OCH_3), 26.7 (-SiC(*C*H_3)$_3$), 19.1 (-Si*C*(CH_3)$_3$) ppm. Anal. Calcd. for $C_{30}H_{38}O_6Si$: C, 68.93; H, 7.33. Found: C, 68.80; H, 7.35. MALDI-MS $[M + Na]^+$ calcd. for ($C_{30}H_{38}O_6Si$) 545.23, found 545.55.

3-*O*-Benzyl-6-*O*-*tert*-butyldimethylsilyl-D-galactal (33).[30]

^{1}H NMR (400 MHz, $CDCl_3$): δ 7.40-7.30 (aromatic H), 6.40 (d, *J* = 6.0 Hz, 1H, H-1),4.70 (d, *J* = 6.0 Hz, 1H, H-2), 4.69-4.60 (AB, *J* = 12.0 Hz, 2H, -CH_2Ph), 4.21 (bs, 1H), 4.14 (bs, 1H), 3.97 (m, 1H), 3.90-3.80 (m, 2H), 2.63 (bs, 1H), 0.92 (s,9H, *t*-butyl protons), 0.11 (s, 6H, -Si(CH_3)$_2$) ppm. ^{13}C NMR (100 MHz, $CDCl_3$): δ 144.9 (C-1), 137.7, 128.5, 127.9, 127.7 (aromatic signals), 99.5 (C-2), 76.6 (C-5), 70.9 (-CH_2Ph), 70.4 (C-3), 62.4, 62.1 (C-4, C-6), 26.0 (-SiC(*C*H_3)$_3$), 19.2 (-Si*C*(CH_3)$_3$), -5.4 (-Si (CH_3)$_2$) ppm. Anal. Calcd. for $C_{19}H_{30}O_4Si$: C, 65.10; H, 8.63. Found: C, 65.25; H, 8.55. MALDI-MS $[M + Na]^+$ calcd. for ($C_{19}H_{30}O_4Si$) 350.42, found 350.55

3-O-Benzyl-6-*O*-*tert*-butyldiphenylsilyl-D-galactal (34).

$[\alpha]_D^{25}$ - 3 (*c* 1.9, $CHCl_3$); ^1H NMR (400 MHz, $CDCl_3$): δ 7.90-7.30 (aromatic H), 6.50 (d, *J* = 6.0 Hz, 1H, H-1), 4.84 (d, *J* = 6.0 Hz, 1H, H-2), 4.82-4.75 (AB, *J* = 12.0 Hz, 2H, -C*H*₂Ph), 4.35 (s, 2H), 4.23 (m, 1H), 4.15-4.00 (overlapped signals, 2H), 2.73 (s, 1 H), 1.22 (s, 9 H, *t*-butyl protons) ppm. ^{13}C NMR (100 MHz, $CDCl_3$): δ 144.9 (C-1), 137.7, 135.5, 133.1, 129.7-127.7 (aromatic signals), 99.5 (C-2), 76.5 (C-5), 70.9 (-CH_2Ph), 70.4 (C-3), 62.6, 62.5 (C-4, C-6), 26.7 (-SiC(CH_3)₃), 19.1 (-Si*C*(CH_3)₃) ppm. Anal. Calcd. for $C_{29}H_{34}O_4Si$: C, 73.38; H, 7.22. Found: C, 73.20; H, 7.40. MALDI-MS $[M + Na]^+$ calc. for ($C_{29}H_{34}O_4Si$) 497.21, found 497.45.

Allyl 3-*O*-benzyl-6-*O*-*tert*-butyldimethylsilyl-α-D-galactopyranoside (35).

$[\alpha]_D^{25}$+81 (*c* 1.6, $CHCl_3$);^1H NMR (400 MHz, $CDCl_3$): δ 7.40-7.20 (aromatic H), 6.00-5.90 (m, 1H, -C*H*=CH_2), 5.28 (bd, *J* = 1.6 and 17.2 Hz, 1H, -CH=CH_aH_b), 5.20 (bd, *J* = 1.6 and 10.4 Hz, 1H, -CH=CH_aH_b), 4.95 (d, *J* = 4.0 Hz, 1H, H-1), 4.76 and 4.71 (2 x d, AB, *J* = 11.6 Hz, 2 H, -C*H*₂Ph), 4.19 (bdd, *J* = 5.2 and 12.8 Hz, 1H, -C*Ha*HbCH=CH_2), 4.05 (d, *J* = 3.2 Hz, 1H, H-4), 4.02 (dd, *J* = 6.6 and 12.8 Hz, 1H, -CHa*Hb*CH=CH_2), 3.84 (dd, *J* = 5.6 and 9.6 Hz, 1H, H-6a), 3.80-3.70 (overlapped signals, 2H), 3.63 (dd, *J* = 2.8 and 9.6 Hz, 1H, H-3), 2.63 (bs, exchangeable, 1H), 2.23 (bs, exchangeable, 1H), 0.89 (s, 9H, *t*-butyl protons), 0.07 (s, 6H, -Si(CH_3)₂). ^{13}C NMR (100 MHz, $CDCl_3$): δ 137.9, 128.5, 127.9, 127.8 (aromatic signals), 133.6 (CH_2=*C*H-), 117.8 (*C*H₂=CH-), 97.6 (C-1), 78.7 (C-3), 72.0 (-CH_2Ph), 70.2 (C-5), 68.6 (C-2), 68.4 (CH_2=CH*C*H₂-), 66.9 (C-4), 62.5 (C-6), 25.8 (-SiC(*C*H₃)₃), 18.2 (-Si*C*(CH_3)₃), -5.4 (-Si (CH_3)₂). Anal. Calcd. for $C_{22}H_{36}O_6Si$: C, 62.23; H, 8.55. Found: C, 62.10; H, 8.60. MALDI-MS $[M + Na]^+$ calcd. for ($C_{22}H_{36}O_6Si$) 447.22, found 447.40.

References of known products

1. Lee, D.; Taylor, M. S. *J. Am. Chem. Soc.* **2011**, *133*, 3724-3727.
2. El-Badri, M. H.; Willenbring, D.; Tantillo, D. J.; Gervay-Hague, J. *J. Org. Chem.* **2007**, *72*, 4663-4672.
3. Belakhov, V.; Dovgolevsky, E.; Rabkin, E.; Shulami, S.; Shoham, Y.; Baasov, T. *Carbohydr. Res.* **2004**, *339*, 385-392.
4. Bartoszewicz, A.; Kalek, M.; Stawinski, J. *Tetrahedron* **2008**, *64*, 8843-8850.
5. Kishore, G. D. K.; Baskaran, S. *J. Org. Chem.* **2005**, *70*, 4520-4523.
6. Moitessier, N.; Englebienne, P.; Chapleur, Y. *Tetrahedron* **2005**, *61*, 6839-6853.
7. Boto, A.; Hernandez, D.; Hernandez, R.; Suarez, E. *J. Org. Chem.* **2006**, *72*, 1938-1948

8. Krylov, V. B.; Argunov, D. A.; Vinnitskiy, D. Z.; Verkhnyatskaya, S. A.; Gerbst, A. G.; Ustyuzhanina, N. E.; Dmitrenok, A. S.; Huebner, J.; Holst, O.; Siebert, H.-C.; Nifantiev, N. E. *Chem. Eur. J.* **2014**, *20*, 16516-16522.

9. Levecque, P.; Gammon, D. W.; Kinfe, H. H.; Jacobs, P.; De Vos, D.; Sels, B. *Adv. Syn. Catal.* **2008**, *350*, 1557-1568.

10. Davis, B. G.; Nash, R. J.; Watson, A. A.; Smith, C.; Fleet, G. W. J. *Tetrahedron* **1999**, *55*, 4501-4520.

11. Lam, S. N.; Gervay-Hague, J. *Carbohydr. Res.* **2002**, *337*, 1953–1965.

12. Arias-Perez, M. S.; Lopez, M. S.; Santos, M. J. *J. Chem. Soc. Perkin Trans* **2002**, 1549-1552.

13. Kumar, P. S.; Kumar, G. D. K.; Baskaran, S. *Eur. J Org. Chem.* **2008**, 6063-6067.

14. Chung, M.-K.; Orlova, G.; Goddard, J. D.; Schlaf, M.; Harris, R.; Beveridge, T. J.; White, G.; Hallett, F. R. J. *Am. Chem. Soc.* **2002**, *124*, 10508-10518.

15. Lee, D.; Taylor, M. S. *Org. Biomol. Chem*. **2013**, *11*, 5409–5412.

16. Chen, N.; Xie, J. *Org. Biomol. Chem.* **2016**, *14*, 1102-1110.

17. Nakagawa, Y.; Doi, T.; Taketani, T.; Takegoshi, K.; Igarashi, Y.; Ito, Y. *Chem. Eur. J.* **2013**, *19*, 10516-10525.

18. Francais, A.; Urban, D. ; Beau, J.-M. *Angew. Chem. Int. Ed.***2007**, *46*, 8662-8665;

19. Wang, C.-C.; Kulkarni, S. S.; Lee, J.-C.; Luo, S.-Y.; Hung, S.-C. *Nat. Protoc.***2008**, *3*, 97-113.

20. Irmak, M.; Groschner, A.; Boysen, M. M. K. *Chem. Commun.* **2007**, 177-179.

21. Abragam Joseph, A.; Dhurandhare, V. M.; Chang, C. W.: Verma, V. P.: Mishra, G. P.; Ku, C. C.; Lin, C.-C.; Wang, C.-C.*Chem. Commun*. **2015**, *51*, 104-106.

22. Lim, J.; Grove, B. C.; Roth, A.; Breaker, R. R. *Angew. Chem. Int. Ed.* **2006**, *45*, 6689-6693.

23. George, J.; Reddy, B. V. S. *Org. Biomol. Chem.* **2012**, *10*, 4731-4738.

24. Minuth, T.; Irmak, M.; Groschner, A.; Lehnert, T.; Boysen, M. M. K. *Eur. J. Org. Chem.* **2009**, 997-1008.

25. Saikam, V.; Dara, S.; Yadav, M.; Singh, P.; Vishwakarma, R. A. *J. Org. Chem*. **2015**, *80*, 11916-11925.

26. Chan, L.; Taylor, M. S. *Org. Lett.* **2011**, *13*, 3090-3093.

27. Ren, B.; Ramstroem, O.; Zhang, Q.; Ge, J.; Dong, H *Chem. Eur. J.* **2016**, *22*, 2481-2486.

28. Novikov, Y. Y.; Sampson, P. *J. Org. Chem.* **2005**, *70*, 10247-10259.

29. Herradon, B.; Morcuende, A.; Valverde, S. *Synlett* **1995**, *5*, 455-458.

30. Bieg, T.; Kral, K.; Paszkowska, J.; Szeja, W.; Wandzik, I. *J. Carbohyd. Chem.* **2012**, *31*, 593-601.

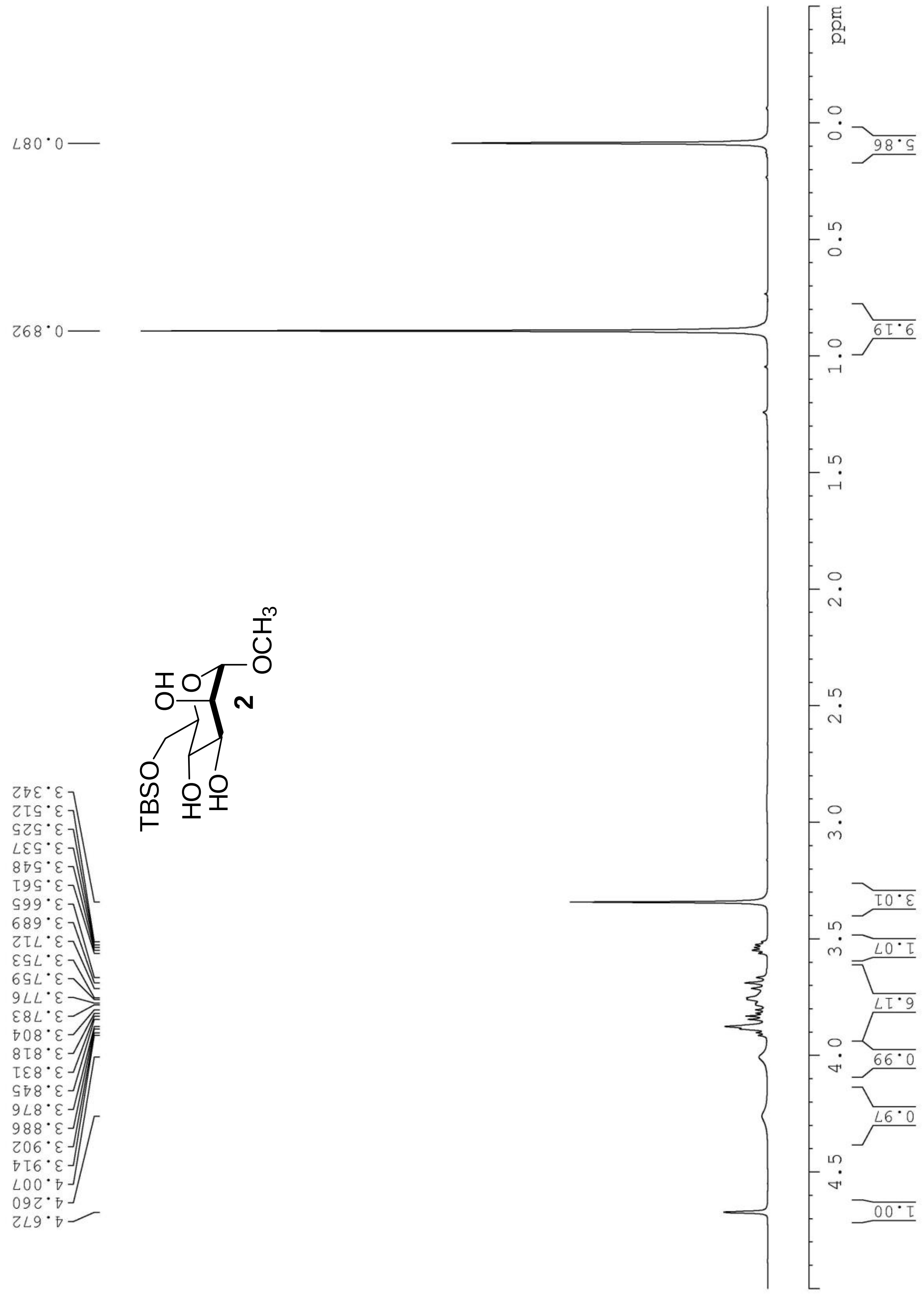
0.087
0.892
3.342
3.512
3.525
3.537
3.548
3.561
3.665
3.689
3.712
3.753
3.759
3.776
3.783
3.804
3.818
3.831
3.845
3.876
3.886
3.902
3.914
4.007
4.260
4.672
TBSO
HO
HO
OH
O
OCH3
2
5.86
9.19
3.01
1.07
6.17
0.99
0.97
1.00
ppm
0.0
0.5
1.0
1.5
2.0
2.5
3.0
3.5
4.0
4.5

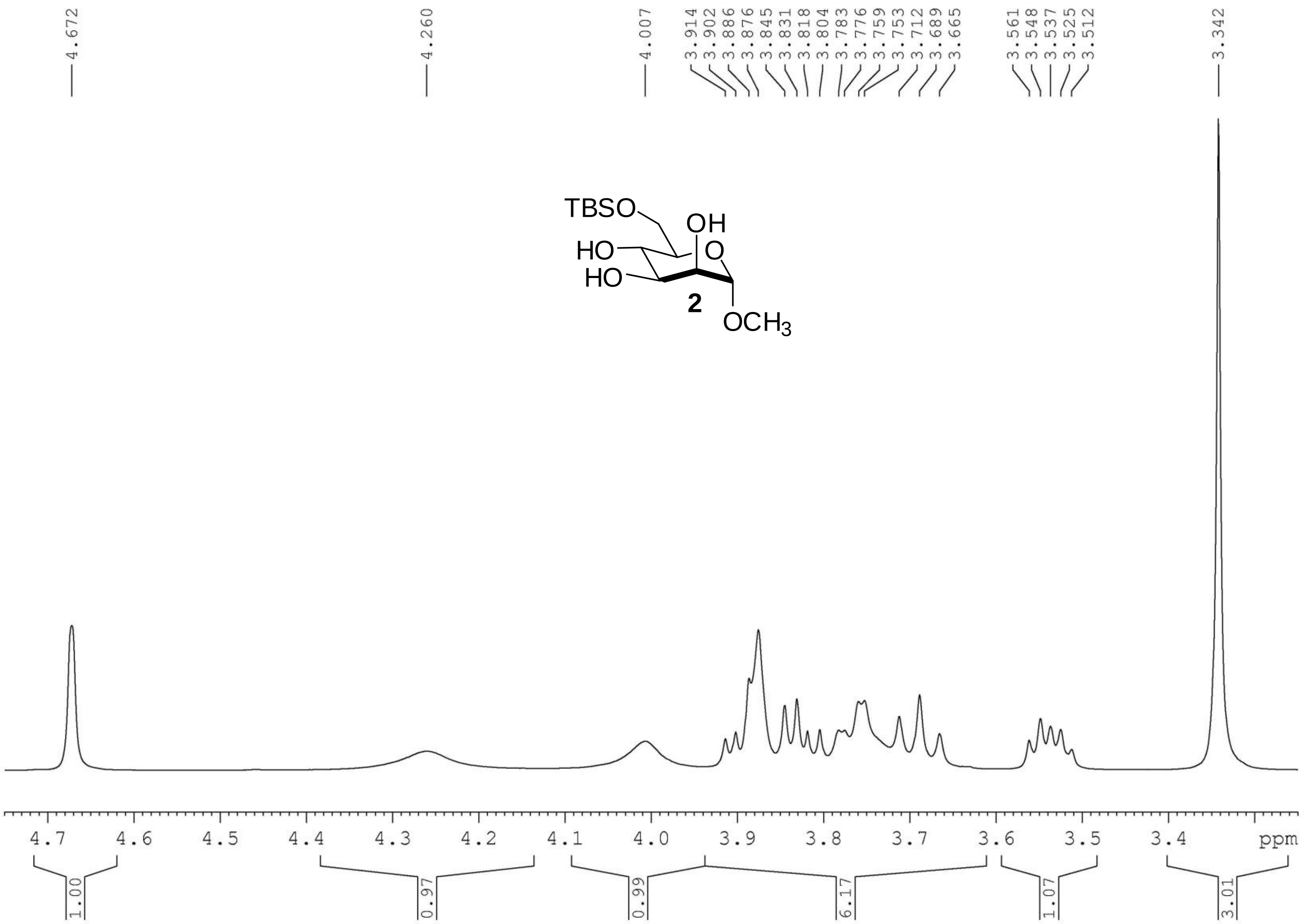
4.672
4.260
4.007
3.914
3.902
3.886
3.876
3.845
3.831
3.818
3.804
3.783
3.776
3.759
3.753
3.712
3.689
3.665
3.561
3.548
3.537
3.525
3.512
3.342
TBSO
OH
HO
O
HO
2
OCH3
4.7
4.6
4.5
4.4
4.3
4.2
4.1
4.0
3.9
3.8
3.7
3.6
3.5
3.4
ppm
1.00
0.97
0.99
6.17
1.07
3.01

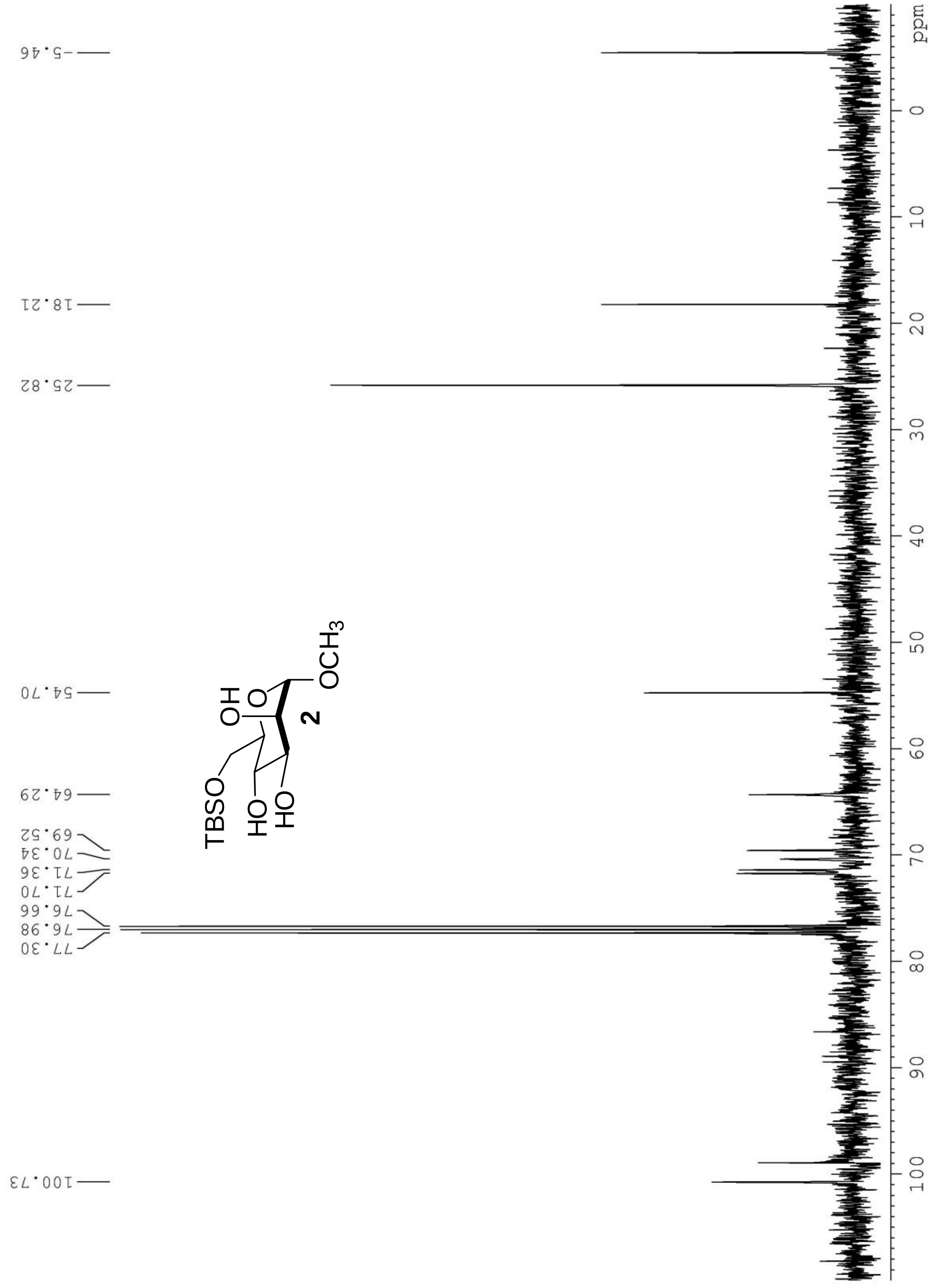
-5.46
18.21
25.82
54.70
64.29
69.52
70.34
71.36
71.70
76.66
76.98
77.30
100.73
TBSO
HO
HO
OH
O
OCH3
2
ppm

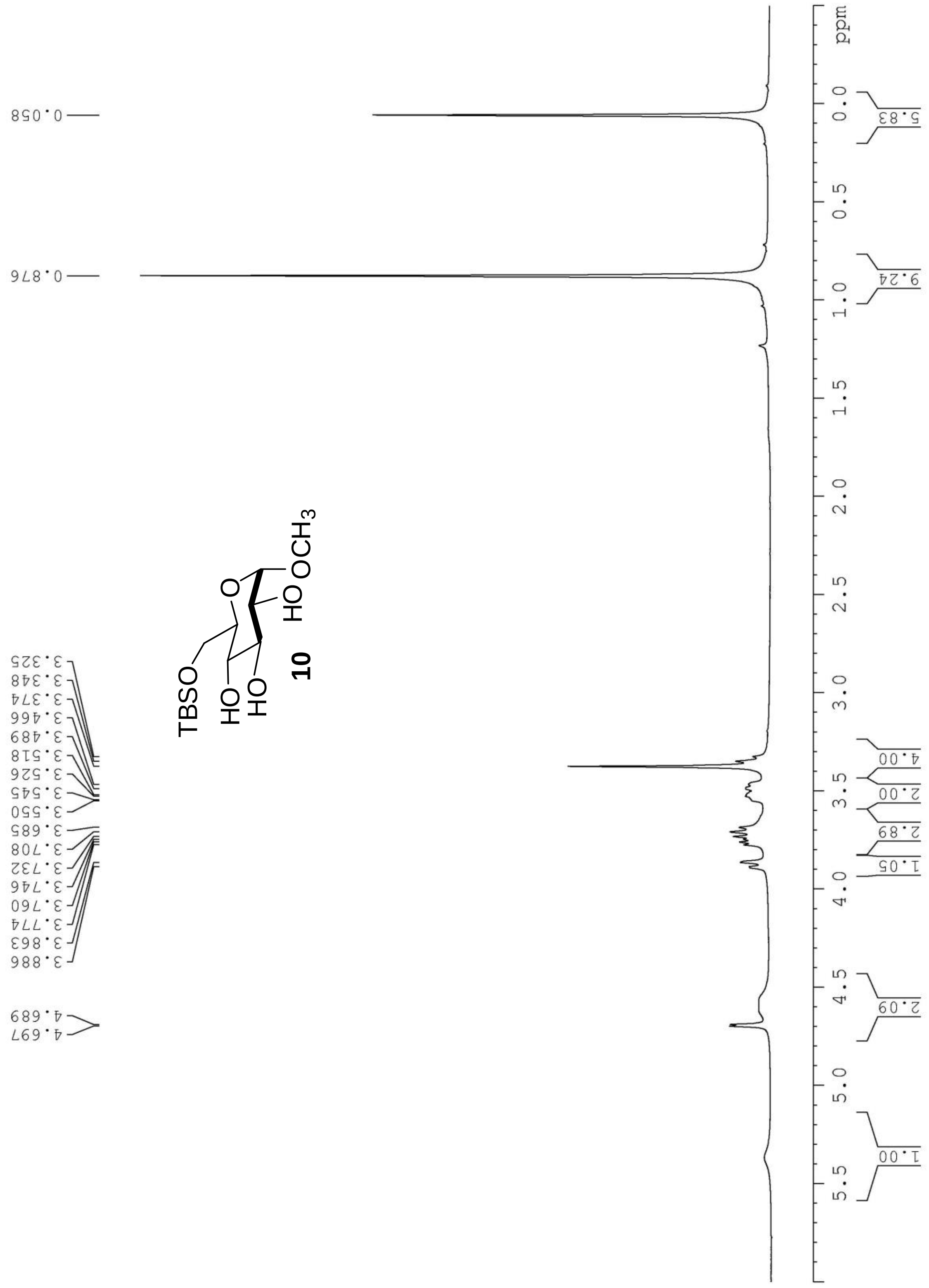
TBSO
HO
HO
HO
O
OCH3
10
ppm
0.058
0.876
3.325
3.348
3.374
3.466
3.489
3.518
3.526
3.545
3.550
3.685
3.708
3.732
3.746
3.760
3.774
3.863
3.886
4.689
4.697
5.83
9.24
4.00
2.00
2.89
1.05
2.09
1.00

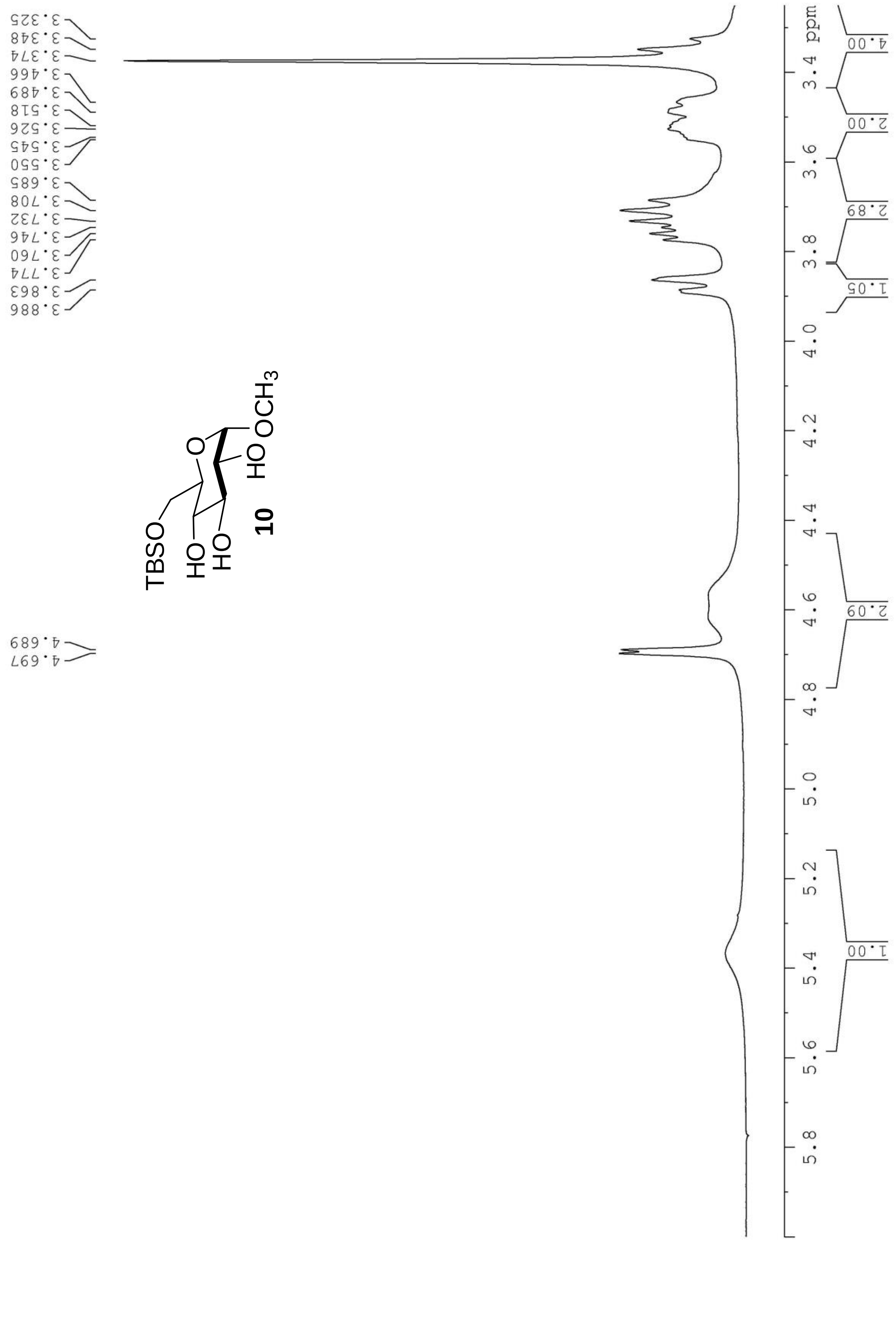
TBSO
HO
HO
HO
OCH3
10
ppm

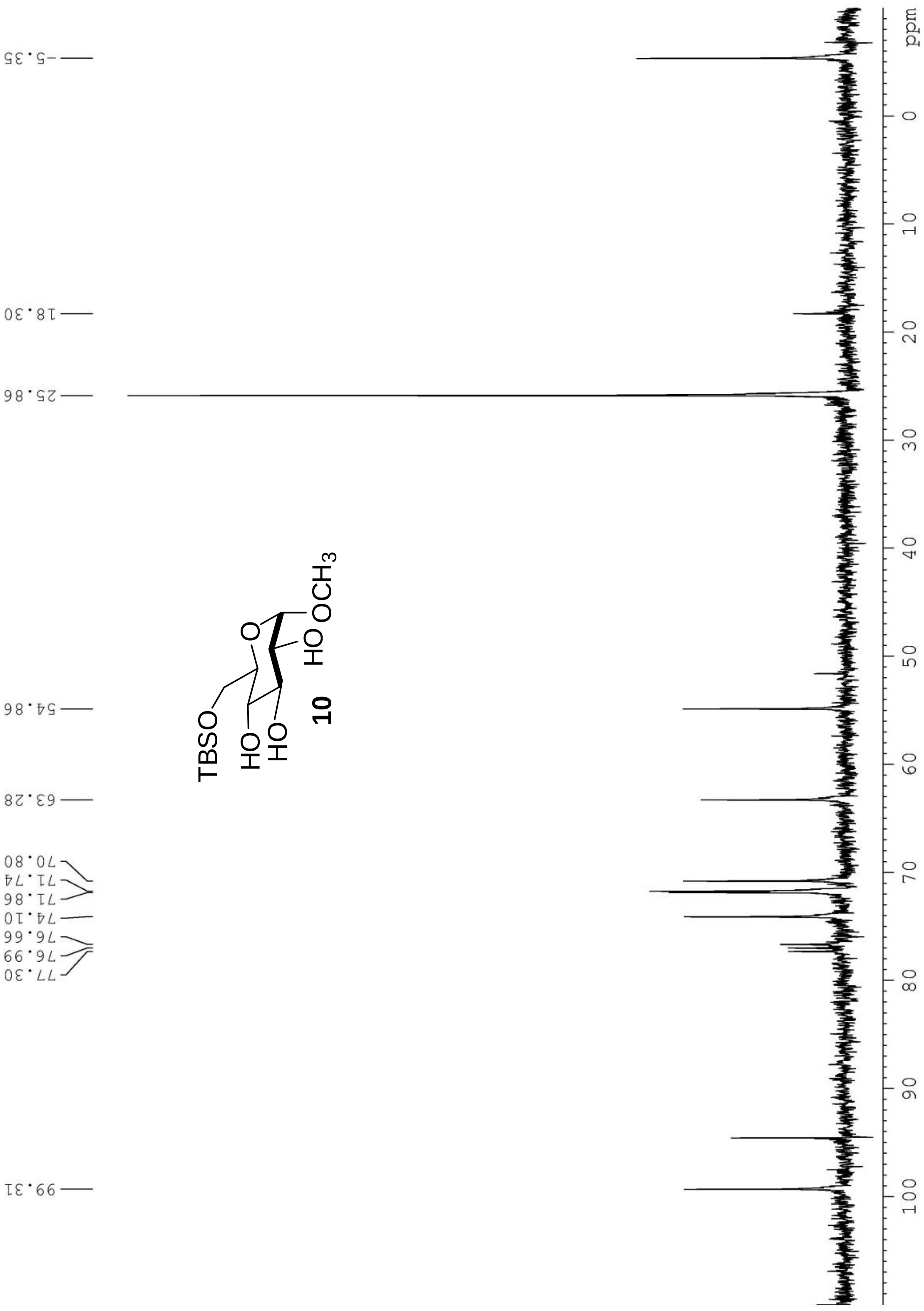
-5.35
18.30
25.86
54.86
63.28
70.80
71.74
71.86
74.10
76.66
76.99
77.30
99.31
ppm
0
10
20
30
40
50
60
70
80
90
100
TBSO
HO
HO
HO
O
OCH_3
10

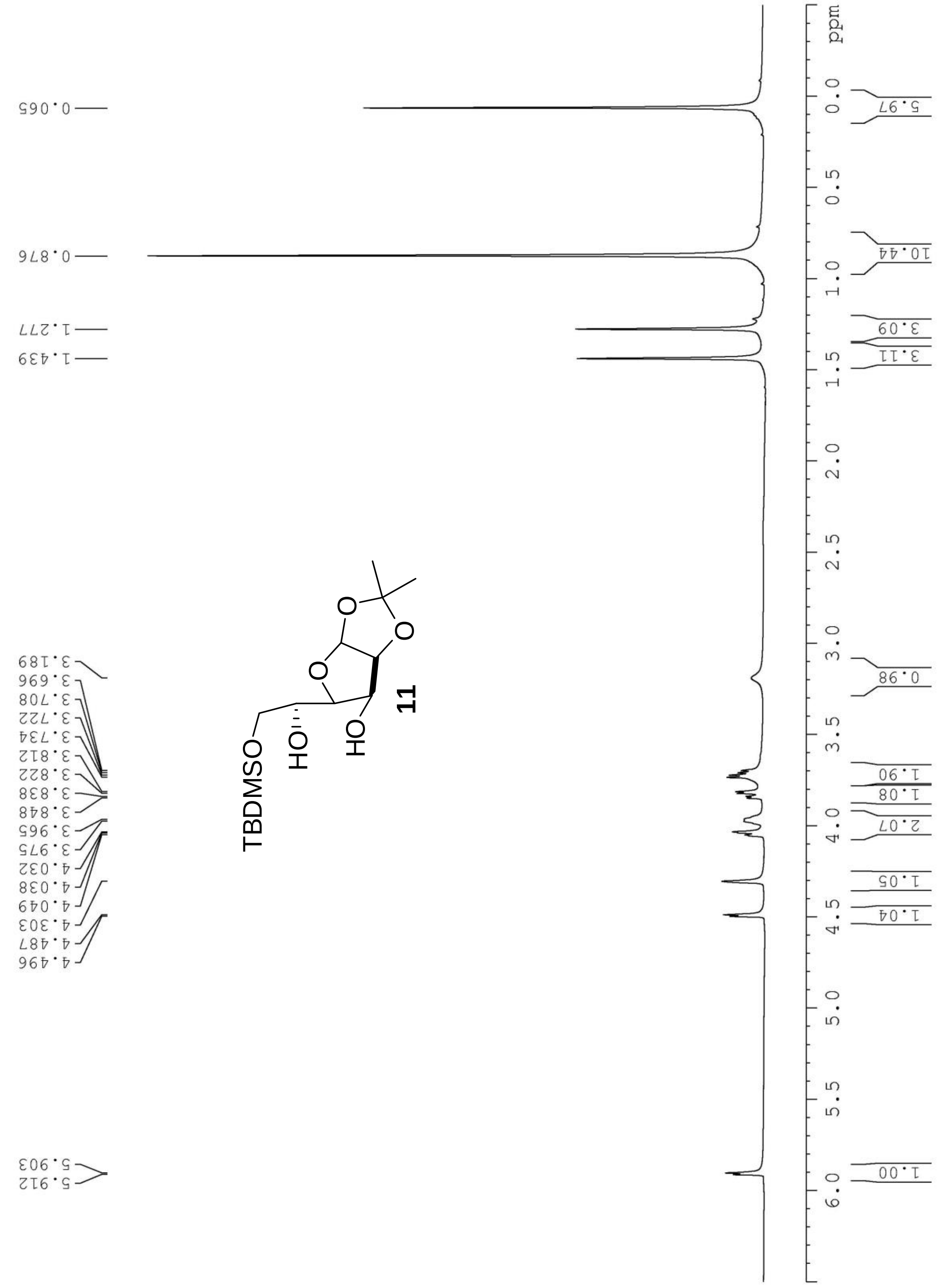
0.065
0.876
1.277
1.439
3.189
3.696
3.708
3.722
3.734
3.812
3.822
3.838
3.848
3.965
3.975
4.032
4.038
4.049
4.303
4.487
4.496
5.903
5.912
TBDMSO
HO
O
11
ppm
5.97
10.44
3.09
3.11
0.98
1.90
1.08
2.07
1.05
1.04
1.00

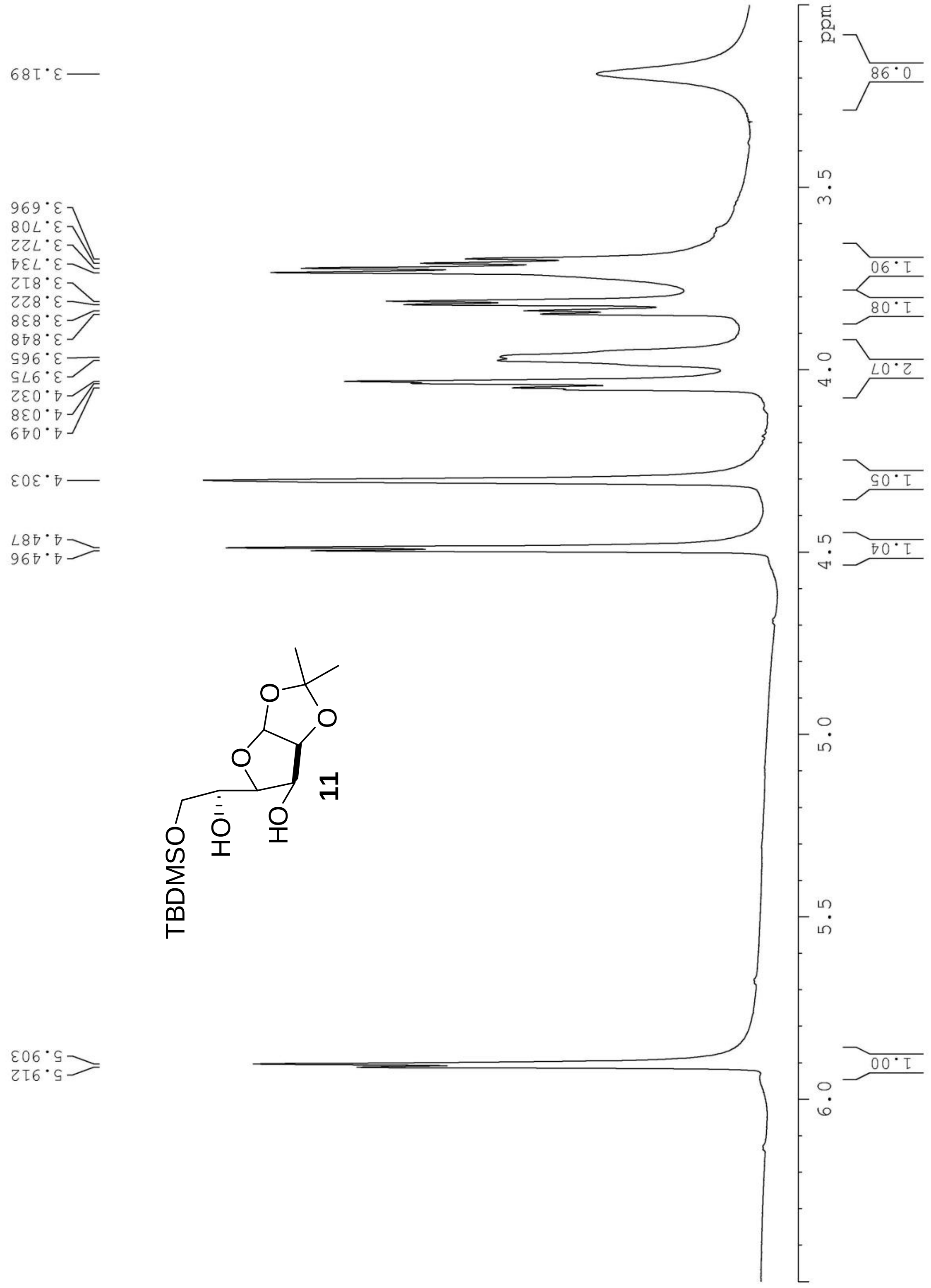
3.189
3.696
3.708
3.722
3.734
3.812
3.822
3.838
3.848
3.965
3.975
4.032
4.038
4.049
4.303
4.487
4.496
5.903
5.912
TBDMSO
HO
HO
O
O
O
11
ppm
3.5
4.0
4.5
5.0
5.5
6.0
0.98
1.90
1.08
2.07
1.05
1.04
1.00

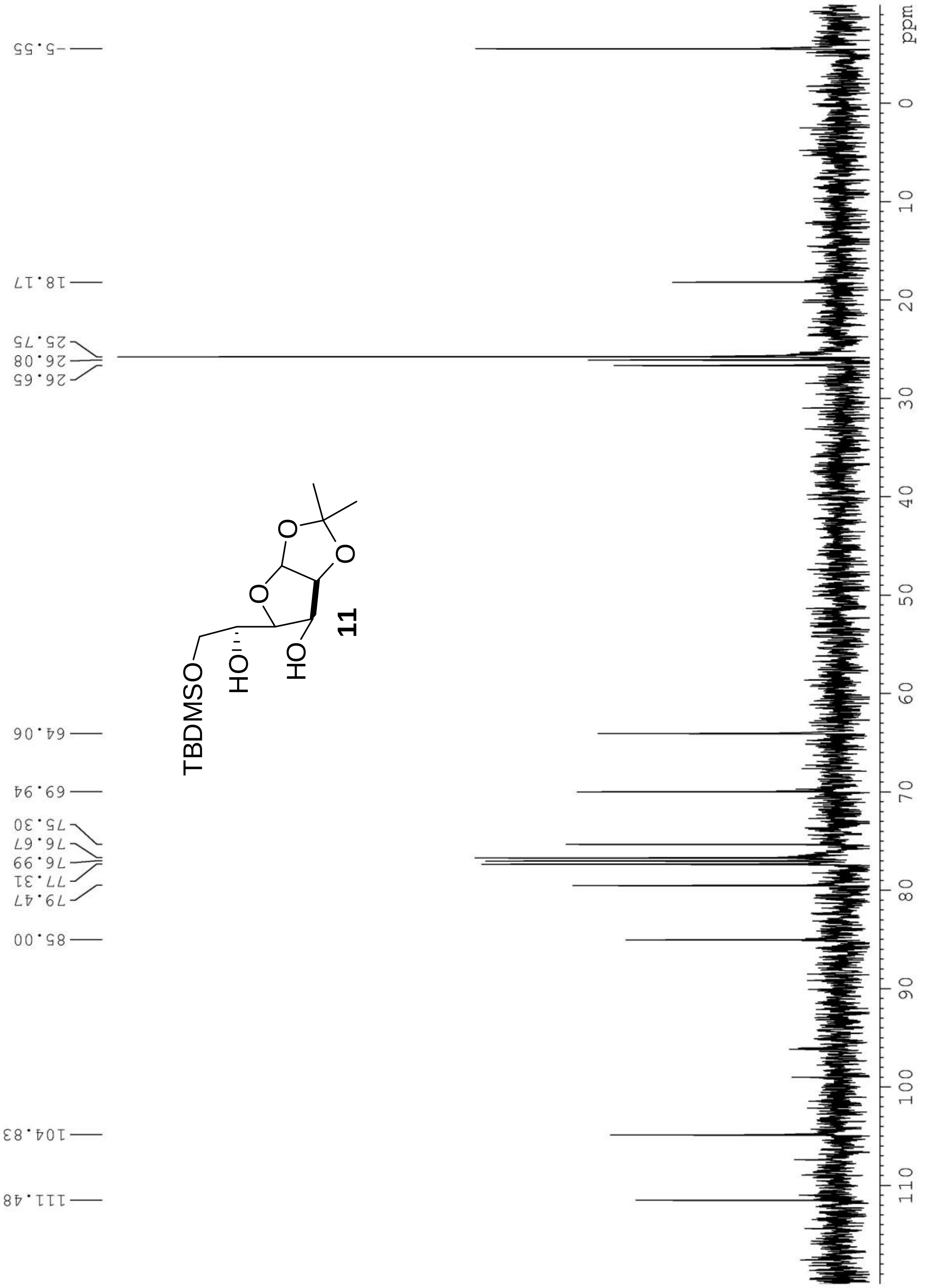
-5.55
18.17
25.75
26.08
26.65
64.06
69.94
75.30
76.67
76.99
77.31
79.47
85.00
104.83
111.48
ppm
TBDMSO
HO
HO
11

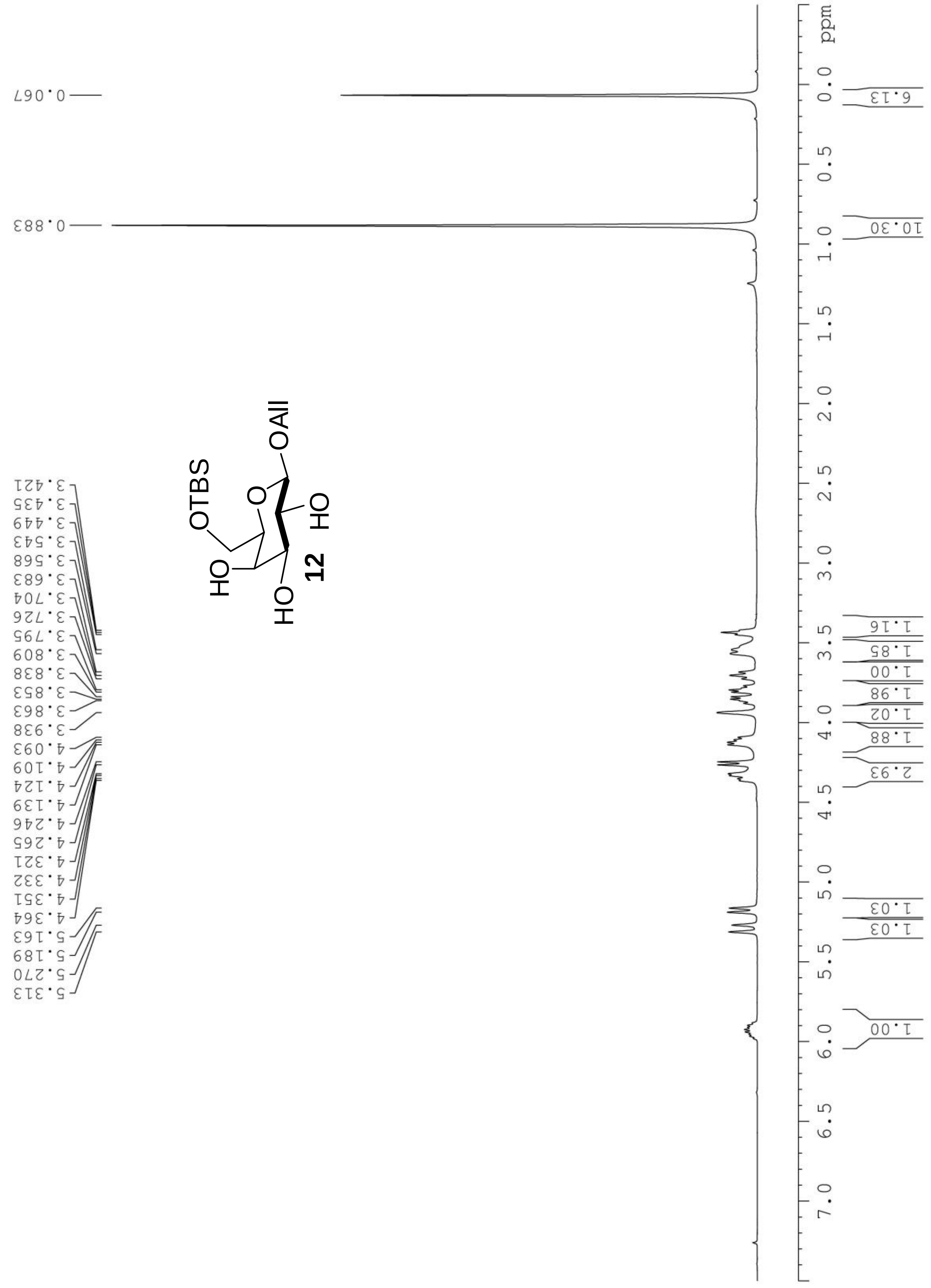
OTBS
O
OAll
HO
HO
HO
12
0.067
0.883
3.421
3.435
3.449
3.543
3.568
3.683
3.704
3.726
3.795
3.809
3.838
3.853
3.863
3.938
4.093
4.109
4.124
4.139
4.246
4.265
4.321
4.332
4.351
4.364
5.163
5.189
5.270
5.313
ppm
0.0
0.5
1.0
1.5
2.0
2.5
3.0
3.5
4.0
4.5
5.0
5.5
6.0
6.5
7.0
6.13
10.30
1.16
1.85
1.00
1.98
1.02
1.88
2.93
1.03
1.03
1.00

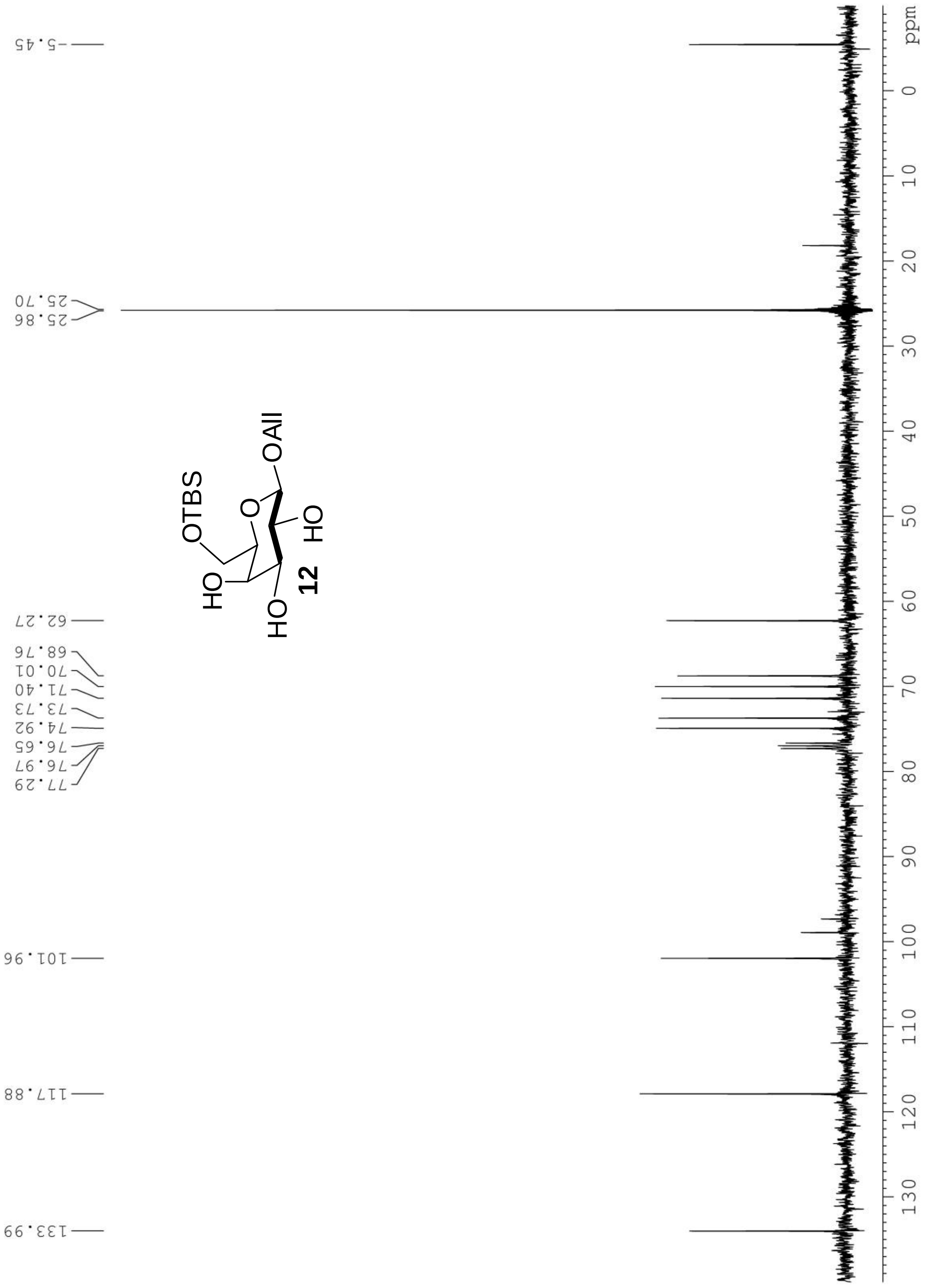
-5.45
25.70
25.86
62.27
68.76
70.01
71.40
73.73
74.92
76.65
76.97
77.29
101.96
117.88
133.99
OTBS
O
OAll
HO
HO
HO
12
ppm

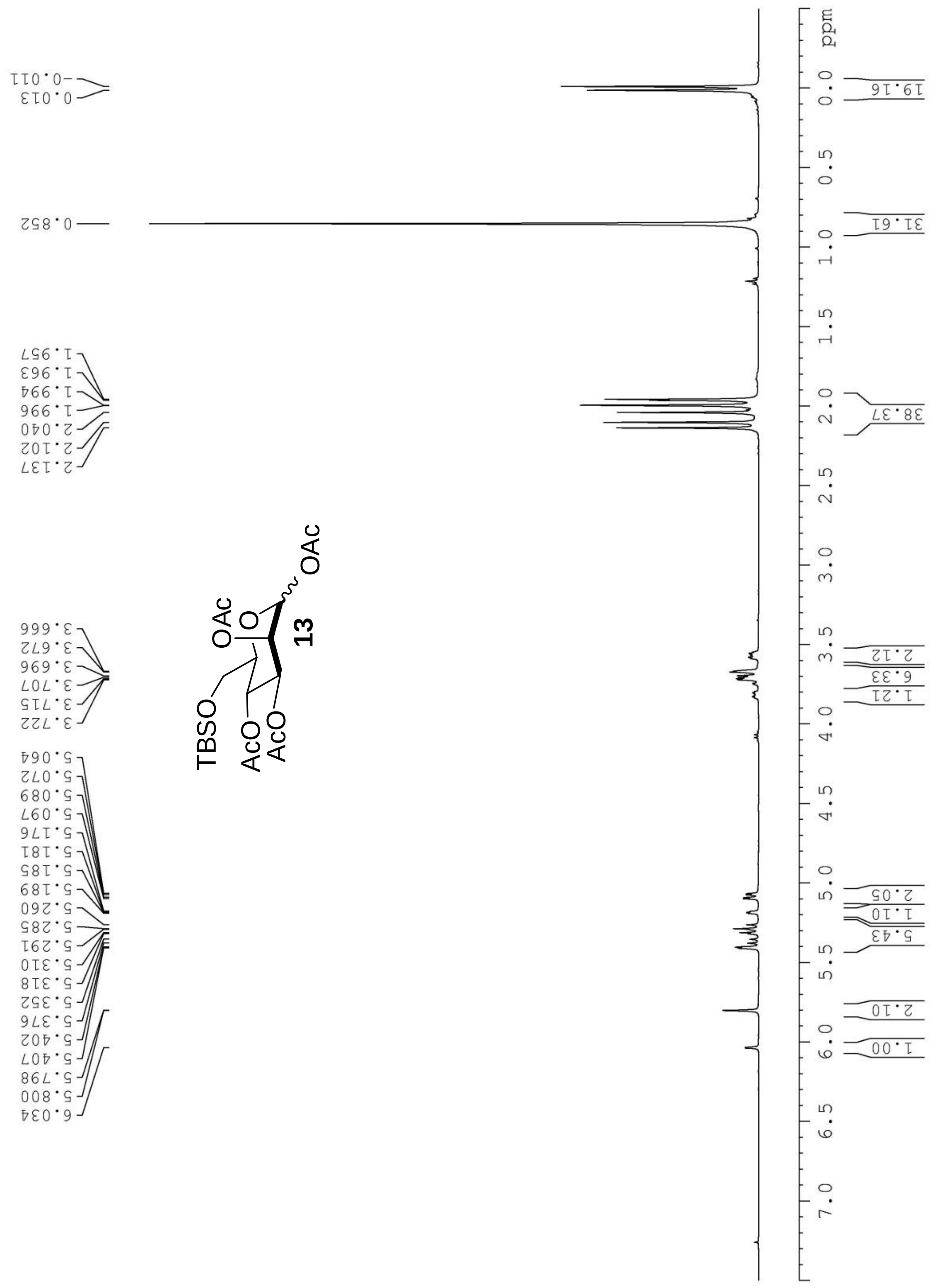
-0.011
0.013
0.852
1.957
1.963
1.994
1.996
2.040
2.102
2.137
3.666
3.672
3.696
3.707
3.715
3.722
5.064
5.072
5.089
5.097
5.176
5.181
5.185
5.189
5.260
5.285
5.291
5.310
5.318
5.352
5.376
5.402
5.407
5.798
5.800
6.034
TBSO
AcO
AcO
OAc
O
OAc
13
ppm
0.0
0.5
1.0
1.5
2.0
2.5
3.0
3.5
4.0
4.5
5.0
5.5
6.0
6.5
7.0
19.16
31.61
38.37
2.12
6.33
1.21
2.05
1.10
5.43
2.10
1.00

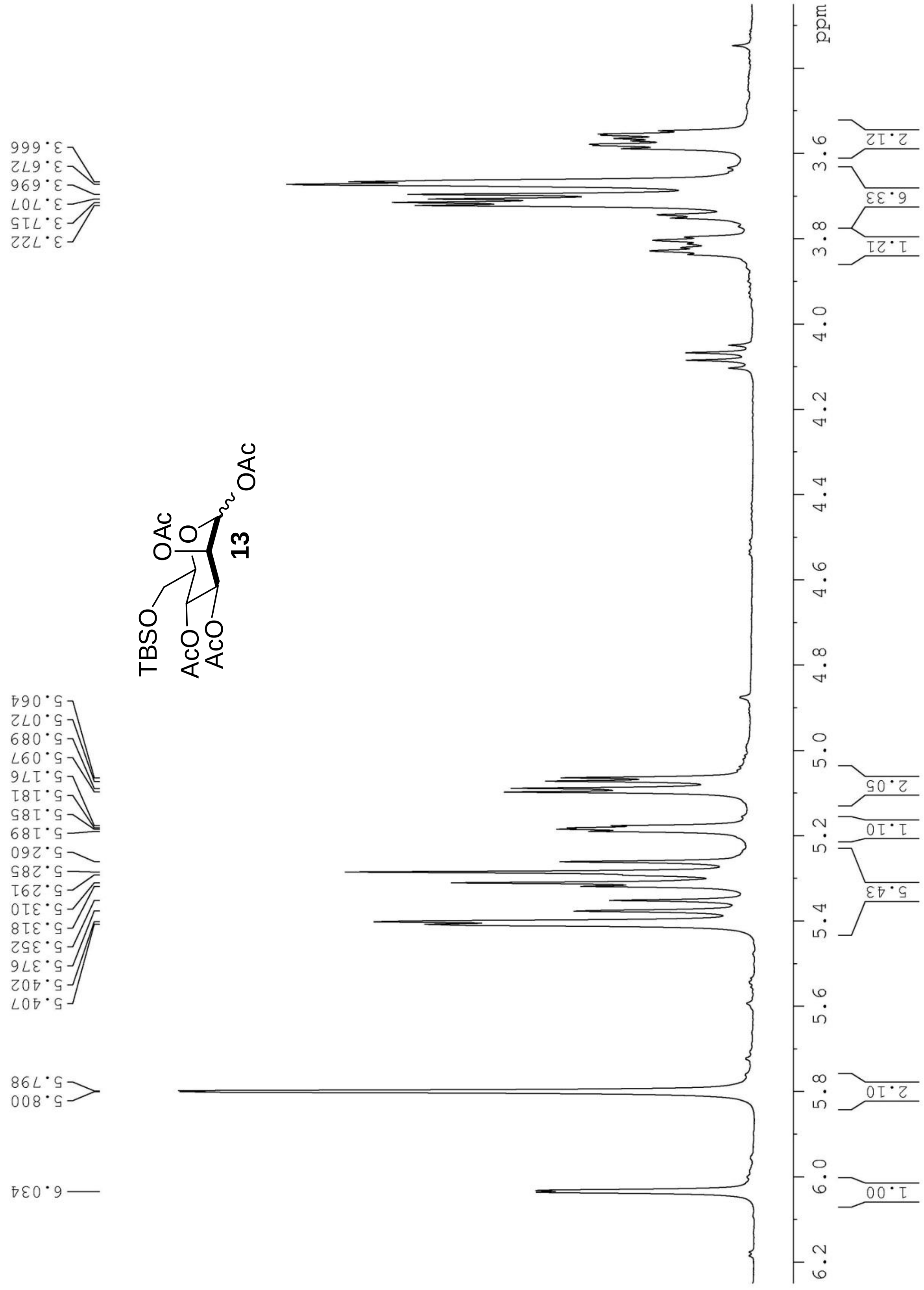
3.666
3.672
3.696
3.707
3.715
3.722
5.064
5.072
5.089
5.097
5.176
5.181
5.185
5.189
5.260
5.285
5.291
5.310
5.318
5.352
5.376
5.402
5.407
5.798
5.800
6.034
TBSO
AcO
AcO
OAc
O
OAc
13
2.12
6.33
1.21
2.05
1.10
5.43
2.10
1.00
ppm
3.6
3.8
4.0
4.2
4.4
4.6
4.8
5.0
5.2
5.4
5.6
5.8
6.0
6.2

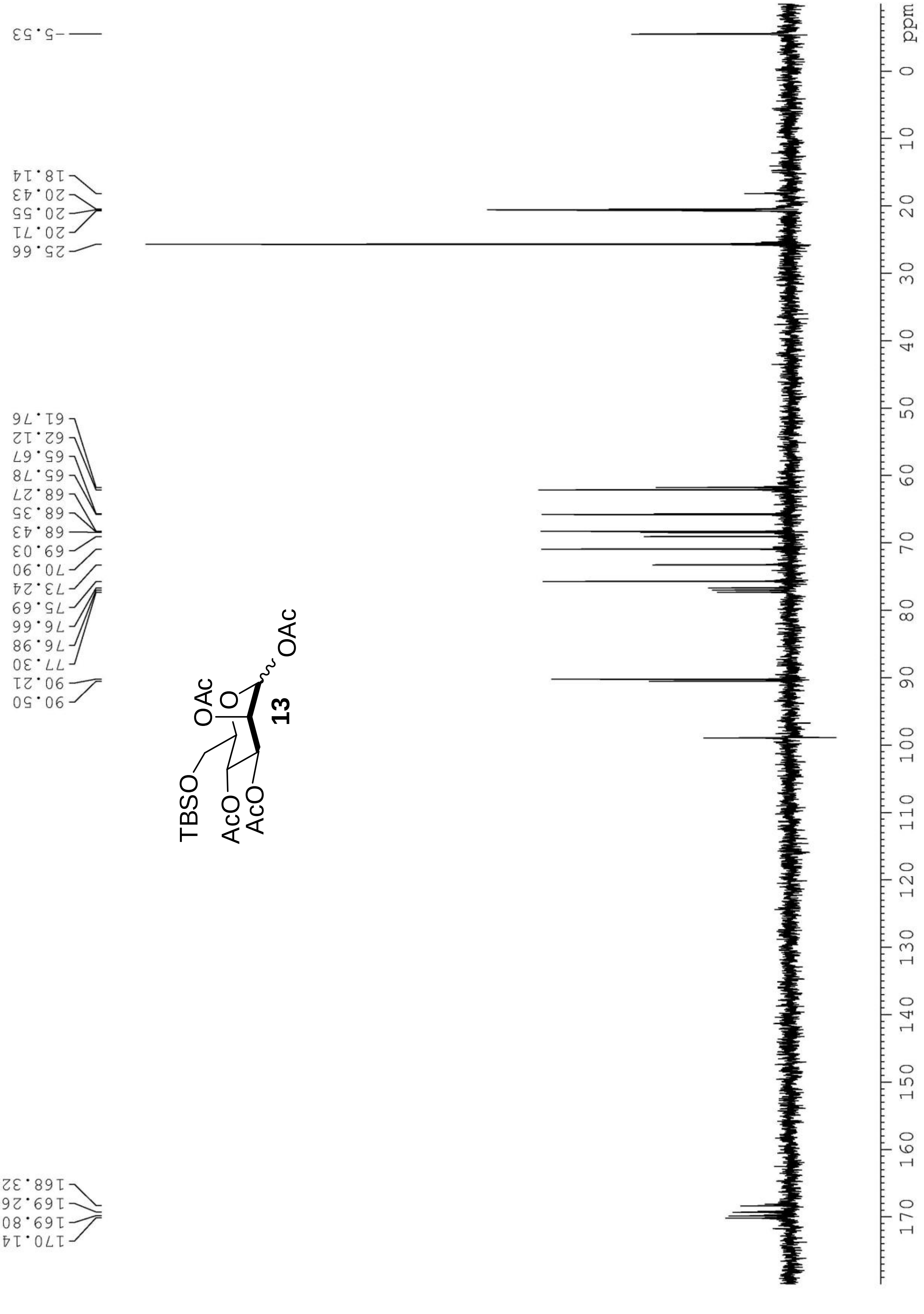
-5.53
18.14
20.43
20.55
20.71
25.66
61.76
62.12
65.67
65.78
68.27
68.35
68.43
69.03
70.90
73.24
75.69
76.66
76.98
77.30
90.21
90.50
168.32
169.26
169.80
170.14
TBSO
AcO
AcO
OAc
O
OAc
13
ppm

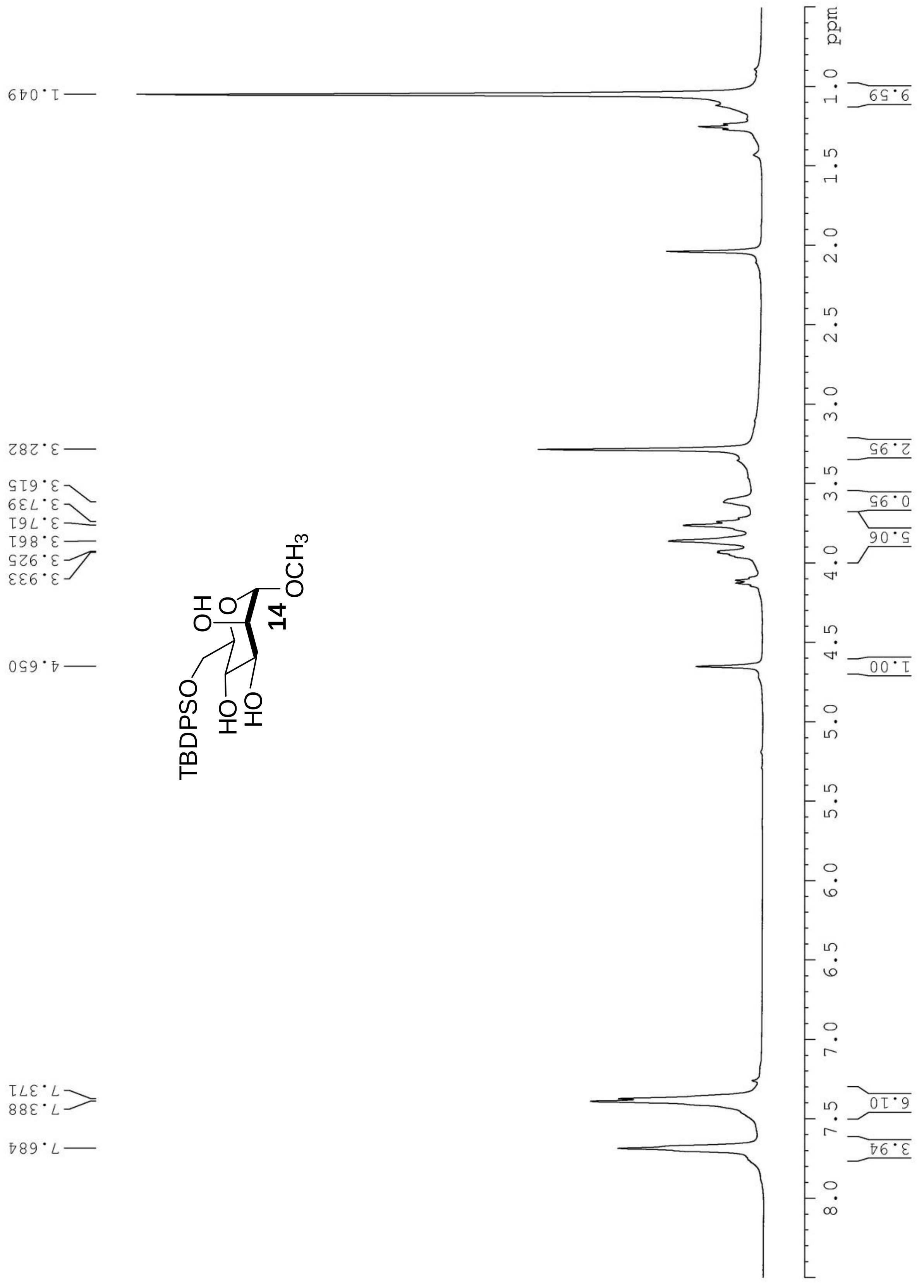
1.049
3.282
3.615
3.739
3.761
3.861
3.925
3.933
4.650
7.371
7.388
7.684
TBDPSO
HO
HO
OH
O
OCH3
14
ppm
1.0
1.5
2.0
2.5
3.0
3.5
4.0
4.5
5.0
5.5
6.0
6.5
7.0
7.5
8.0
9.59
2.95
0.95
5.06
1.00
6.10
3.94

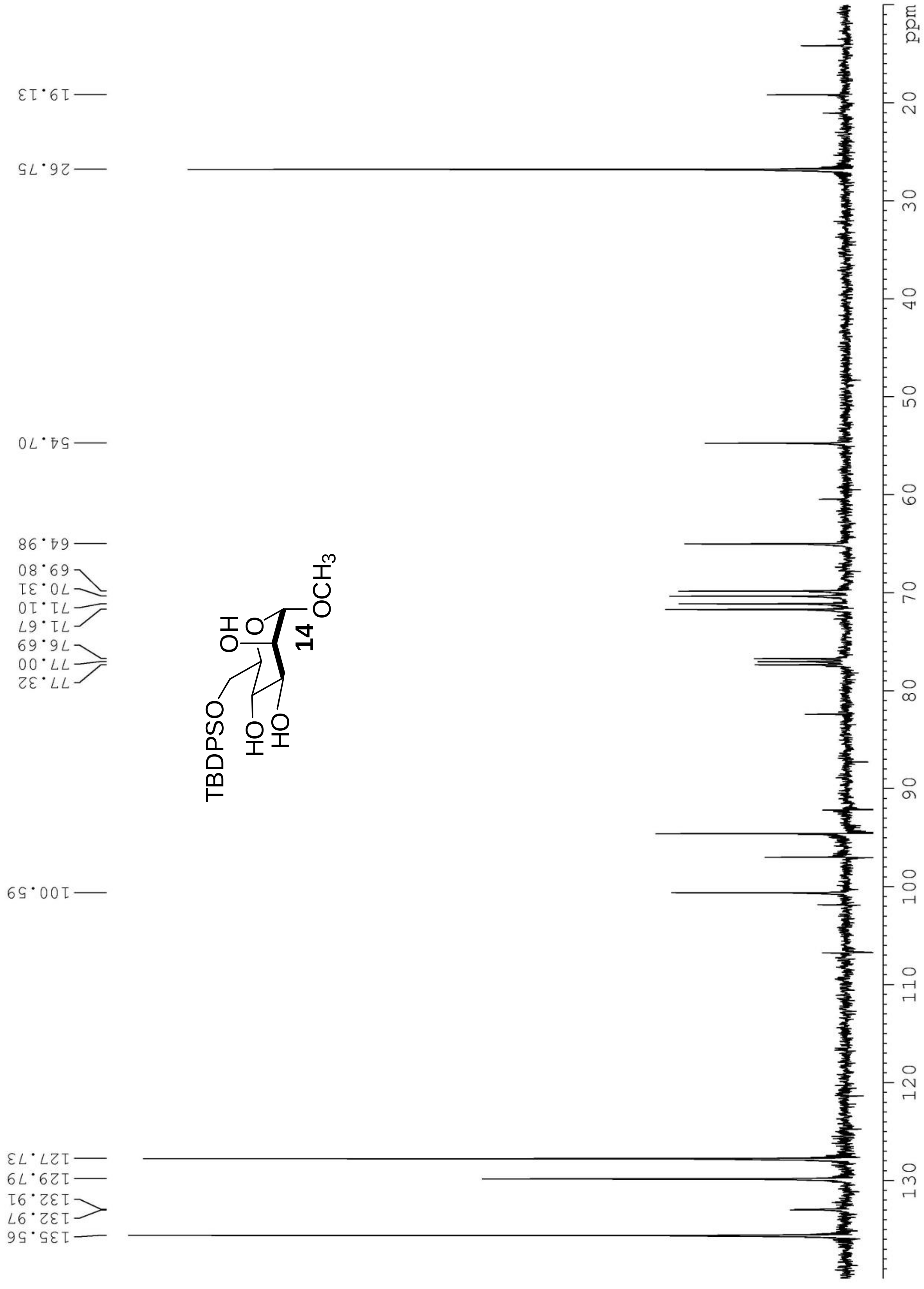
19.13
26.75
54.70
64.98
69.80
70.31
71.10
71.67
76.69
77.00
77.32
100.59
127.73
129.79
132.91
132.97
135.56
ppm
TBDPSO
HO
HO
OH
O
OCH3
14

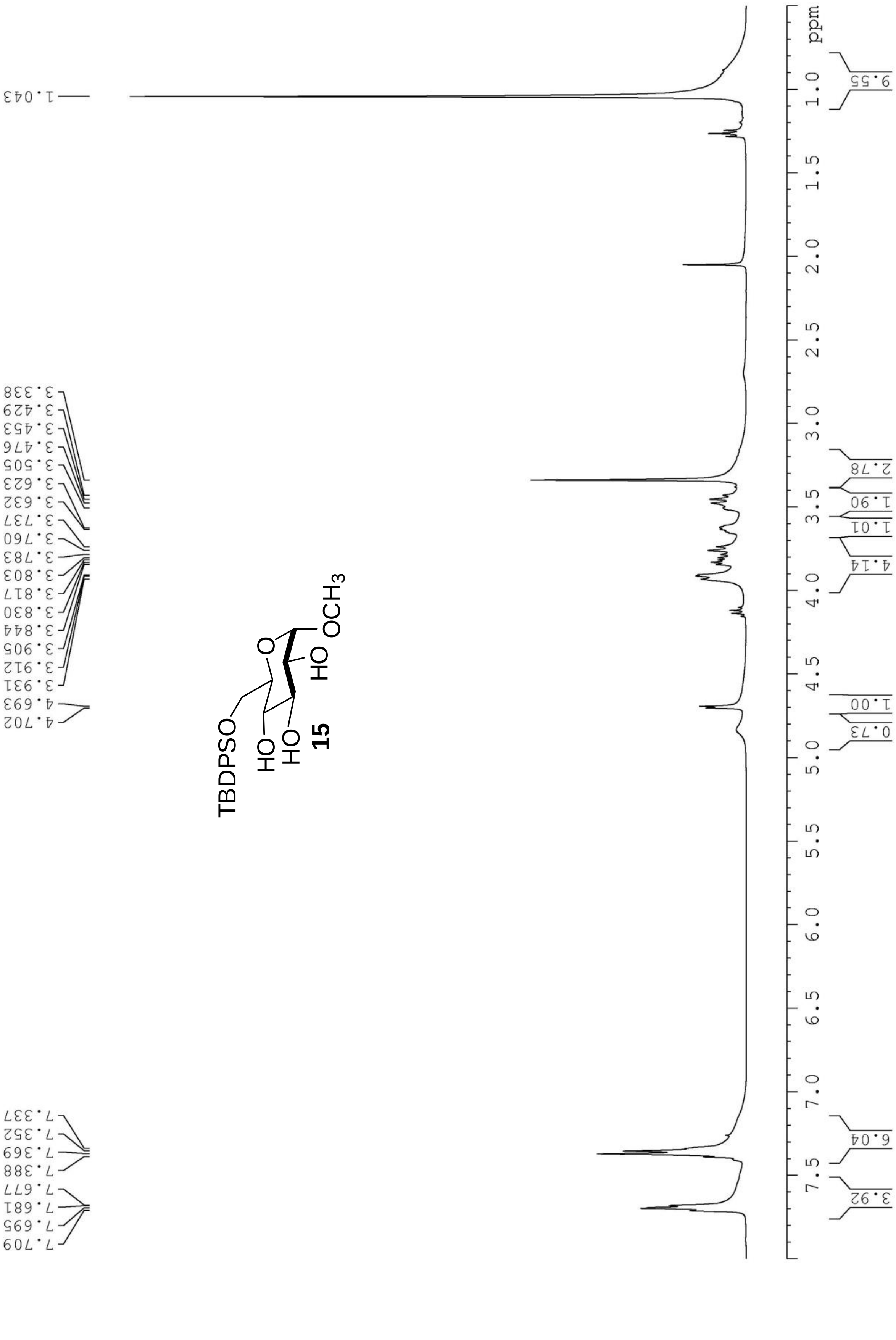

1.043
3.338
3.429
3.453
3.476
3.505
3.623
3.632
3.737
3.760
3.783
3.803
3.817
3.830
3.844
3.905
3.912
3.931
4.693
4.702
7.337
7.352
7.369
7.388
7.677
7.681
7.695
7.709
TBDPSO
HO
HO
HO
OCH3
15
9.55
2.78
1.90
1.01
4.14
1.00
0.73
6.04
3.92
ppm

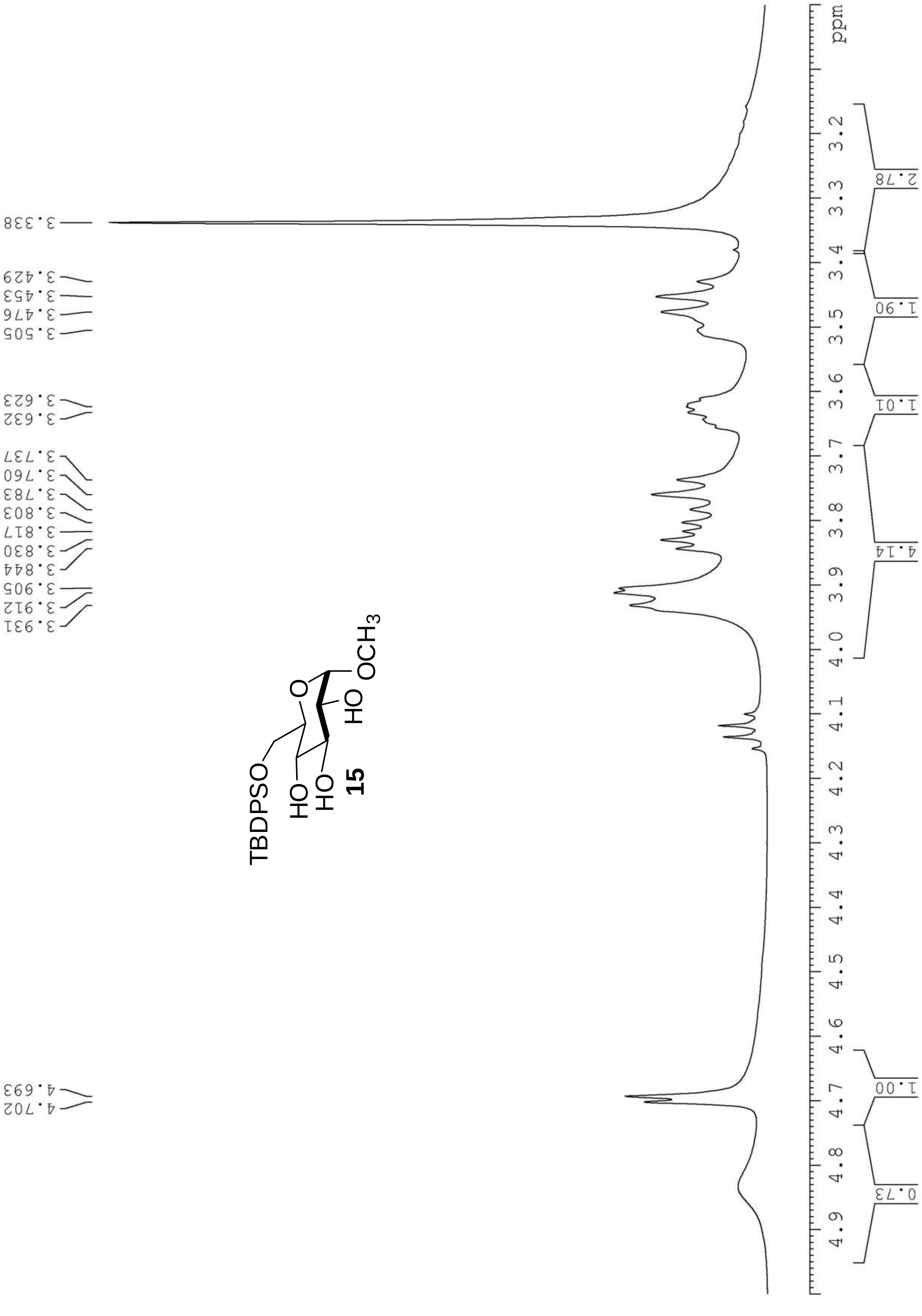

15

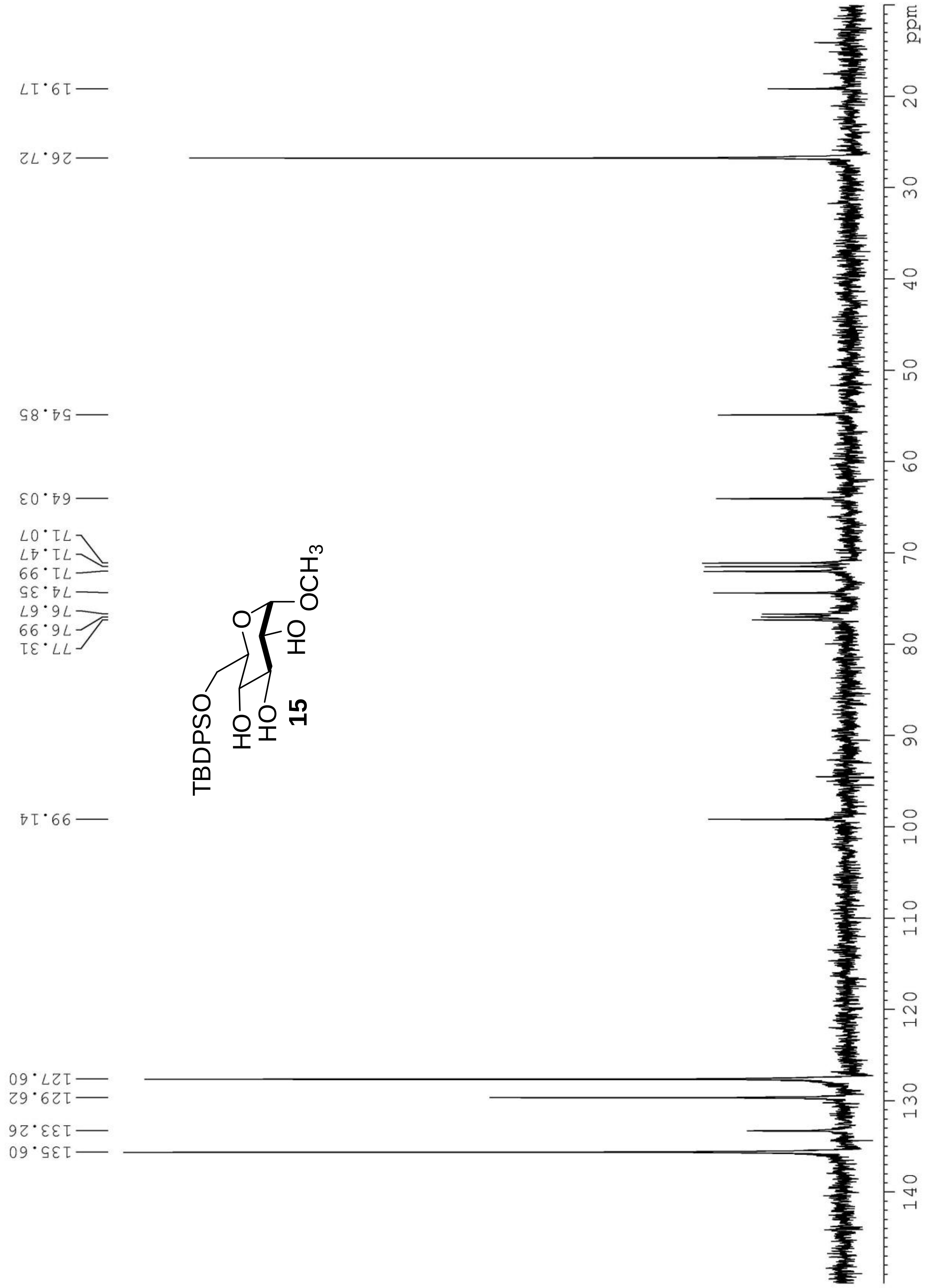

19.17
26.72
54.85
64.03
71.07
71.47
71.99
74.35
76.67
76.99
77.31
99.14
127.60
129.62
133.26
135.60
TBDPSO
HO
HO
HO
O
OCH_3
15
ppm
20
30
40
50
60
70
80
90
100
110
120
130
140

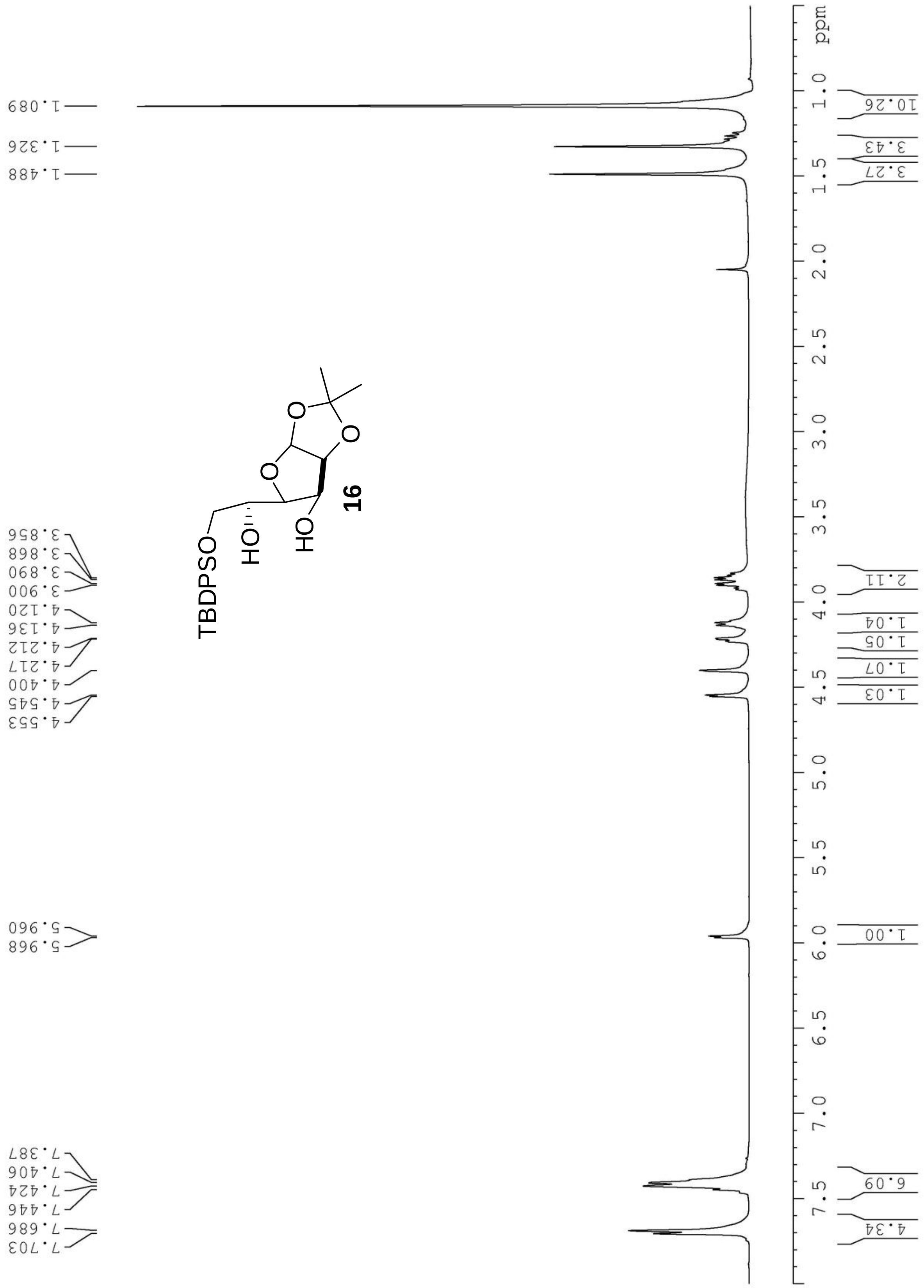
1.089
1.326
1.488
3.856
3.868
3.890
3.900
4.120
4.136
4.212
4.217
4.400
4.545
4.553
5.960
5.968
7.387
7.406
7.424
7.446
7.686
7.703
TBDPSO
HO
HO
16
10.26
3.43
3.27
2.11
1.04
1.05
1.07
1.03
1.00
6.09
4.34
ppm

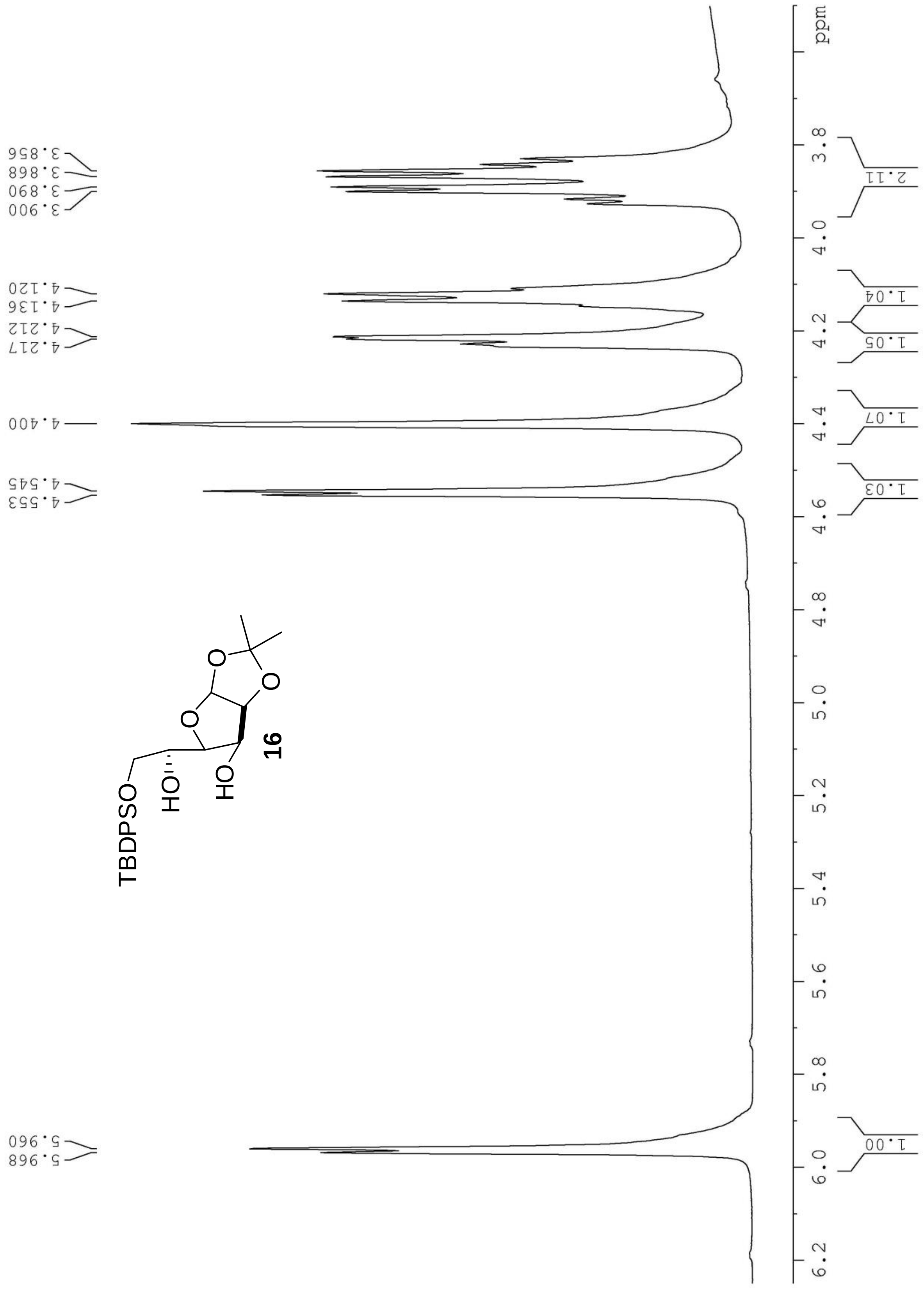
3.856
3.868
3.890
3.900
4.120
4.136
4.212
4.217
4.400
4.545
4.553
5.960
5.968
TBDPSO
HO
HO
O
O
O
16
2.11
1.04
1.05
1.07
1.03
1.00
3.8
4.0
4.2
4.4
4.6
4.8
5.0
5.2
5.4
5.6
5.8
6.0
6.2
ppm

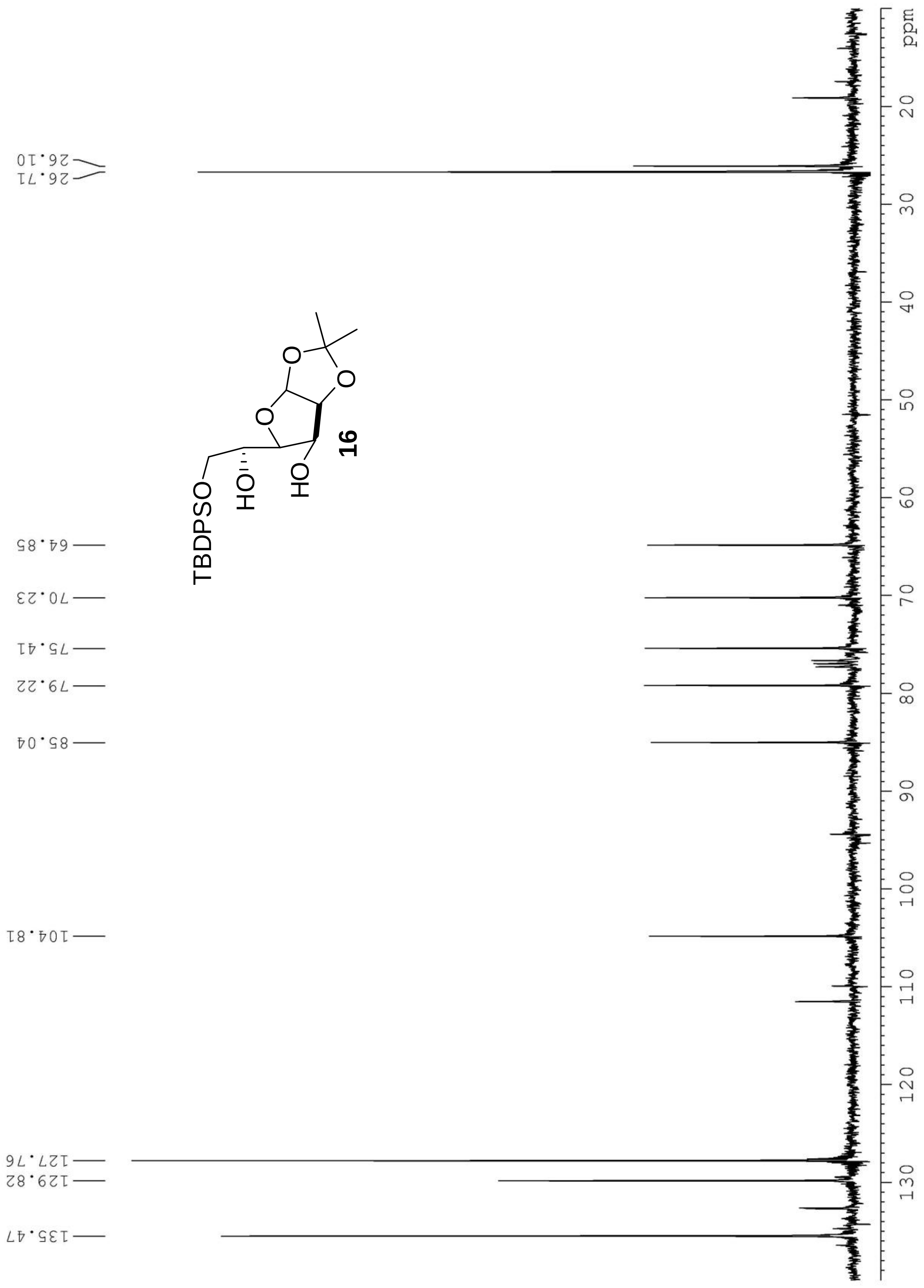
26.10
26.71
64.85
70.23
75.41
79.22
85.04
104.81
127.76
129.82
135.47
TBDPSO
HO
HO
O
O
O
16
ppm
20
30
40
50
60
70
80
90
100
110
120
130

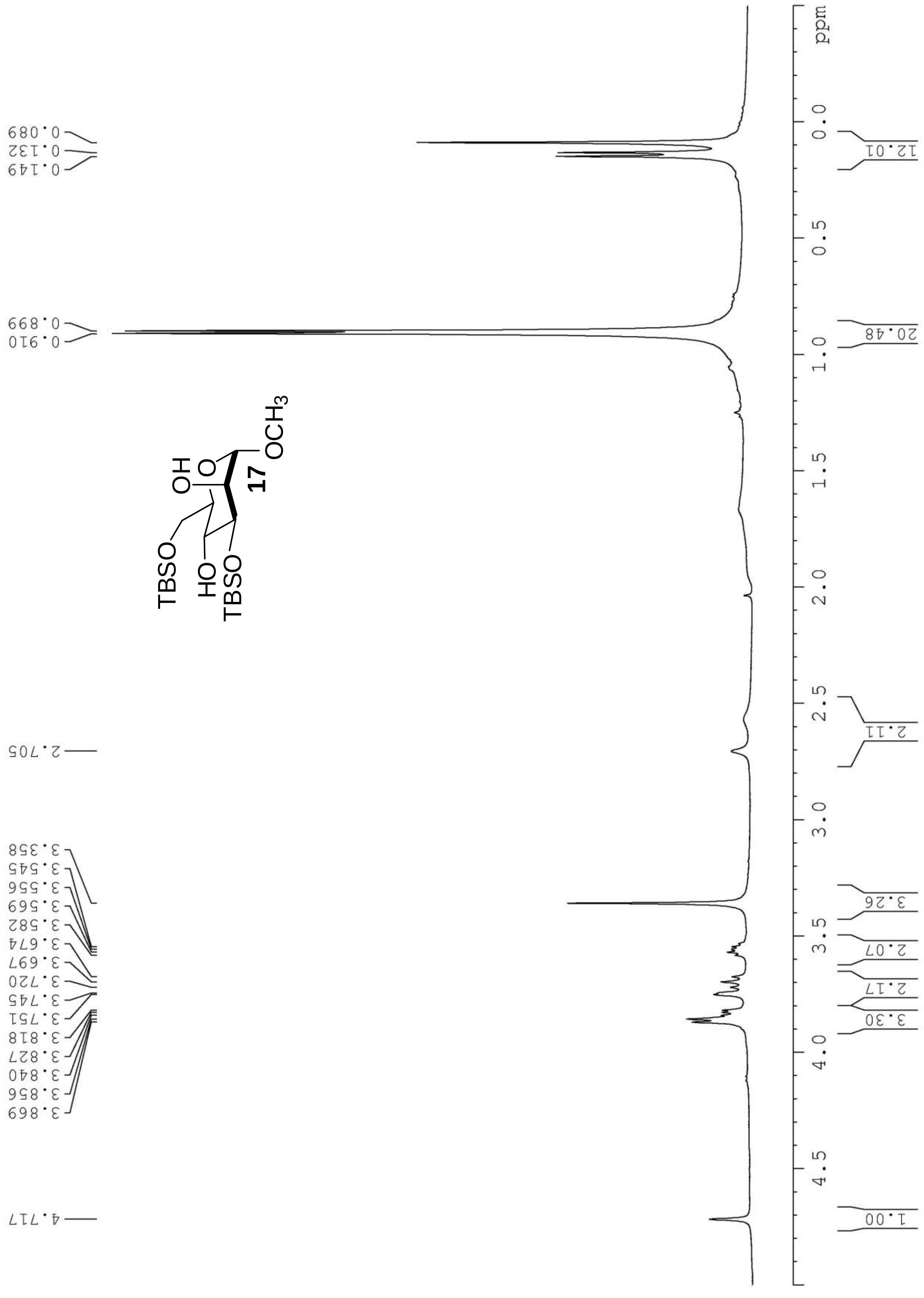

0.089
0.132
0.149
0.899
0.910
2.705
3.358
3.545
3.556
3.569
3.582
3.674
3.697
3.720
3.745
3.751
3.818
3.827
3.840
3.856
3.869
4.717
TBSO
HO
TBSO
OH
O
OCH3
17
12.01
20.48
2.11
3.26
2.07
2.17
3.30
1.00
0.0
0.5
1.0
1.5
2.0
2.5
3.0
3.5
4.0
4.5
ppm

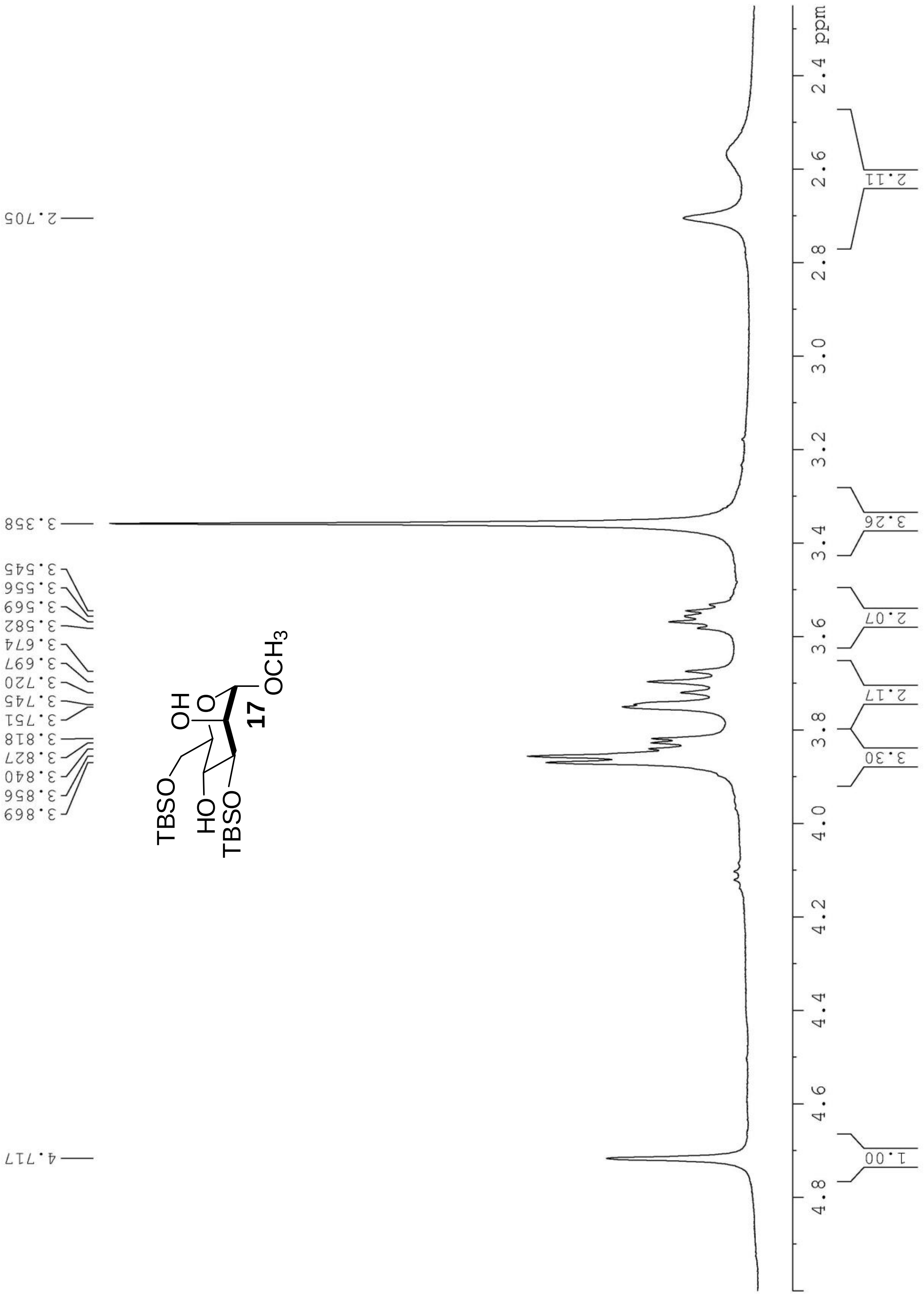
2.705
3.358
3.545
3.556
3.569
3.582
3.674
3.697
3.720
3.745
3.751
3.818
3.827
3.840
3.856
3.869
4.717
TBSO
HO
TBSO
OH
O
OCH3
17
ppm
2.11
3.26
2.07
2.17
3.30
1.00

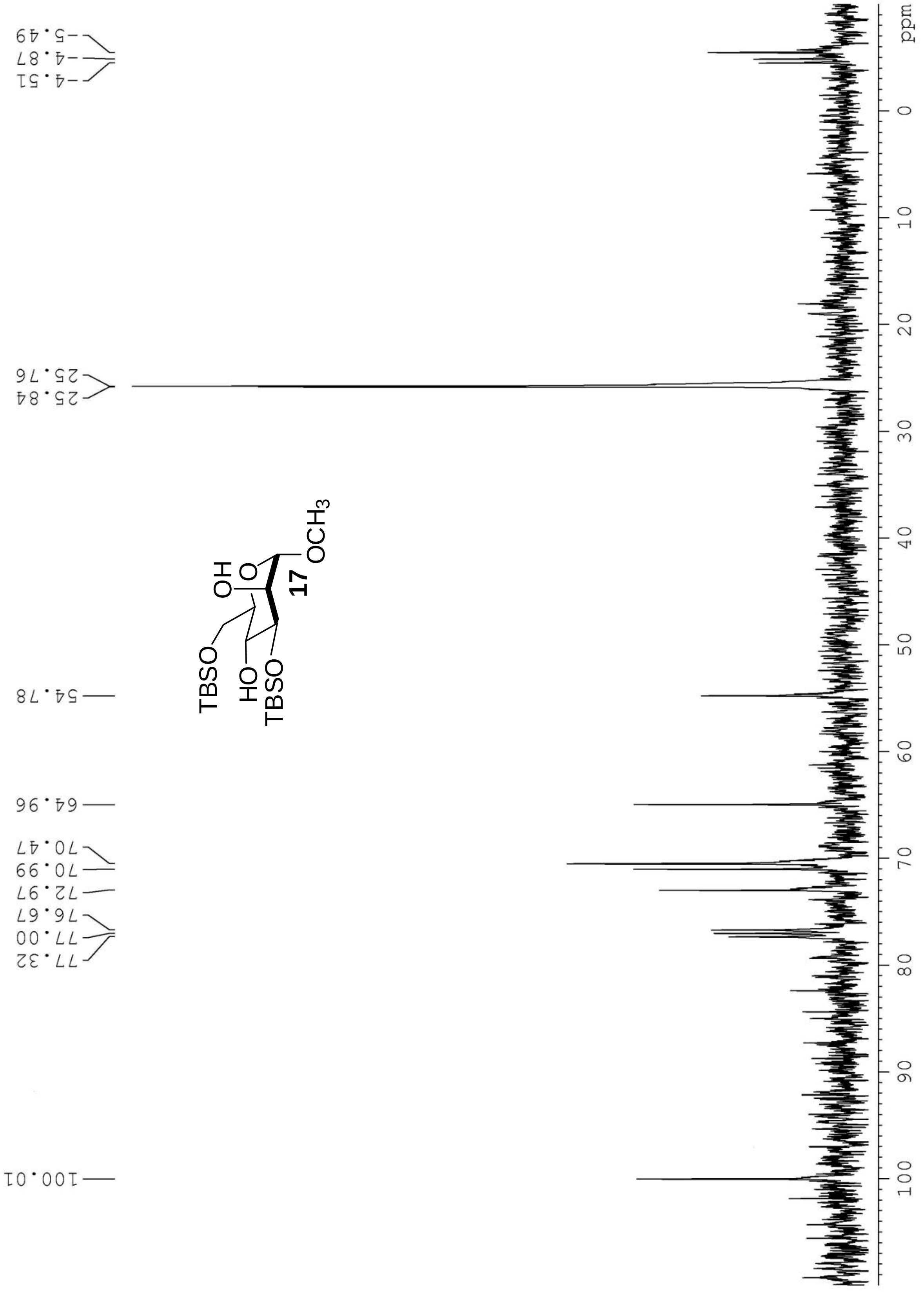
100.01
77.32
77.00
76.67
72.97
70.99
70.47
64.96
54.78
25.84
25.76
-4.51
-4.87
-5.49
TBSO
HO
TBSO
OH
O
OCH3
17
ppm

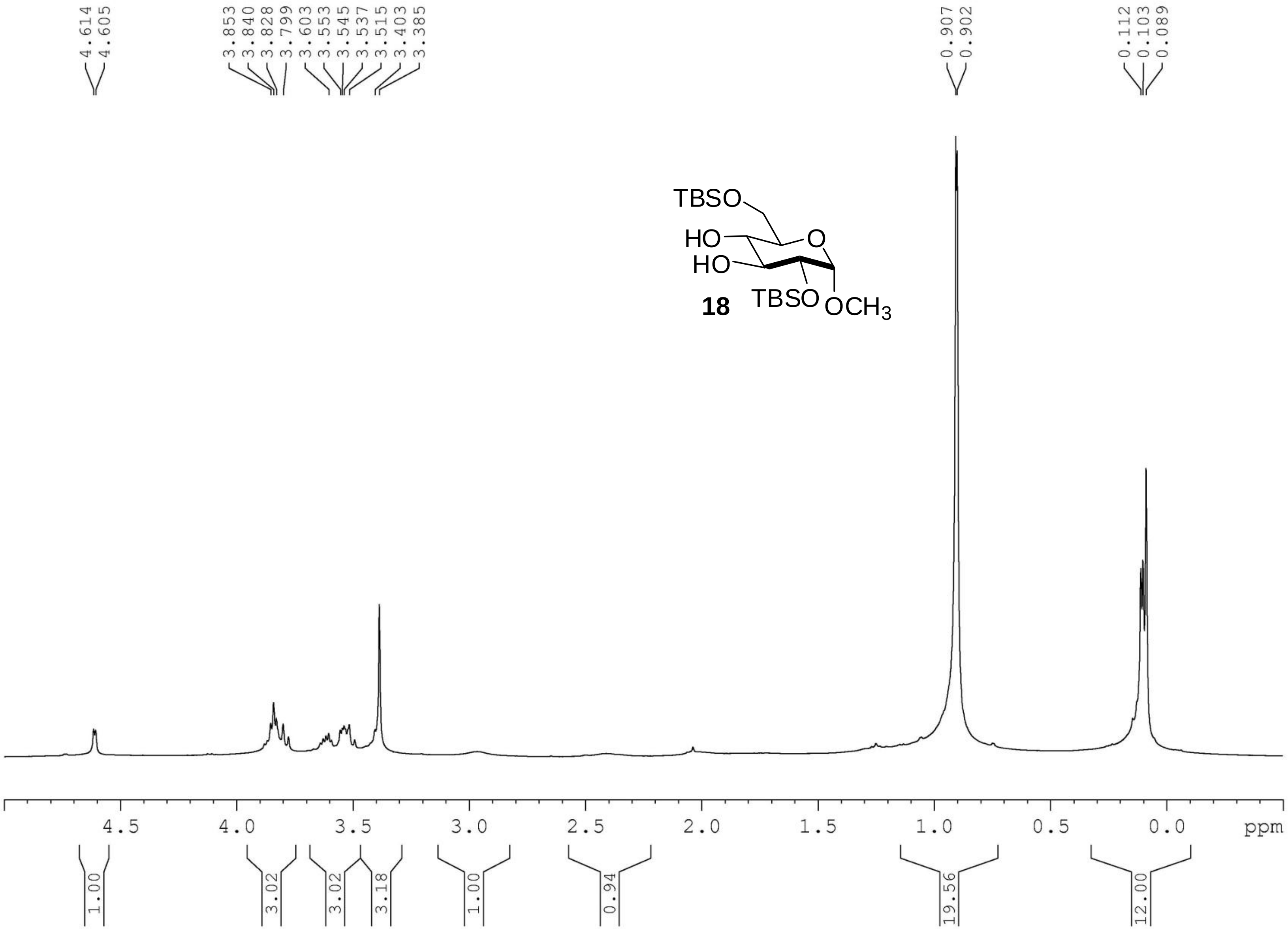
4.614
4.605
3.853
3.840
3.828
3.799
3.603
3.553
3.545
3.537
3.515
3.403
3.385
0.907
0.902
0.112
0.103
0.089
TBSO
HO
HO
O
TBSO
OCH3
18
4.5
4.0
3.5
3.0
2.5
2.0
1.5
1.0
0.5
0.0
ppm
1.00
3.02
3.02
3.18
1.00
0.94
19.56
12.00

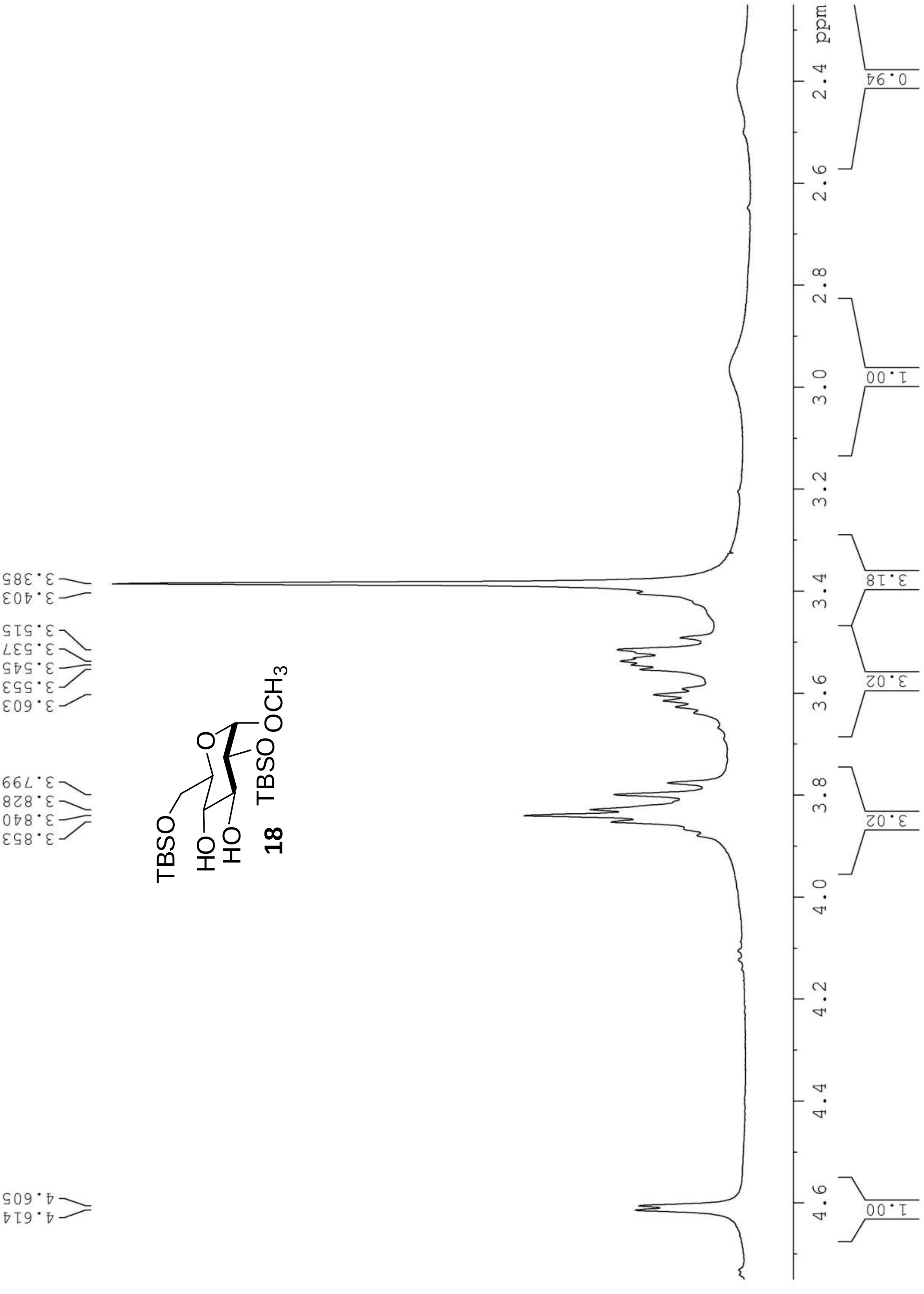
4.614
4.605
3.853
3.840
3.828
3.799
3.603
3.553
3.545
3.537
3.515
3.403
3.385
TBSO
HO
HO
TBSO
O
OCH3
18
ppm
1.00
3.02
3.02
3.18
1.00
0.94

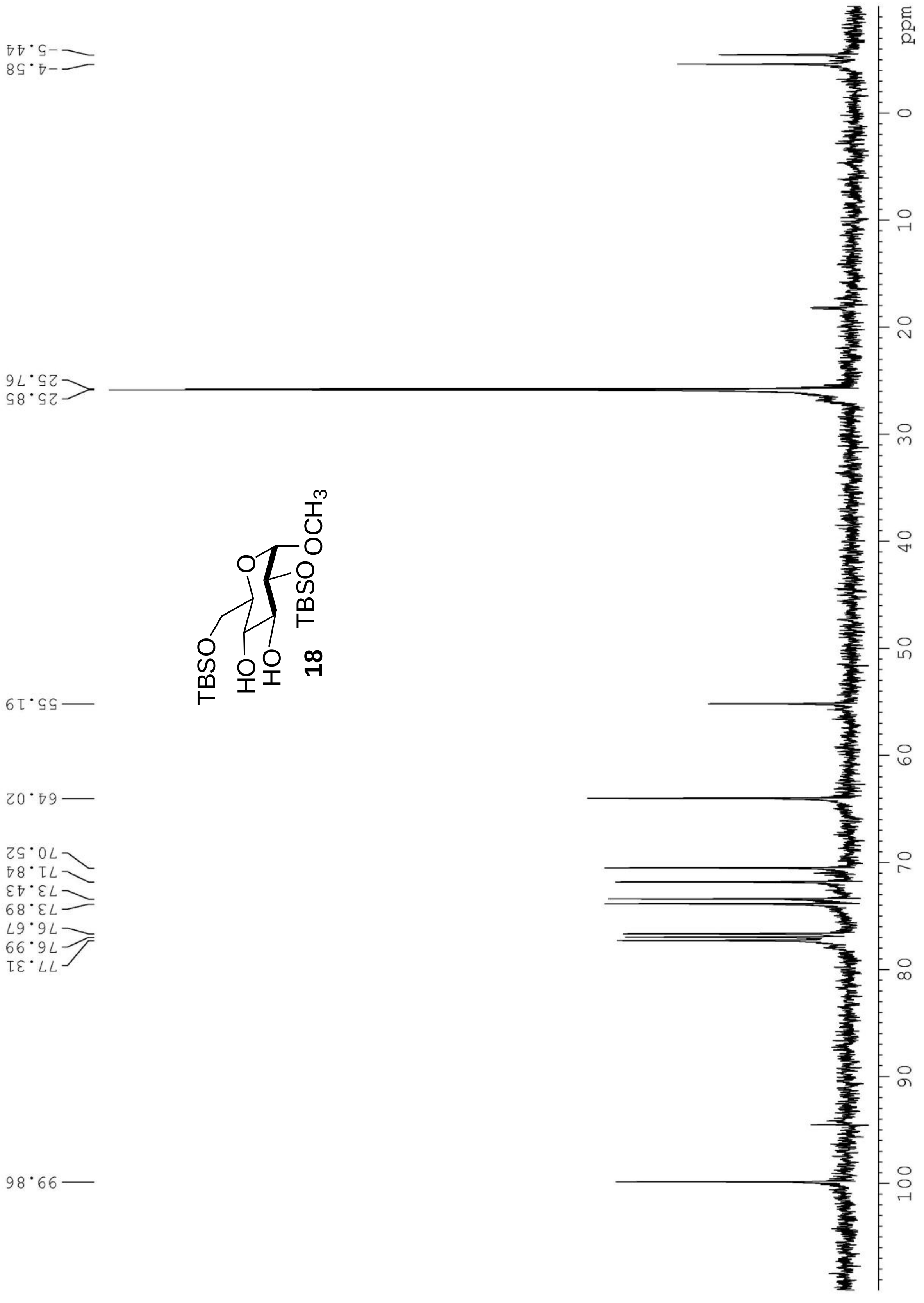

-5.44
-4.58
25.76
25.85
55.19
64.02
70.52
71.84
73.43
73.89
76.67
76.99
77.31
99.86
TBSO
HO
HO
O
TBSO
OCH3
18
ppm
0
10
20
30
40
50
60
70
80
90
100

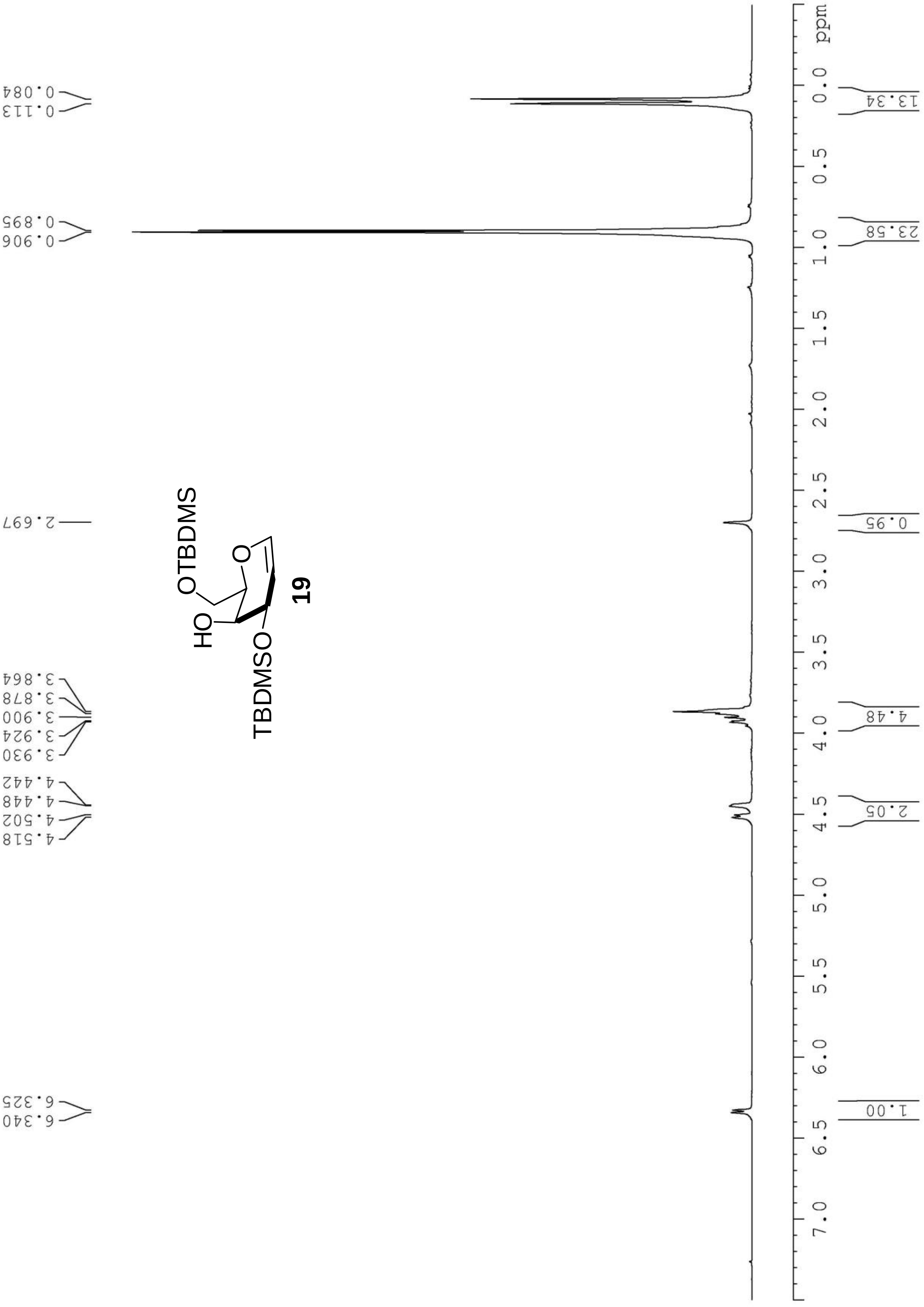
0.084
0.113
0.895
0.906
2.697
3.864
3.878
3.900
3.924
3.930
4.442
4.448
4.502
4.518
6.325
6.340
OTBDMS
HO
TBDMSO
O
19
ppm
13.34
23.58
0.95
4.48
2.05
1.00

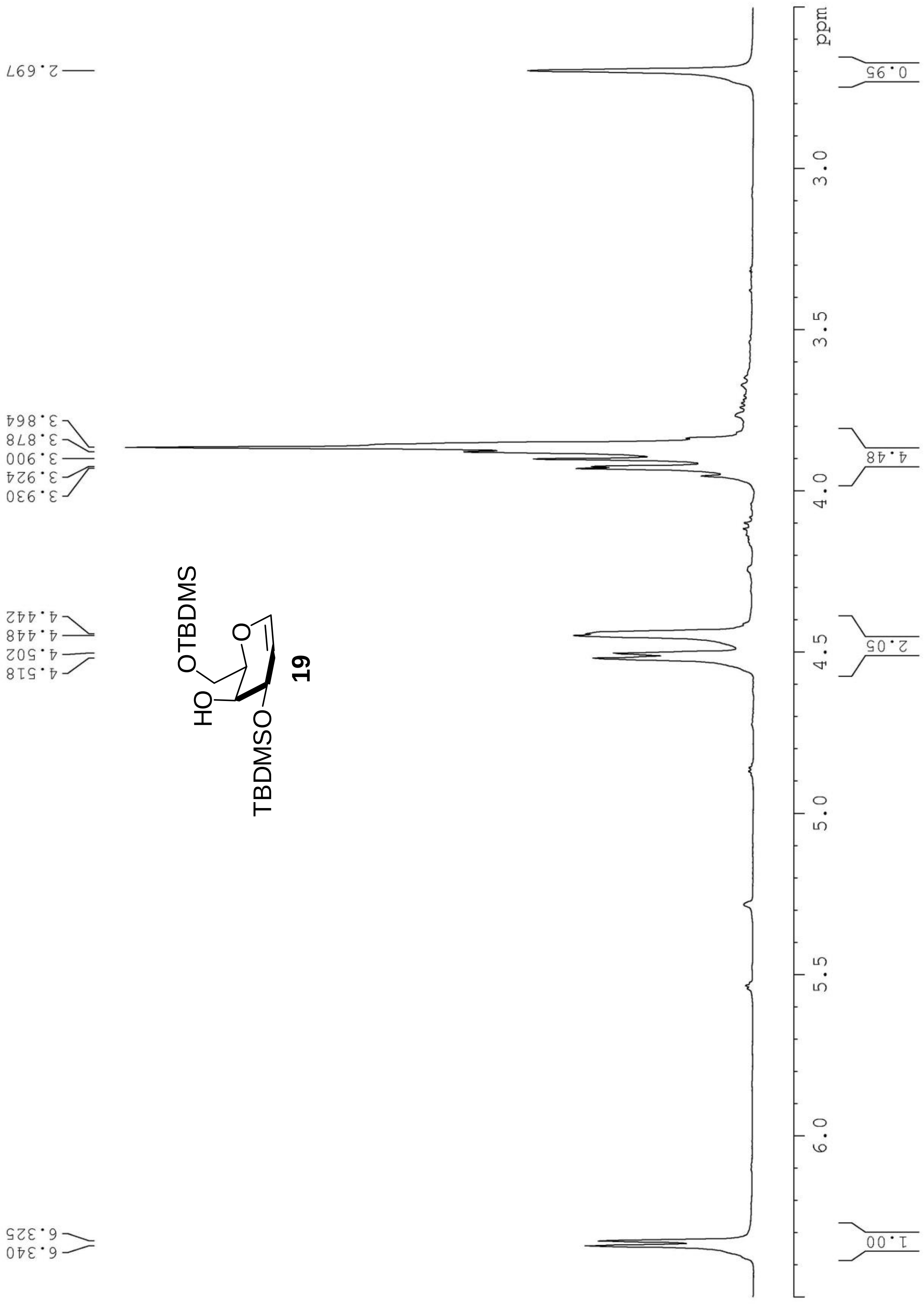
2.697
3.864
3.878
3.900
3.924
3.930
4.442
4.448
4.502
4.518
6.325
6.340
OTBDMS
HO
O
TBDMSO
ppm
3.0
3.5
4.0
4.5
5.0
5.5
6.0
0.95
4.48
2.05
1.00

19

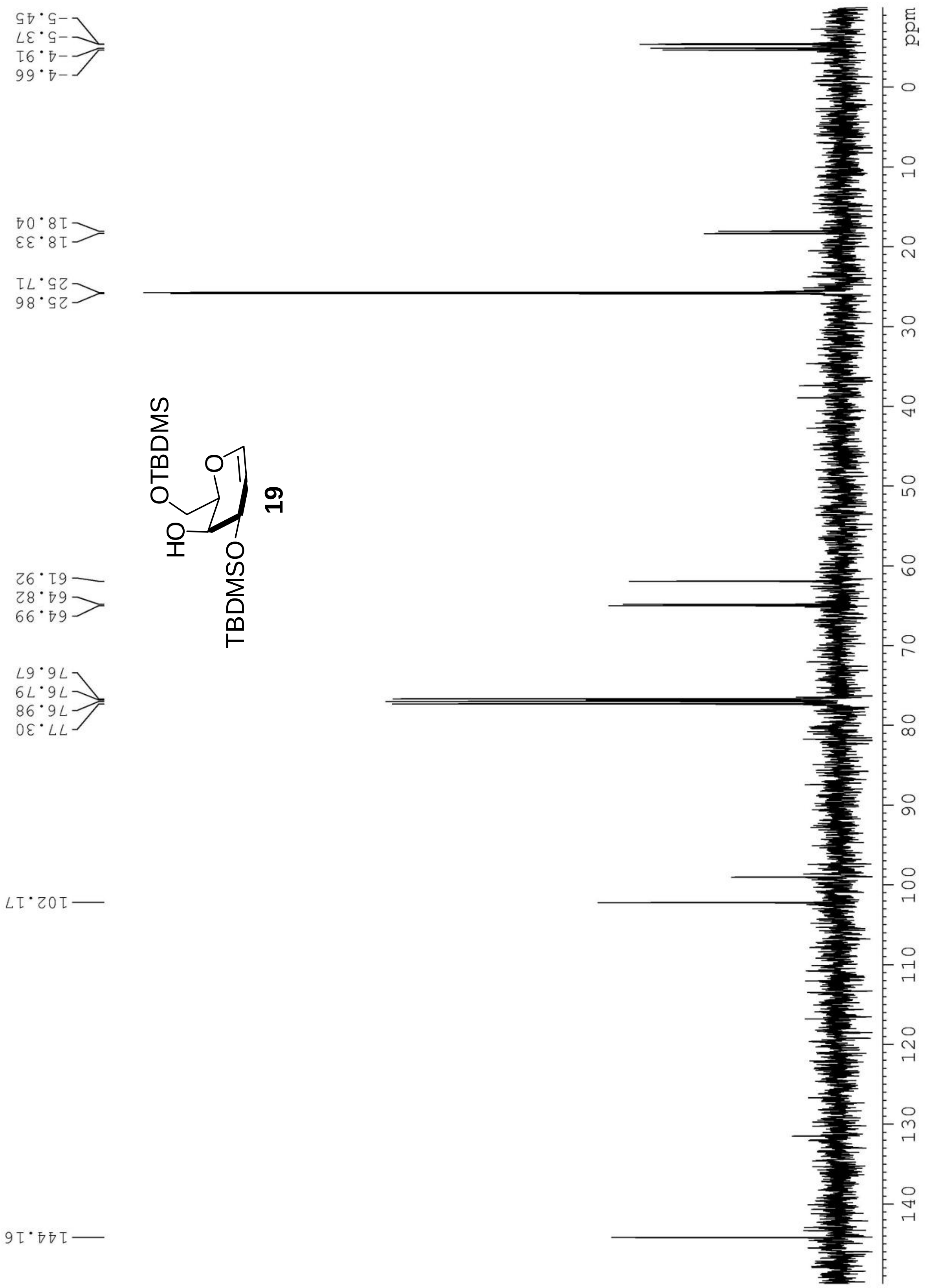

-5.45
-5.37
-4.91
-4.66
18.04
18.33
25.71
25.86
61.92
64.82
64.99
76.67
76.79
76.98
77.30
102.17
144.16
OTBDMS
O
HO
TBDMSO
19
ppm

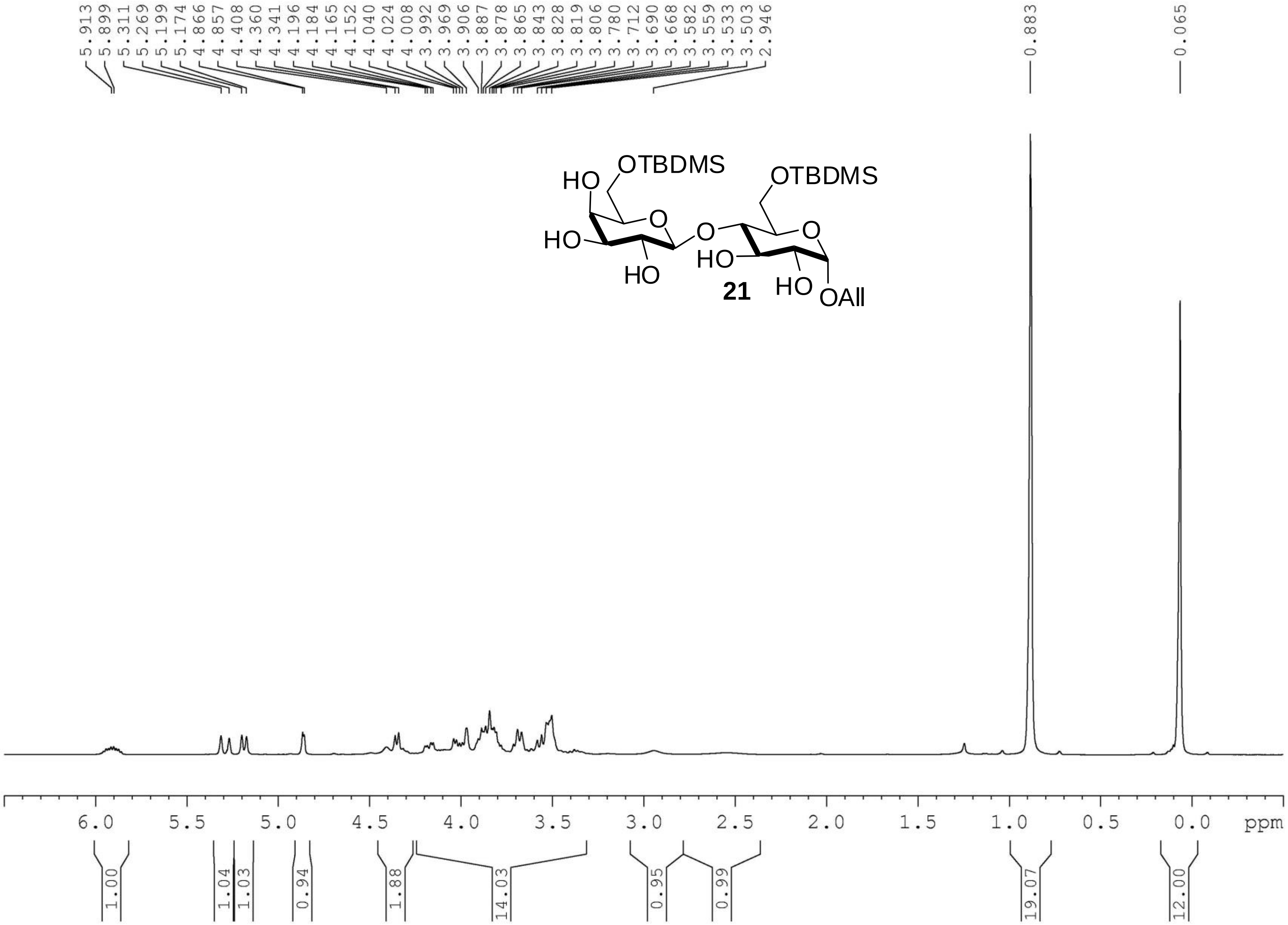
5.913
5.899
5.311
5.269
5.199
5.174
4.866
4.857
4.408
4.360
4.341
4.196
4.184
4.165
4.152
4.040
4.024
4.008
3.992
3.969
3.906
3.887
3.878
3.865
3.843
3.828
3.819
3.806
3.780
3.712
3.690
3.668
3.582
3.559
3.533
3.503
2.946
0.883
0.065
OTBDMS
HO
HO
HO
O
O
OTBDMS
O
HO
HO
OAll
21
6.0
5.5
5.0
4.5
4.0
3.5
3.0
2.5
2.0
1.5
1.0
0.5
0.0
ppm
1.00
1.04
1.03
0.94
1.88
14.03
0.95
0.99
19.07
12.00

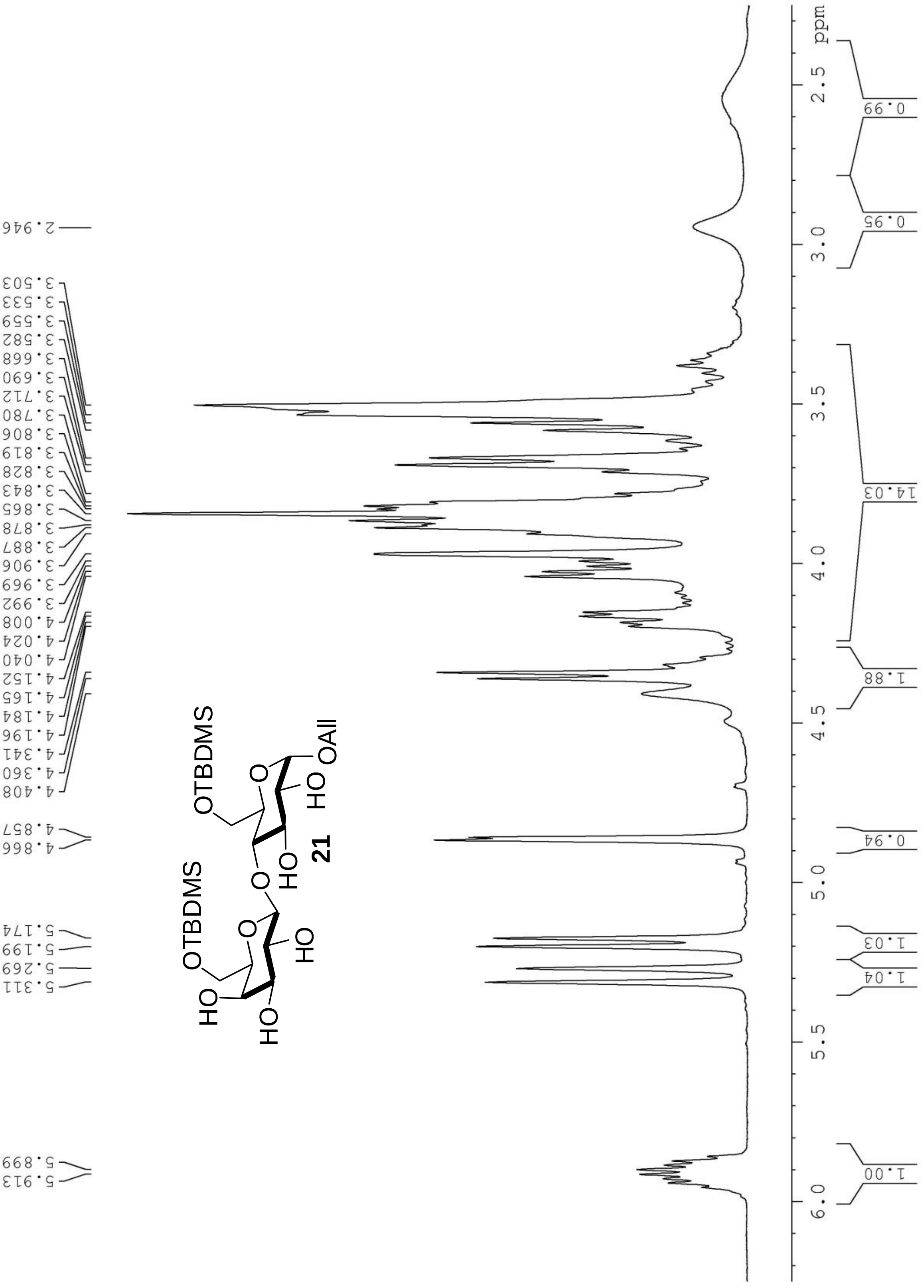
OTBDMS
OTBDMS
OAll
21
ppm

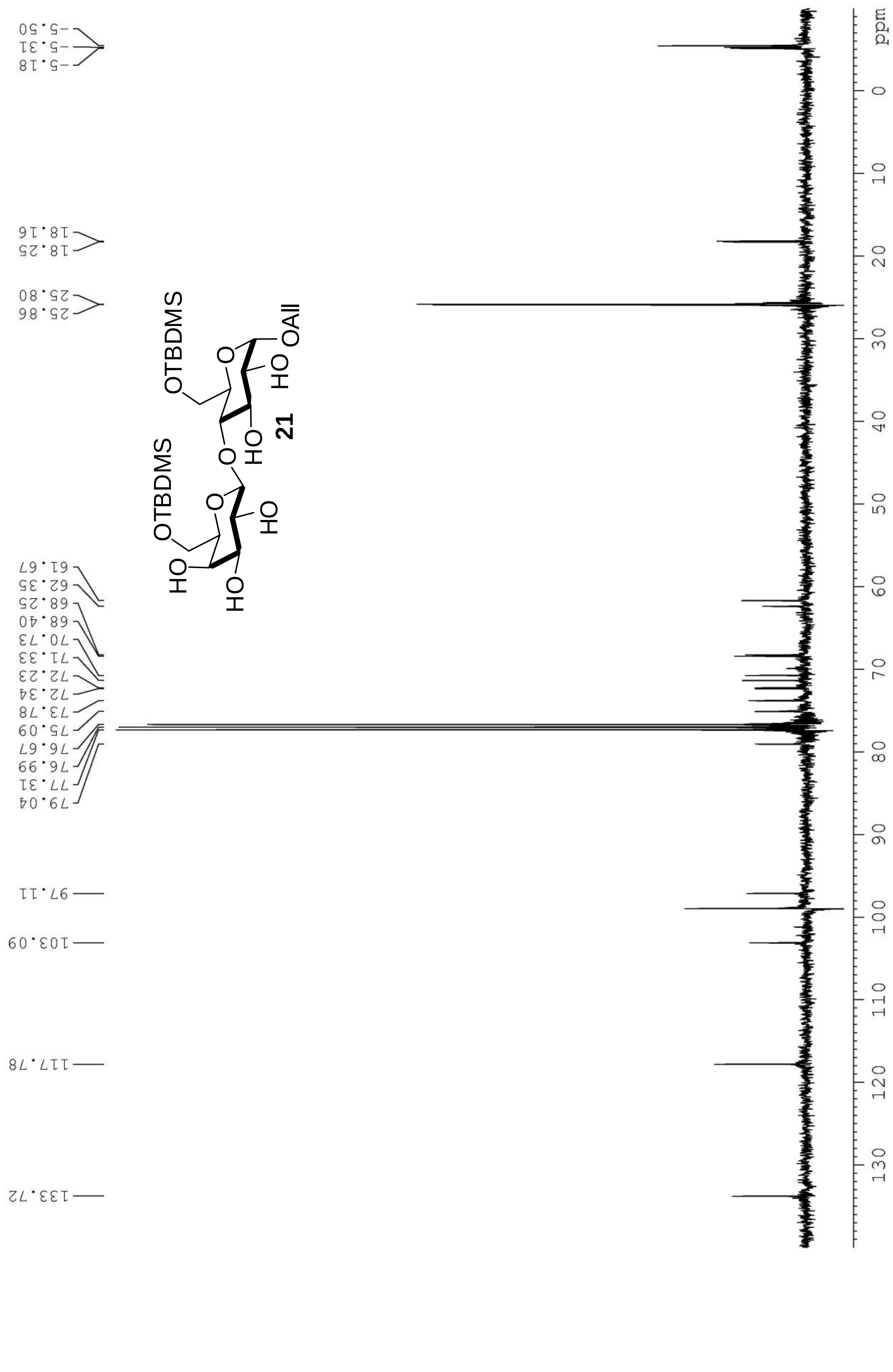
-5.50
-5.31
-5.18
18.16
18.25
25.80
25.86
61.67
62.35
68.25
68.40
70.73
71.33
72.23
72.34
73.78
75.09
76.67
76.99
77.31
79.04
97.11
103.09
117.78
133.72
OTBDMS
OTBDMS
OAll
HO
HO
HO
HO
HO
O
O
O
21
ppm
0
10
20
30
40
50
60
70
80
90
100
110
120
130

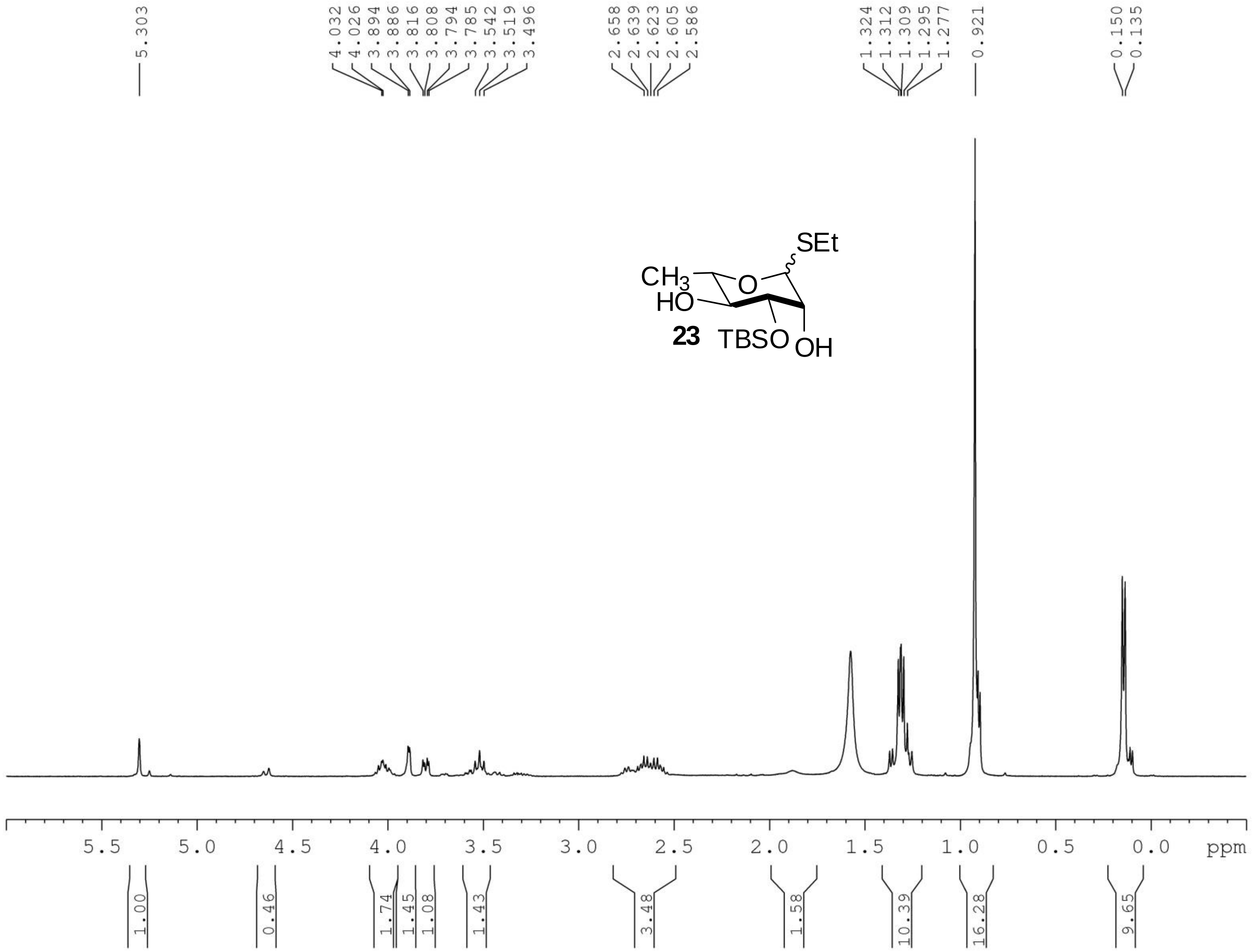
5.303
4.032
4.026
3.894
3.886
3.816
3.808
3.794
3.785
3.542
3.519
3.496
2.658
2.639
2.623
2.605
2.586
1.324
1.312
1.309
1.295
1.277
0.921
0.150
0.135
SEt
CH3
O
HO
23
TBSO
OH
5.5
5.0
4.5
4.0
3.5
3.0
2.5
2.0
1.5
1.0
0.5
0.0
ppm
1.00
0.46
1.74
1.45
1.08
1.43
3.48
1.58
10.39
16.28
9.65

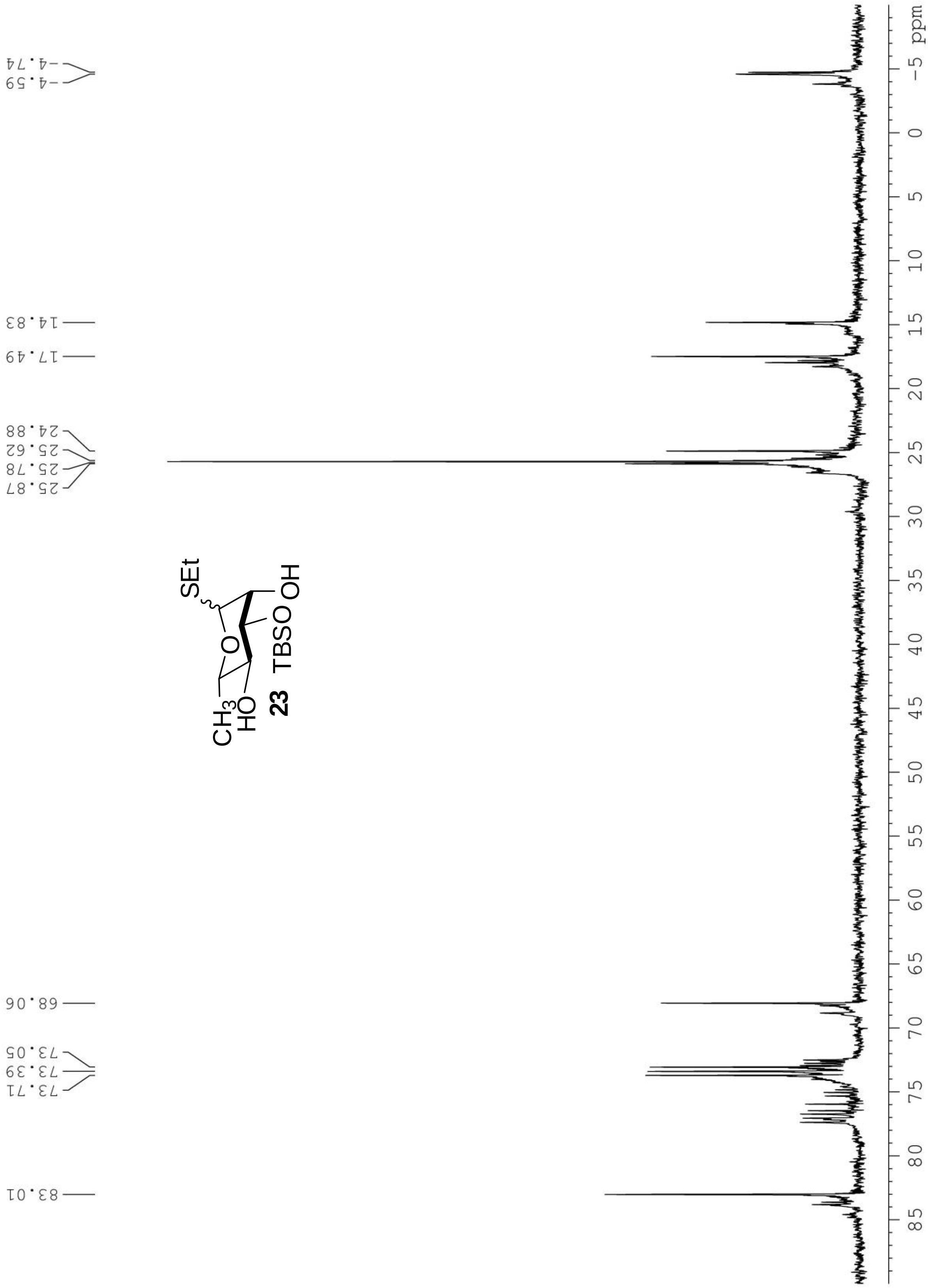

-4.74
-4.59
14.83
17.49
24.88
25.62
25.78
25.87
68.06
73.05
73.39
73.71
83.01
SEt
O
TBSO
OH
HO
CH_3
23
ppm

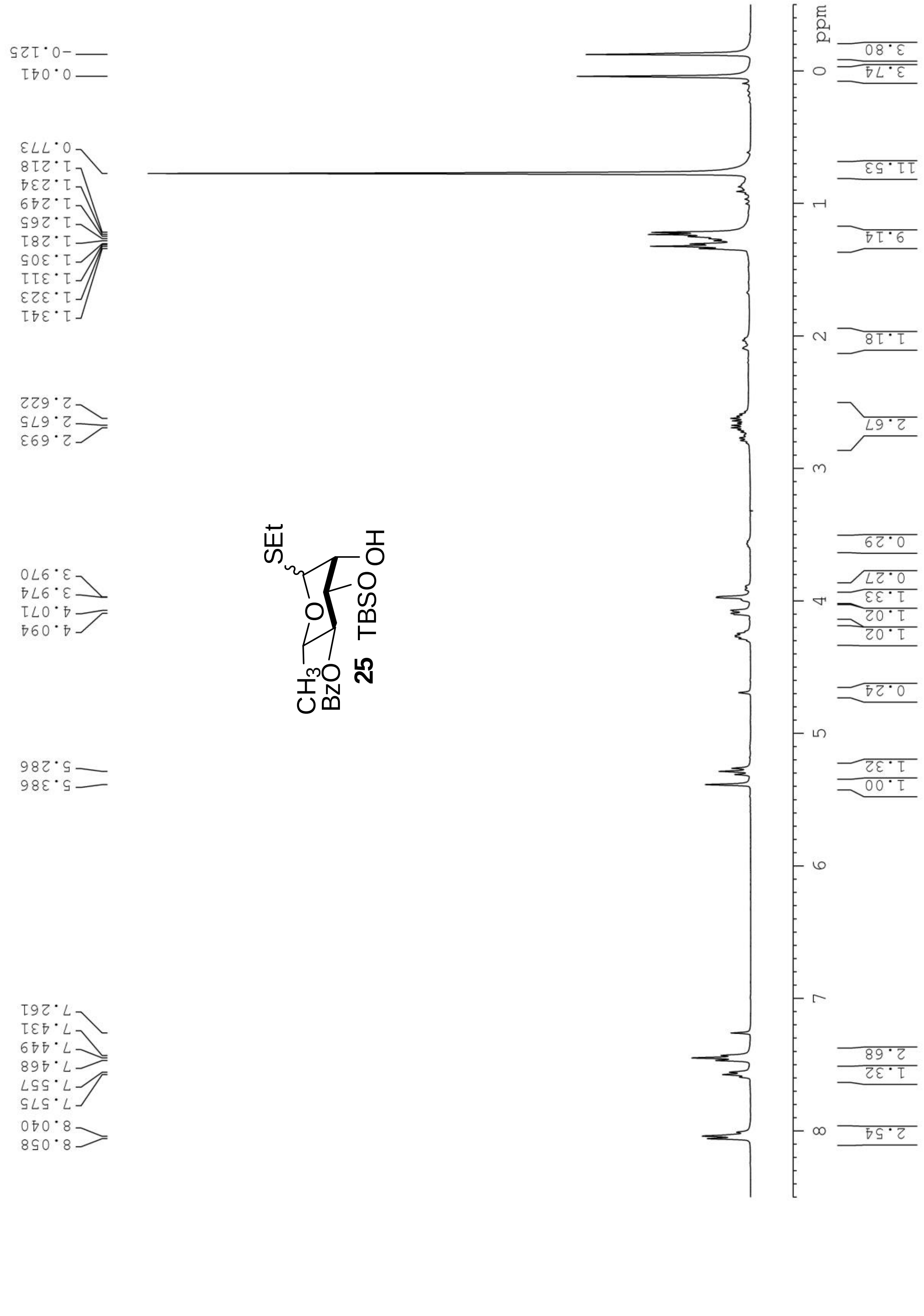
-0.125
0.041
0.773
1.218
1.234
1.249
1.265
1.281
1.305
1.311
1.323
1.341
2.622
2.675
2.693
3.970
3.974
4.071
4.094
5.286
5.386
7.261
7.431
7.449
7.468
7.557
7.575
8.040
8.058
SEt
O
CH3
BzO
TBSO
OH
25
ppm
0
1
2
3
4
5
6
7
8
3.80
3.74
11.53
9.14
1.18
2.67
0.29
0.27
1.33
1.02
1.02
0.24
1.32
1.00
2.68
1.32
2.54

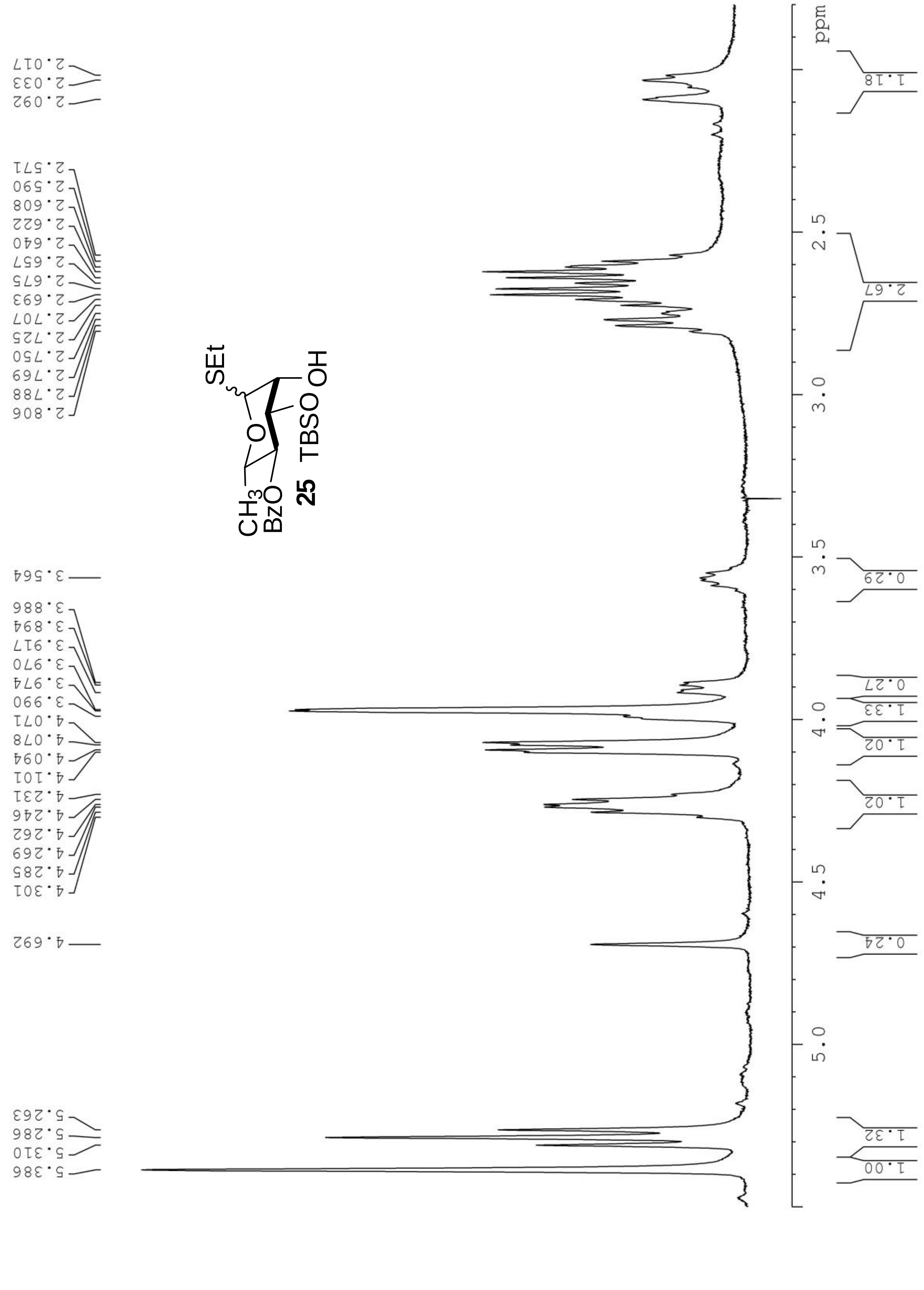

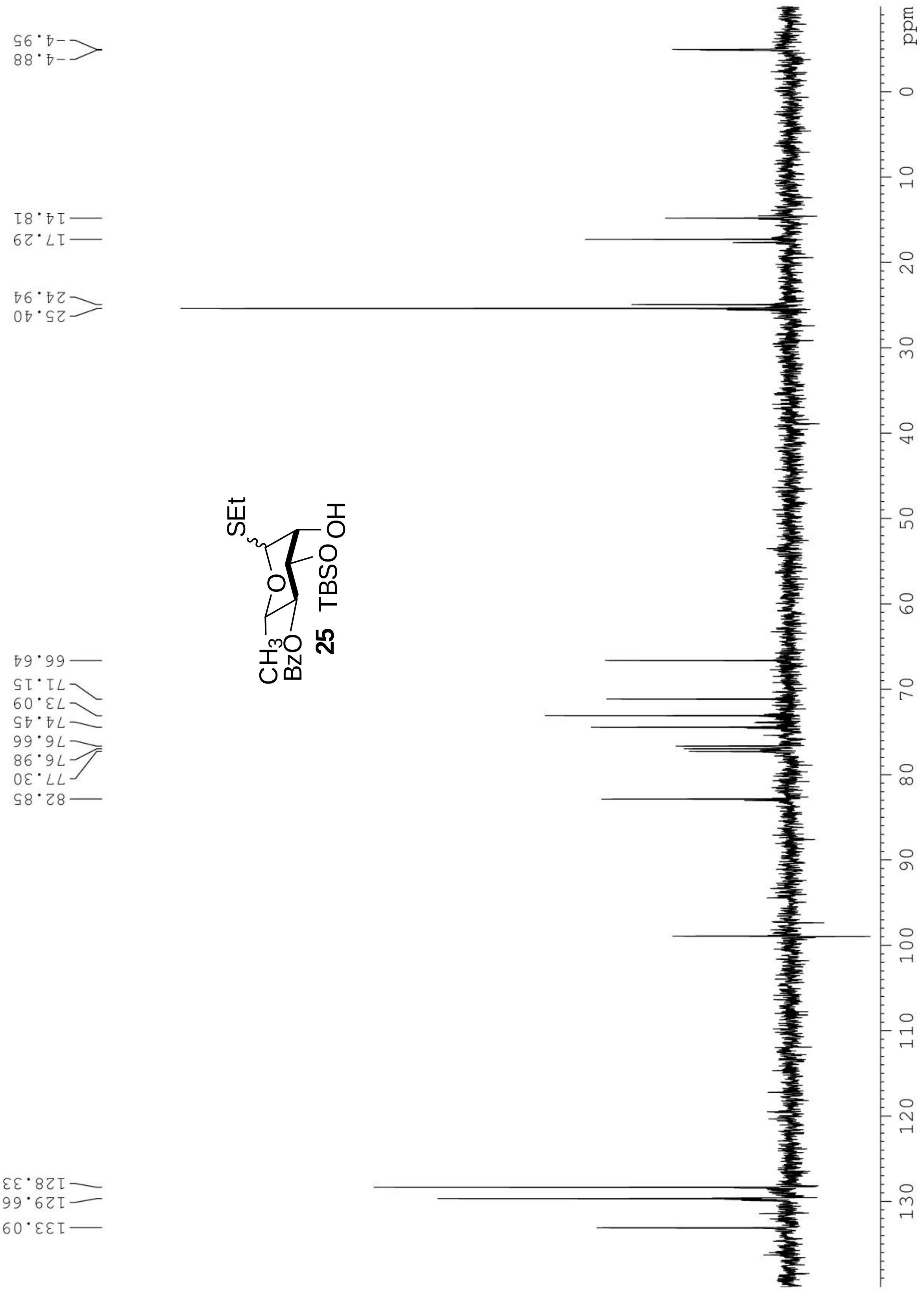
-4.95
-4.88
14.81
17.29
24.94
25.40
66.64
71.15
73.09
74.45
76.66
76.98
77.30
82.85
128.33
129.66
133.09
SEt
OH
TBSO
O
CH3
BzO
25
ppm

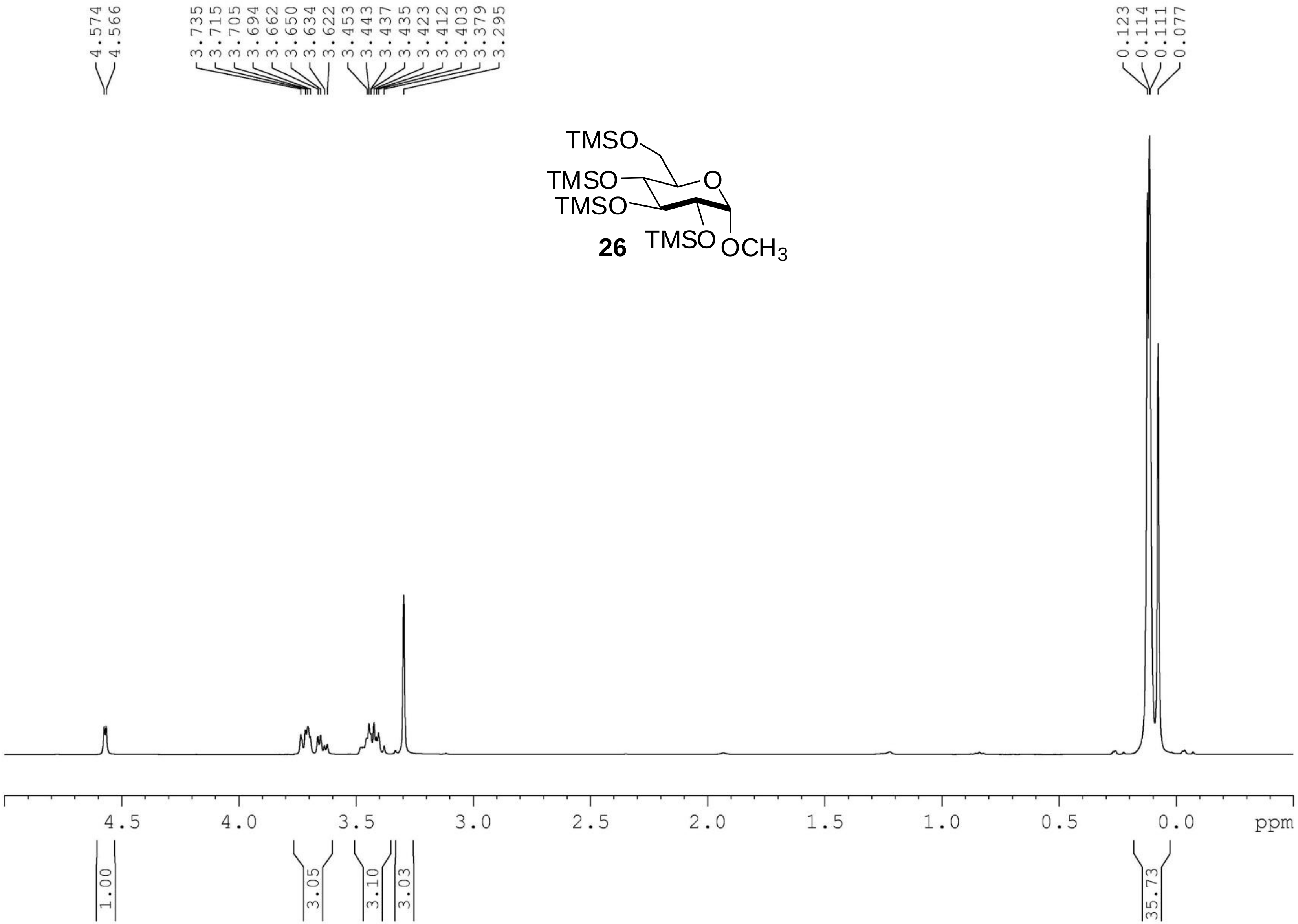
4.574
4.566
3.735
3.715
3.705
3.694
3.662
3.650
3.634
3.622
3.453
3.443
3.437
3.435
3.423
3.412
3.403
3.379
3.295
0.123
0.114
0.111
0.077
TMSO
TMSO
TMSO
O
26
TMSO
OCH3
4.5
4.0
3.5
3.0
2.5
2.0
1.5
1.0
0.5
0.0
ppm
1.00
3.05
3.10
3.03
35.73

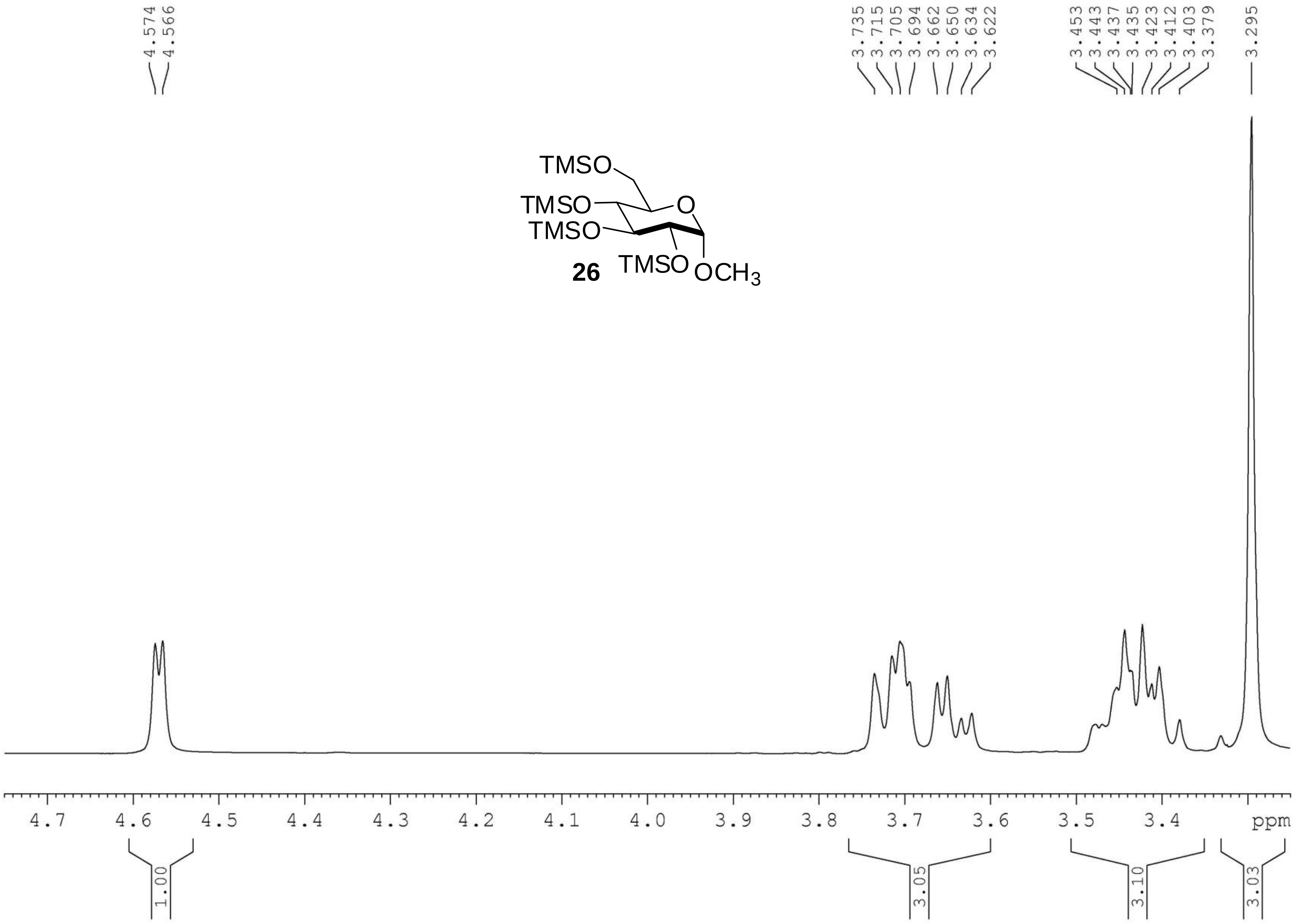
4.574
4.566
3.735
3.715
3.705
3.694
3.662
3.650
3.634
3.622
3.453
3.443
3.437
3.435
3.423
3.412
3.403
3.379
3.295
TMSO
TMSO
TMSO
O
TMSO
OCH3
26
4.7
4.6
4.5
4.4
4.3
4.2
4.1
4.0
3.9
3.8
3.7
3.6
3.5
3.4
ppm
1.00
3.05
3.10
3.03

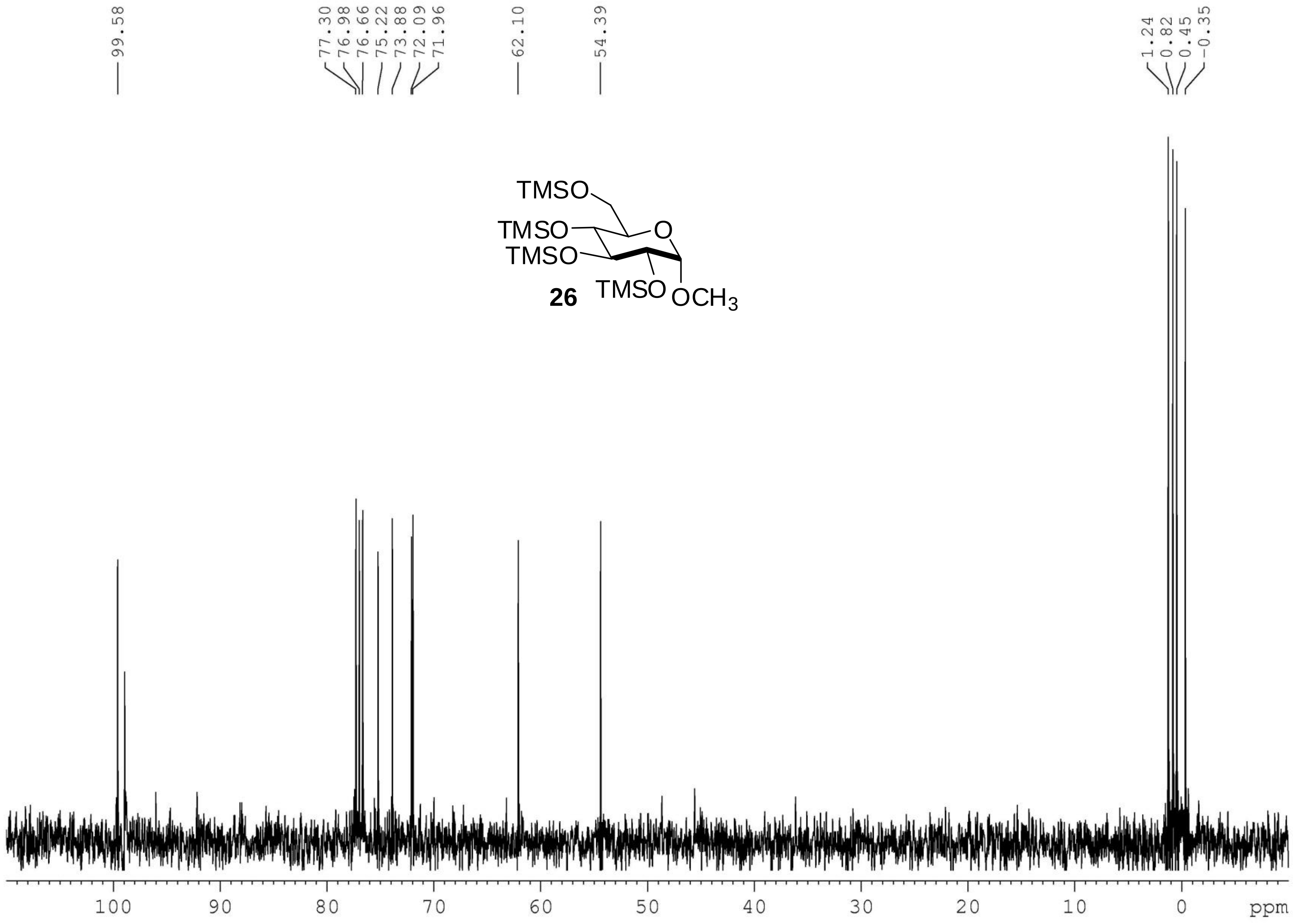
99.58
77.30
76.98
76.66
75.22
73.88
72.09
71.96
62.10
54.39
1.24
0.82
0.45
-0.35
TMSO
TMSO
TMSO
O
TMSO
OCH3
26
100
90
80
70
60
50
40
30
20
10
0
ppm

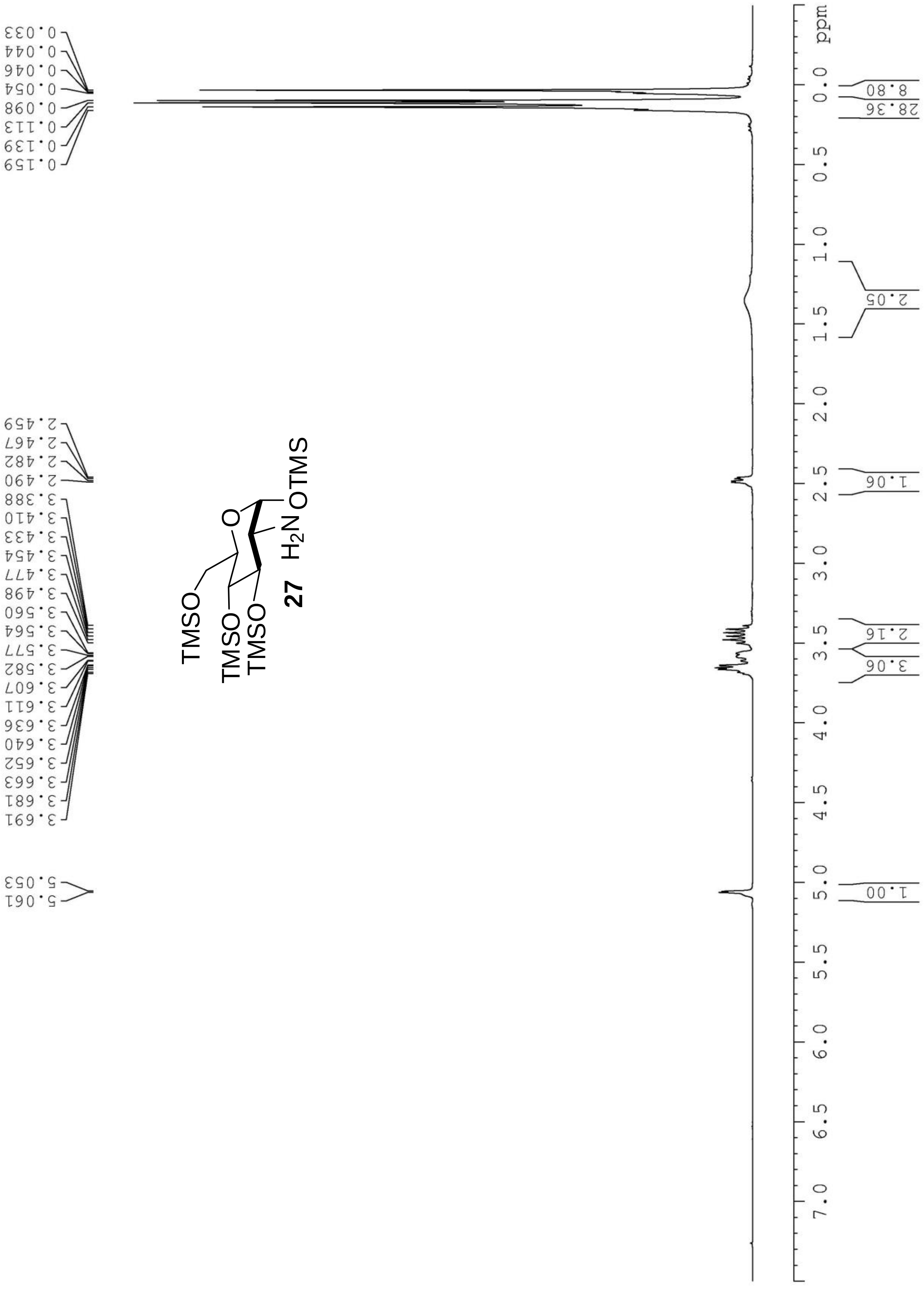
0.033
0.044
0.046
0.054
0.098
0.113
0.139
0.159
2.459
2.467
2.482
2.490
3.388
3.410
3.433
3.454
3.477
3.498
3.560
3.564
3.577
3.582
3.607
3.611
3.636
3.640
3.652
3.663
3.681
3.691
5.053
5.061
TMSO
TMSO
TMSO
O
H_2N
OTMS
ppm
0.0
0.5
1.0
1.5
2.0
2.5
3.0
3.5
4.0
4.5
5.0
5.5
6.0
6.5
7.0
8.80
28.36
2.05
1.06
2.16
3.06
1.00

27

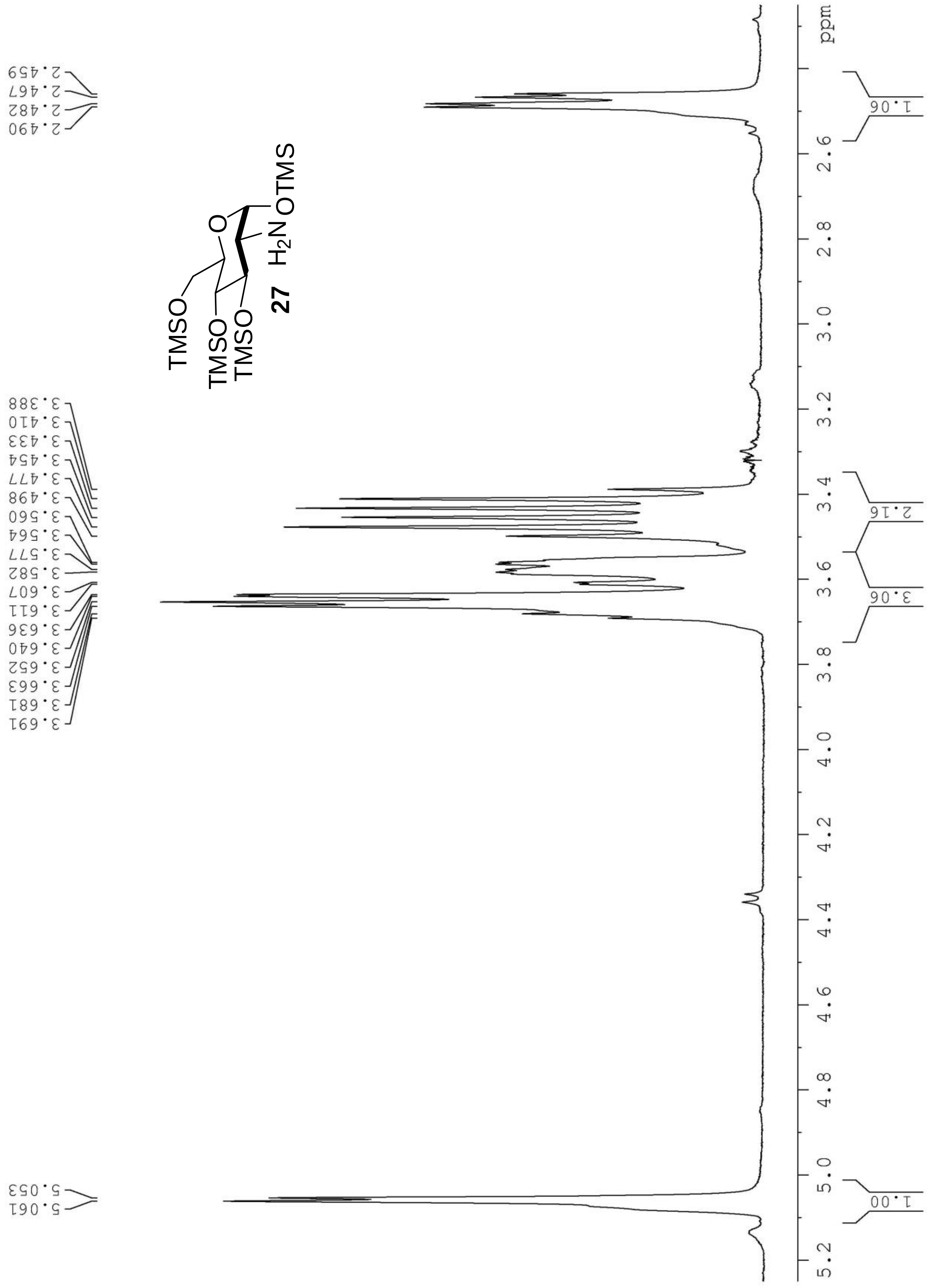
2.459
2.467
2.482
2.490
3.388
3.410
3.433
3.454
3.477
3.498
3.560
3.564
3.577
3.582
3.607
3.611
3.636
3.640
3.652
3.663
3.681
3.691
5.053
5.061
TMSO
TMSO
TMSO
O
H_2N
OTMS
27
1.06
2.16
3.06
1.00
2.6
2.8
3.0
3.2
3.4
3.6
3.8
4.0
4.2
4.4
4.6
4.8
5.0
5.2
ppm

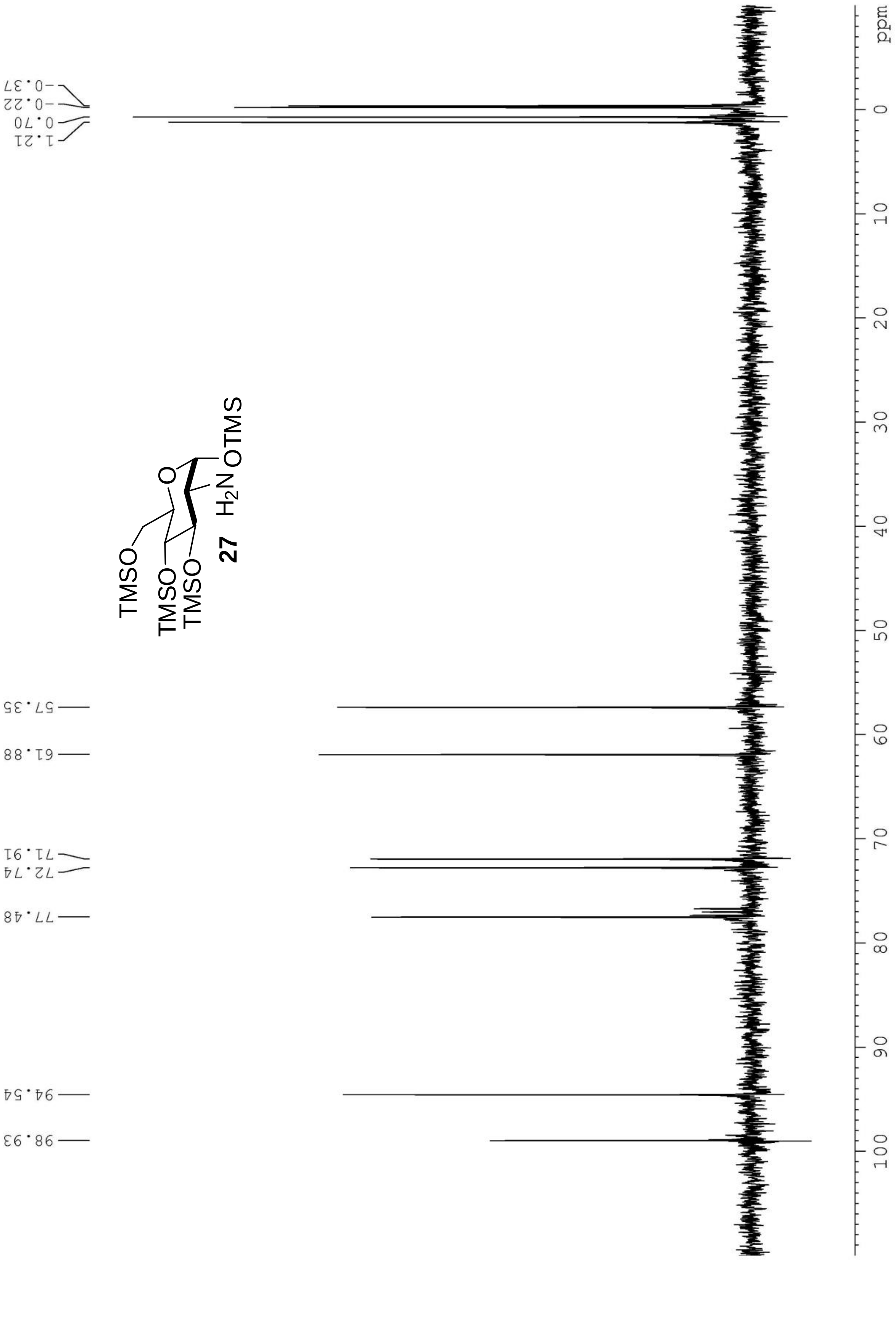
-0.37
-0.22
0.70
1.21
57.35
61.88
71.91
72.74
77.48
94.54
98.93
TMSO
TMSO
TMSO
O
H_2N
OTMS
27
ppm
0
10
20
30
40
50
60
70
80
90
100

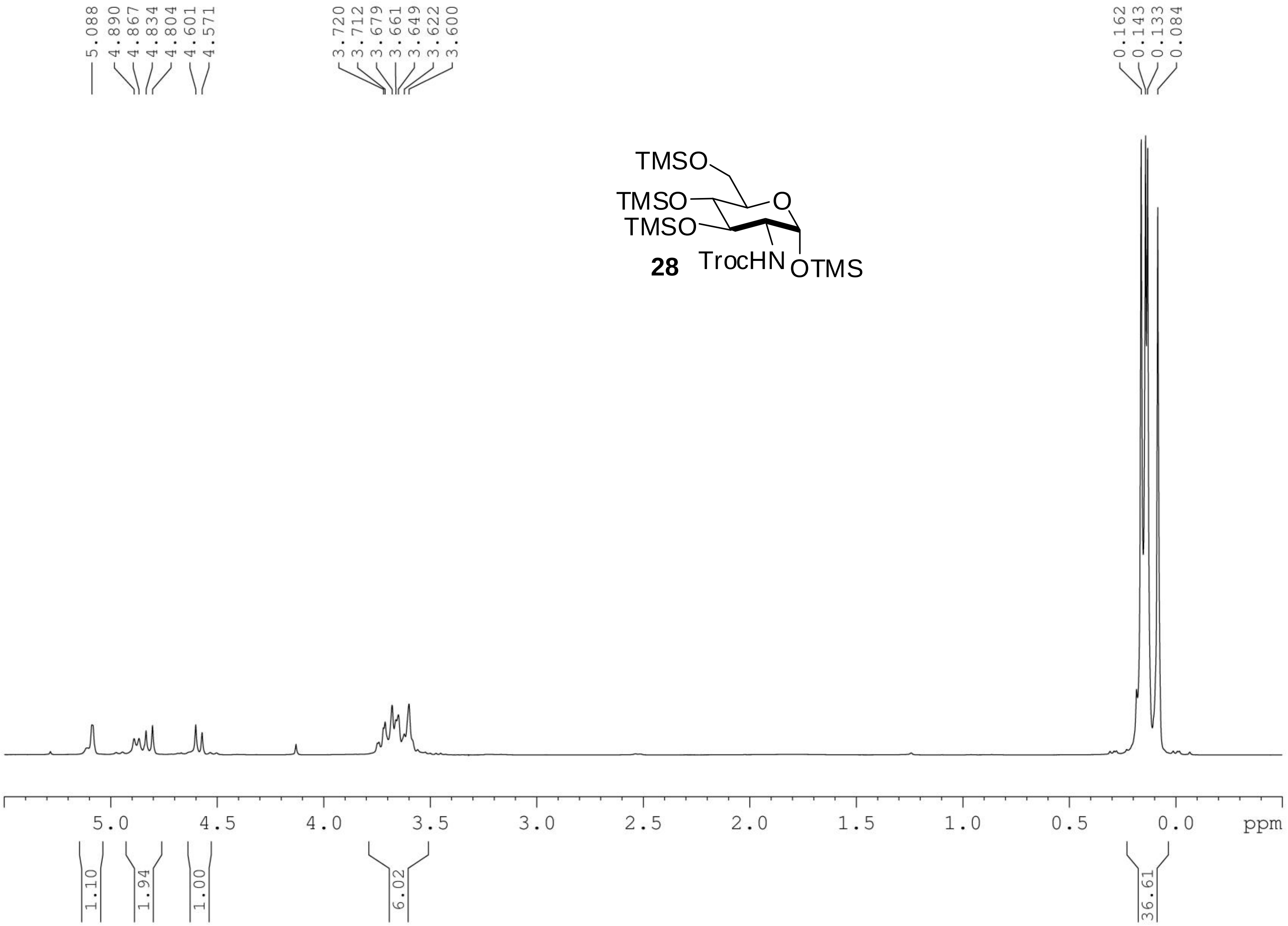
5.088
4.890
4.867
4.834
4.804
4.601
4.571
3.720
3.712
3.679
3.661
3.649
3.622
3.600
0.162
0.143
0.133
0.084
TMSO
TMSO
TMSO
O
TrocHN
OTMS
28
5.0
4.5
4.0
3.5
3.0
2.5
2.0
1.5
1.0
0.5
0.0
ppm
1.10
1.94
1.00
6.02
36.61

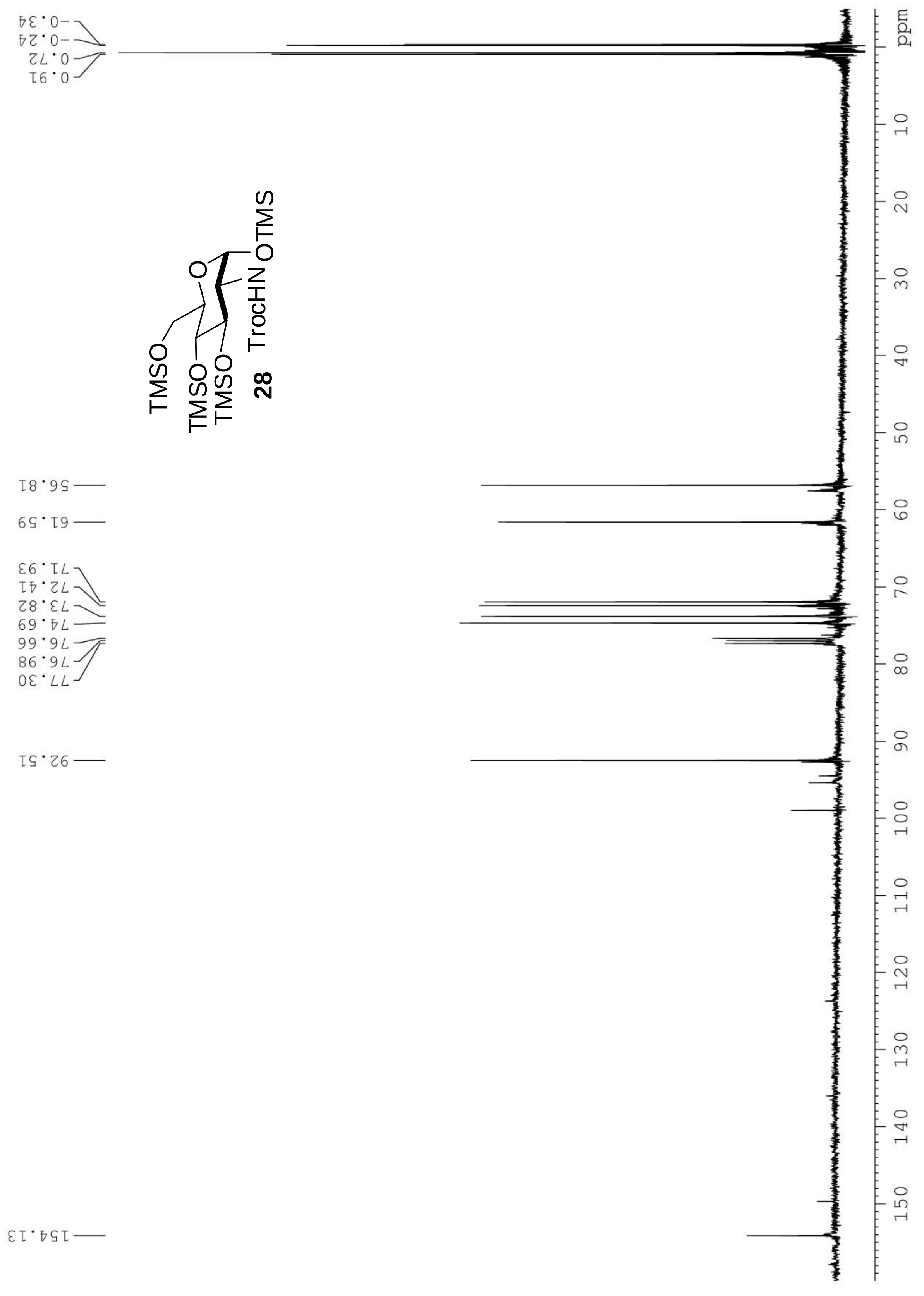
-0.34
-0.24
0.72
0.91
56.81
61.59
71.93
72.41
73.82
74.69
76.66
76.98
77.30
92.51
154.13
TMSO
TMSO
TMSO
O
TrocHN
OTMS
28
ppm
10
20
30
40
50
60
70
80
90
100
110
120
130
140
150

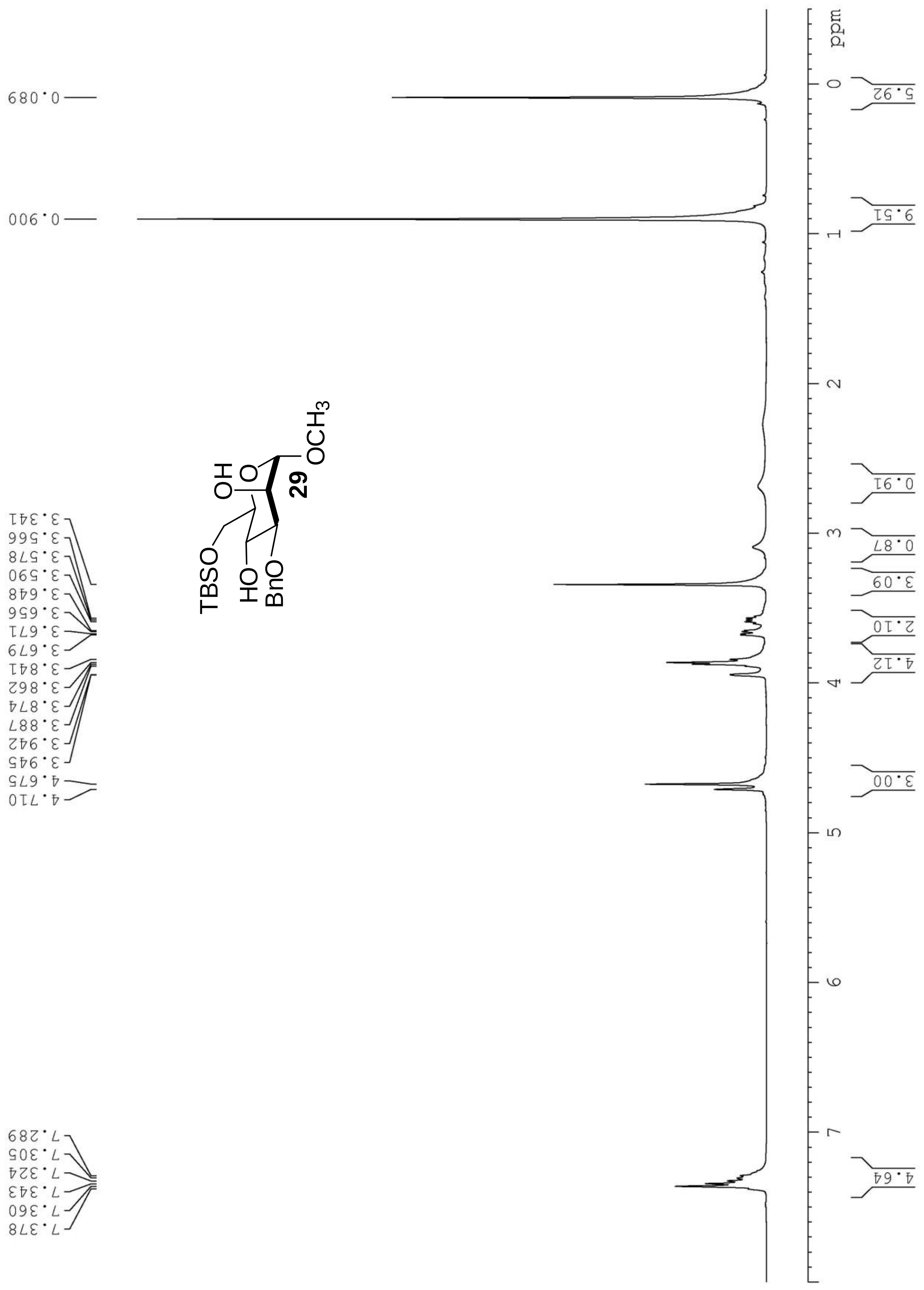

TBSO
HO
BnO
OH
O
OCH3
29
0.089
0.900
3.341
3.566
3.578
3.590
3.648
3.656
3.671
3.679
3.841
3.862
3.874
3.887
3.942
3.945
4.675
4.710
7.289
7.305
7.324
7.343
7.360
7.378
5.92
9.51
0.91
0.87
3.09
2.10
4.12
3.00
4.64
ppm
0
1
2
3
4
5
6
7

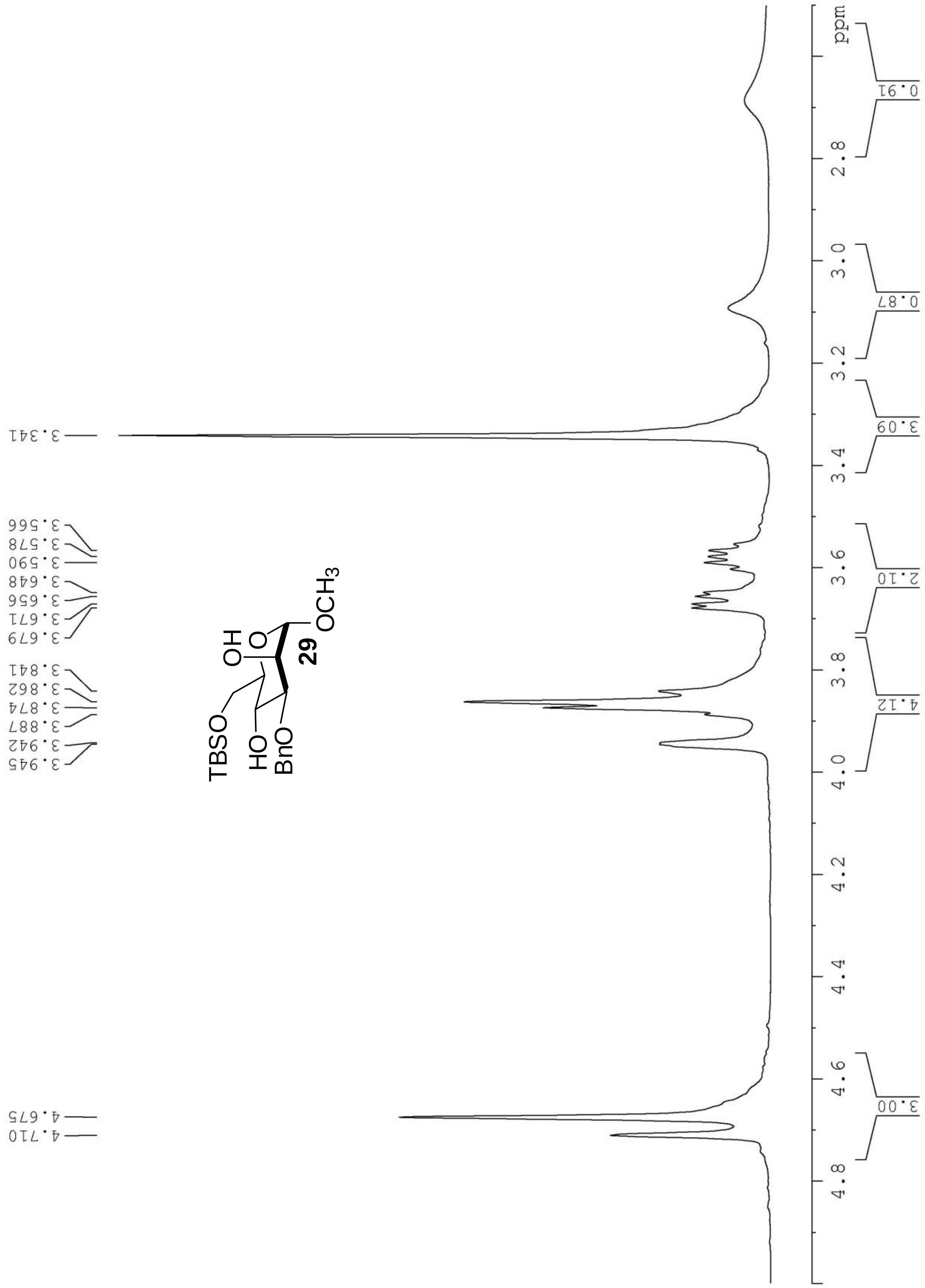
3.341
3.566
3.578
3.590
3.648
3.656
3.671
3.679
3.841
3.862
3.874
3.887
3.942
3.945
4.675
4.710
TBSO
HO
BnO
OH
O
OCH3
29
ppm
2.8
3.0
3.2
3.4
3.6
3.8
4.0
4.2
4.4
4.6
4.8
0.91
0.87
3.09
2.10
4.12
3.00

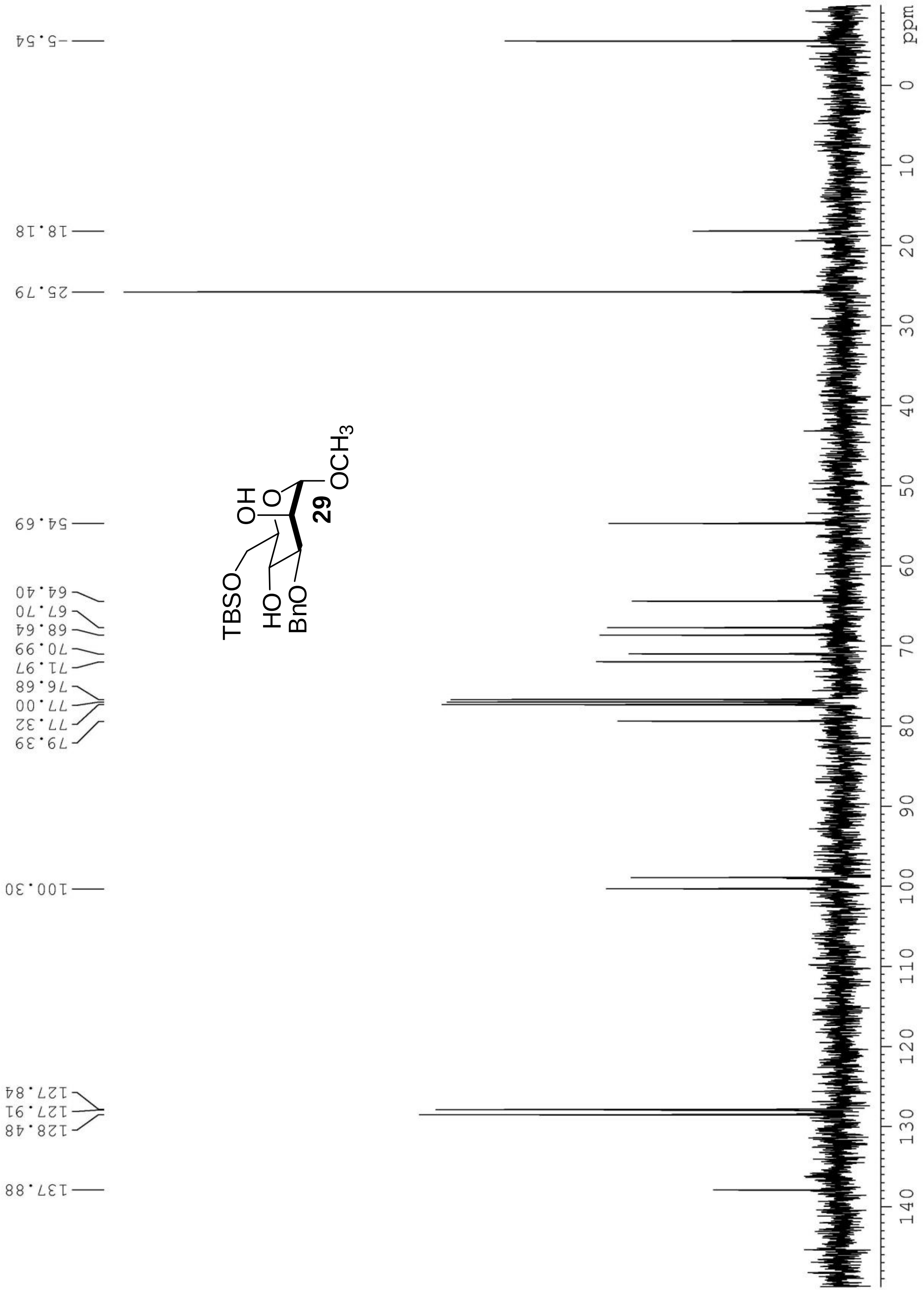
-5.54
18.18
25.79
54.69
64.40
67.70
68.64
70.99
71.97
76.68
77.00
77.32
79.39
100.30
127.84
127.91
128.48
137.88
ppm
TBSO
HO
BnO
OH
O
OCH3
29

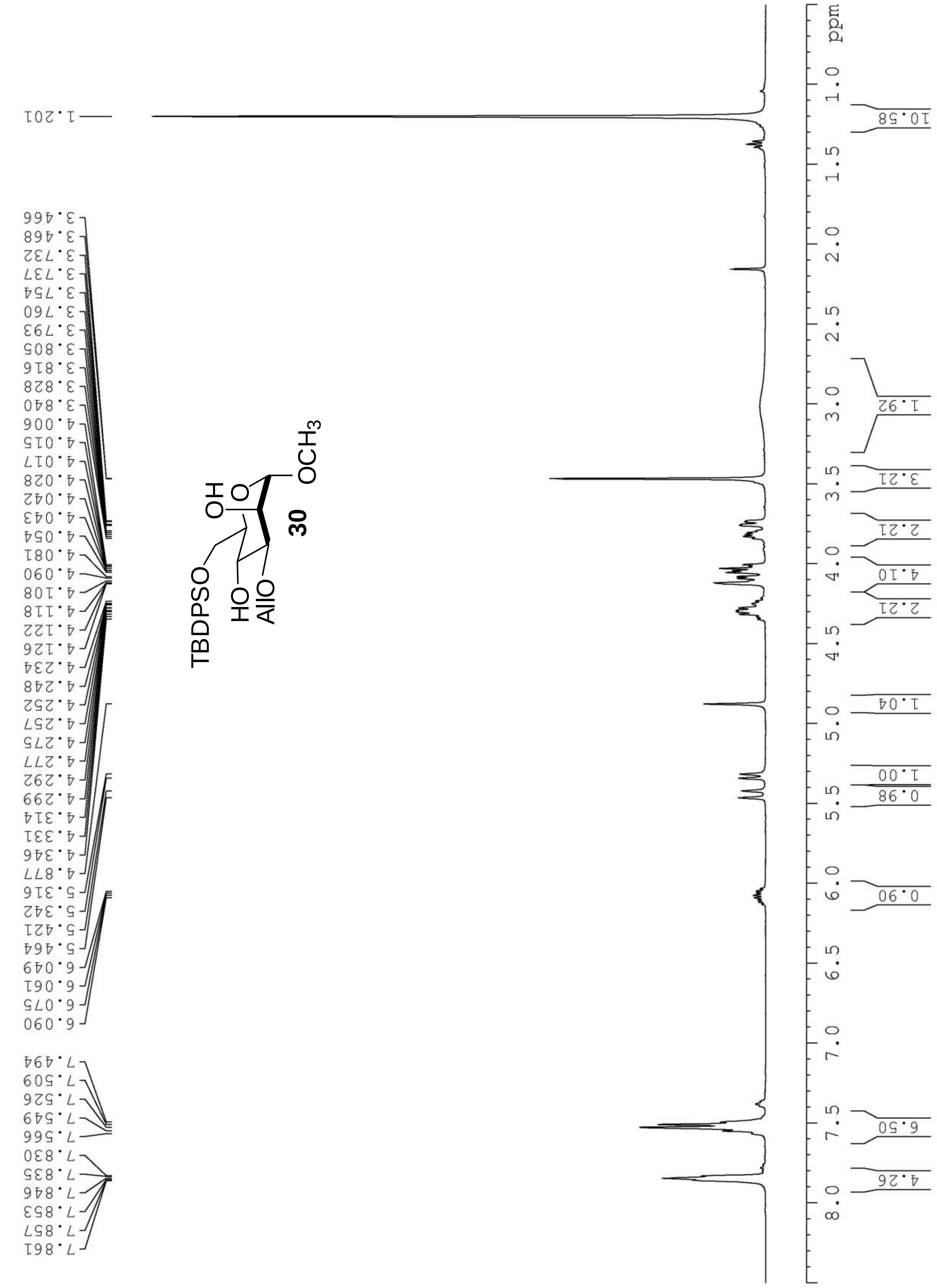
1.201
3.466
3.468
3.732
3.737
3.754
3.760
3.793
3.805
3.816
3.828
3.840
4.006
4.015
4.017
4.028
4.042
4.043
4.054
4.081
4.090
4.108
4.118
4.122
4.126
4.234
4.248
4.252
4.257
4.275
4.277
4.292
4.299
4.314
4.331
4.346
4.877
5.316
5.342
5.421
5.464
6.049
6.061
6.075
6.090
7.494
7.509
7.526
7.549
7.566
7.830
7.835
7.846
7.853
7.857
7.861
TBDPSO
HO
AllO
OH
O
OCH3
30
10.58
1.92
3.21
2.21
4.10
2.21
1.04
1.00
0.98
0.90
6.50
4.26
ppm

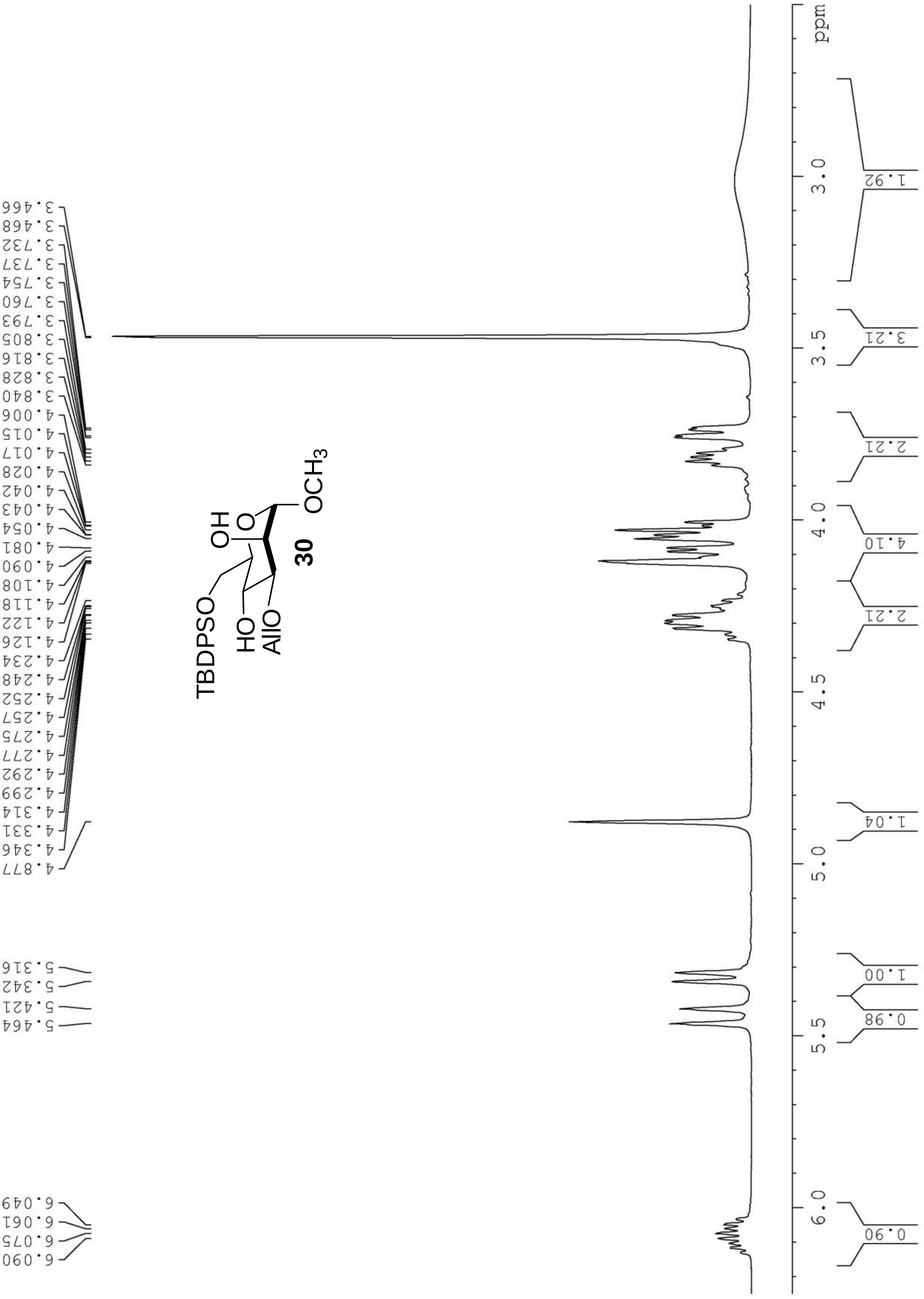
3.466
3.468
3.732
3.737
3.754
3.760
3.793
3.805
3.816
3.828
3.840
4.006
4.015
4.017
4.028
4.042
4.043
4.054
4.081
4.090
4.108
4.118
4.122
4.126
4.234
4.248
4.252
4.257
4.275
4.277
4.292
4.299
4.314
4.331
4.346
4.877
5.316
5.342
5.421
5.464
6.049
6.061
6.075
6.090
TBDPSO
HO
AllO
OH
O
OCH_3
30
1.92
3.21
2.21
4.10
2.21
1.04
1.00
0.98
0.90
3.0
3.5
4.0
4.5
5.0
5.5
6.0
ppm

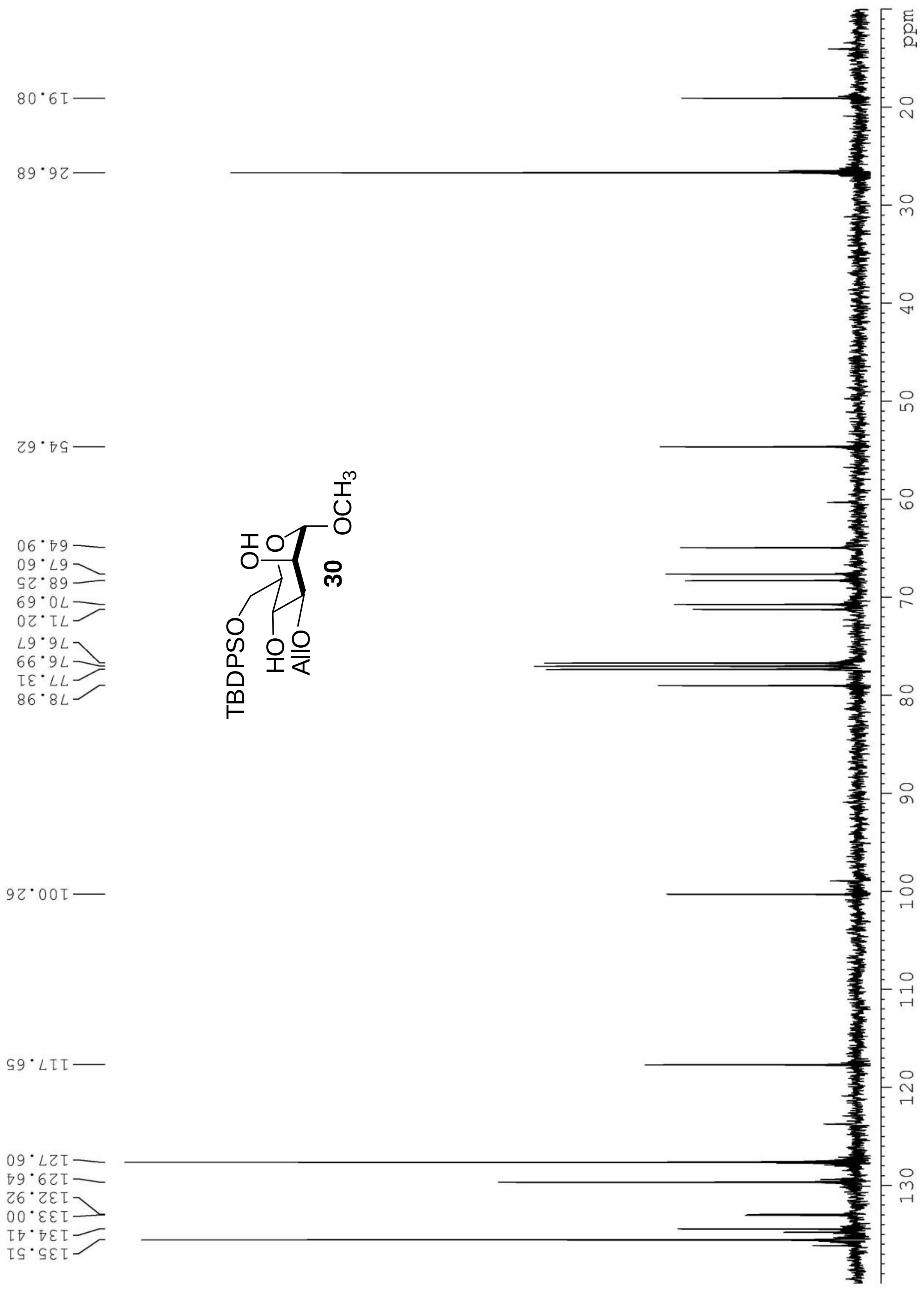
19.08
26.68
54.62
64.90
67.60
68.25
70.69
71.20
76.67
76.99
77.31
78.98
100.26
117.65
127.60
129.64
132.92
133.00
134.41
135.51
TBDPSO
HO
AllO
OH
O
OCH_3
30
ppm

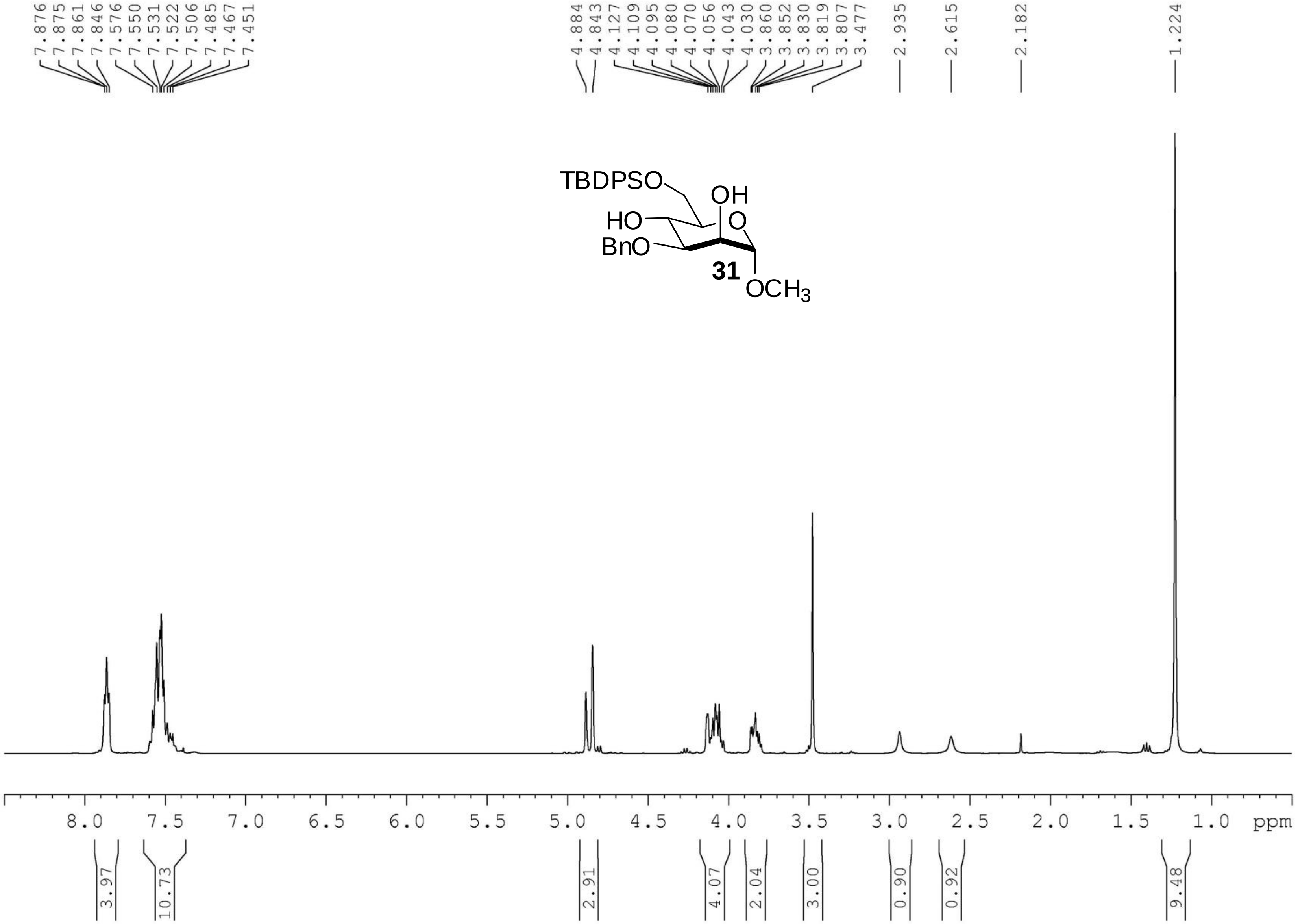
7.876
7.875
7.861
7.846
7.576
7.550
7.531
7.522
7.506
7.485
7.467
7.451
4.884
4.843
4.127
4.109
4.095
4.080
4.070
4.056
4.043
4.030
3.860
3.852
3.830
3.819
3.807
3.477
2.935
2.615
2.182
1.224
TBDPSO
OH
HO
O
BnO
31
OCH_3
8.0
7.5
7.0
6.5
6.0
5.5
5.0
4.5
4.0
3.5
3.0
2.5
2.0
1.5
1.0
ppm
3.97
10.73
2.91
4.07
2.04
3.00
0.90
0.92
9.48

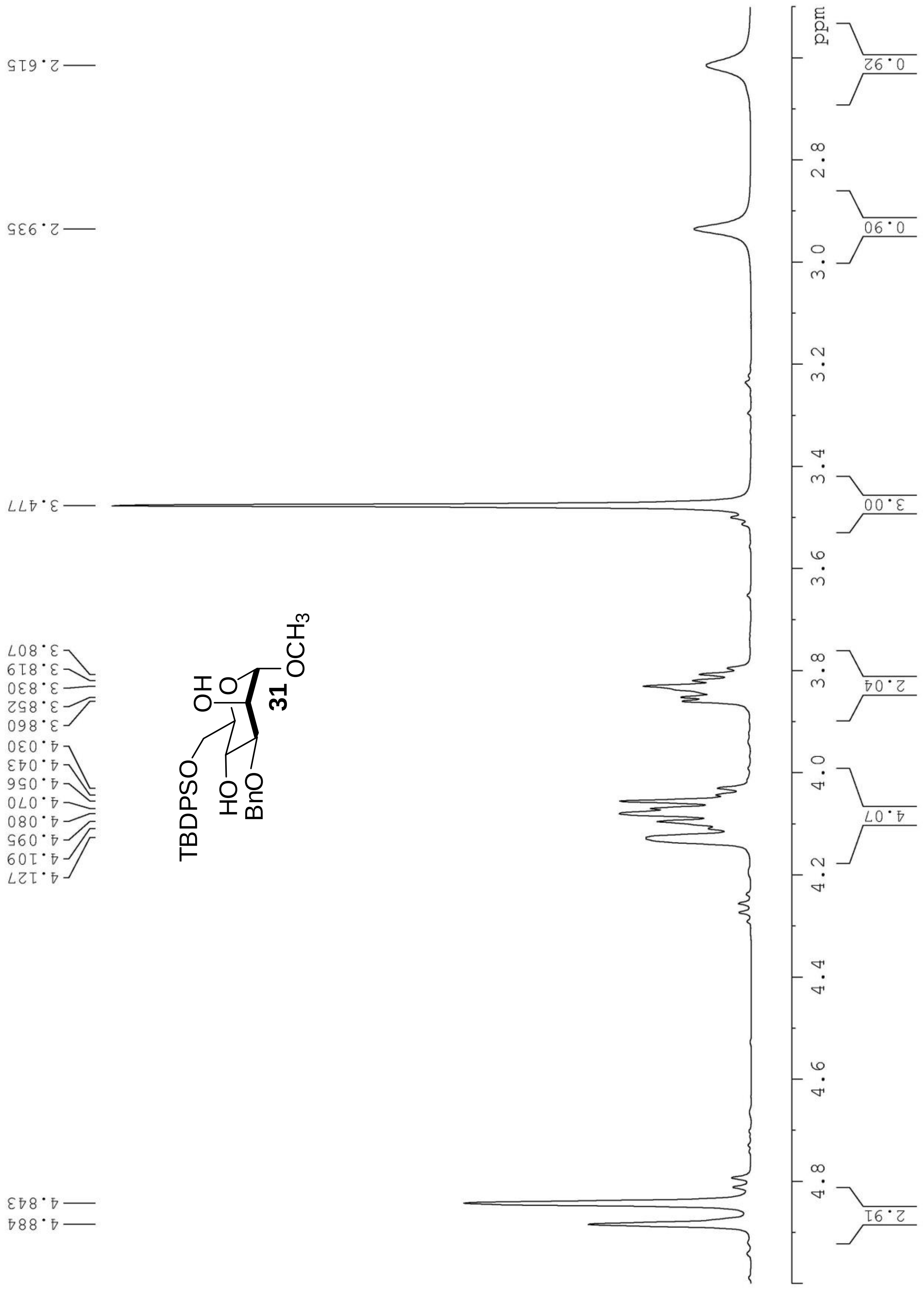
2.615
2.935
3.477
3.807
3.819
3.830
3.852
3.860
4.030
4.043
4.056
4.070
4.080
4.095
4.109
4.127
4.843
4.884
TBDPSO
HO
BnO
OH
O
OCH3
31
ppm
2.8
3.0
3.2
3.4
3.6
3.8
4.0
4.2
4.4
4.6
4.8
0.92
0.90
3.00
2.04
4.07
2.91

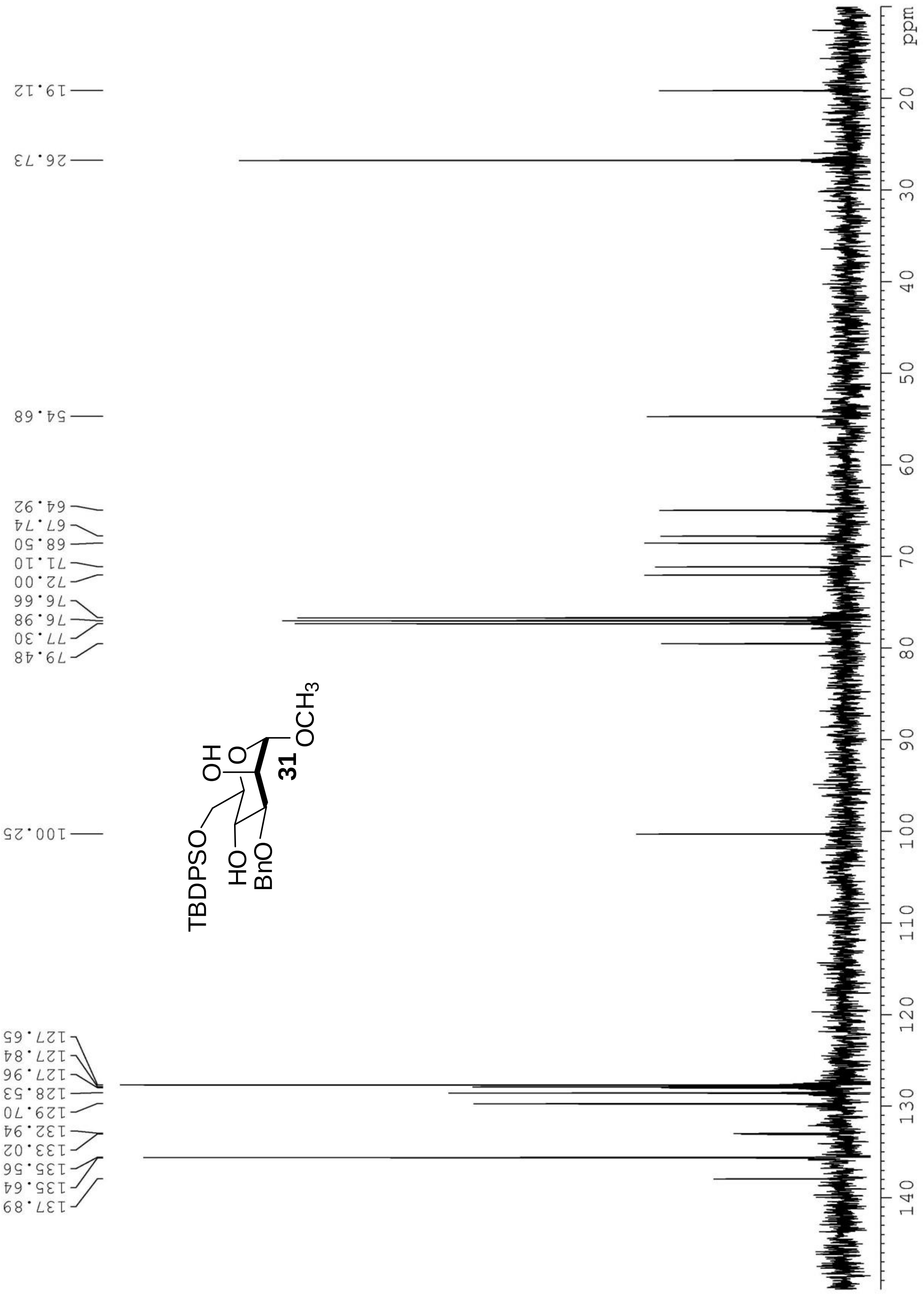

19.12
26.73
54.68
64.92
67.74
68.50
71.10
72.00
76.66
76.98
77.30
79.48
100.25
127.65
127.84
127.96
128.53
129.70
132.94
133.02
135.56
135.64
137.89
TBDPSO
HO
BnO
OH
O
OCH3
31
ppm

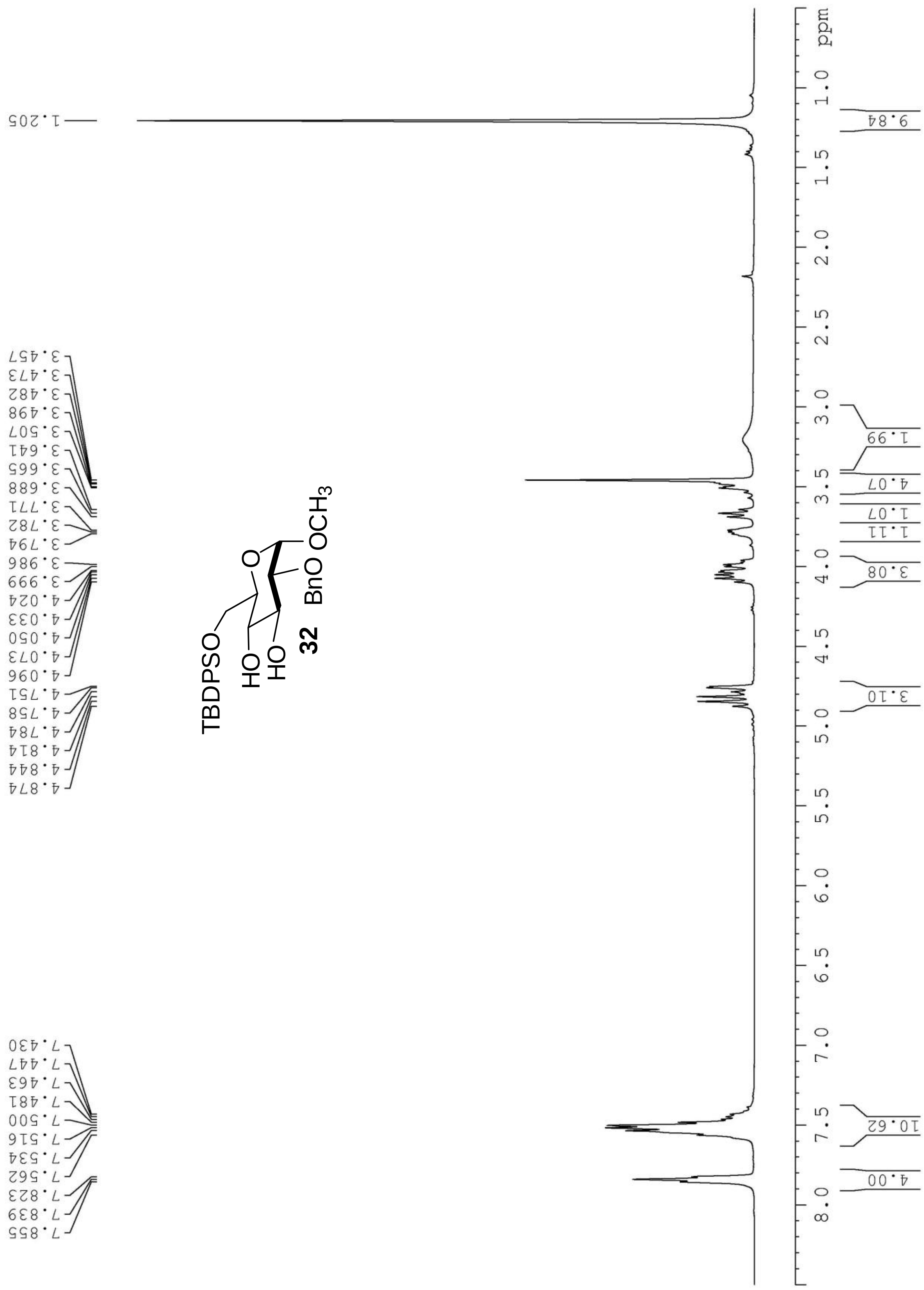
1.205
3.457
3.473
3.482
3.498
3.507
3.641
3.665
3.688
3.771
3.782
3.794
3.986
3.999
4.024
4.033
4.050
4.073
4.096
4.751
4.758
4.784
4.814
4.844
4.874
7.430
7.447
7.463
7.481
7.500
7.516
7.534
7.562
7.823
7.839
7.855
TBDPSO
HO
HO
BnO
OCH3
32
9.84
1.99
4.07
1.07
1.11
3.08
3.10
10.62
4.00
ppm

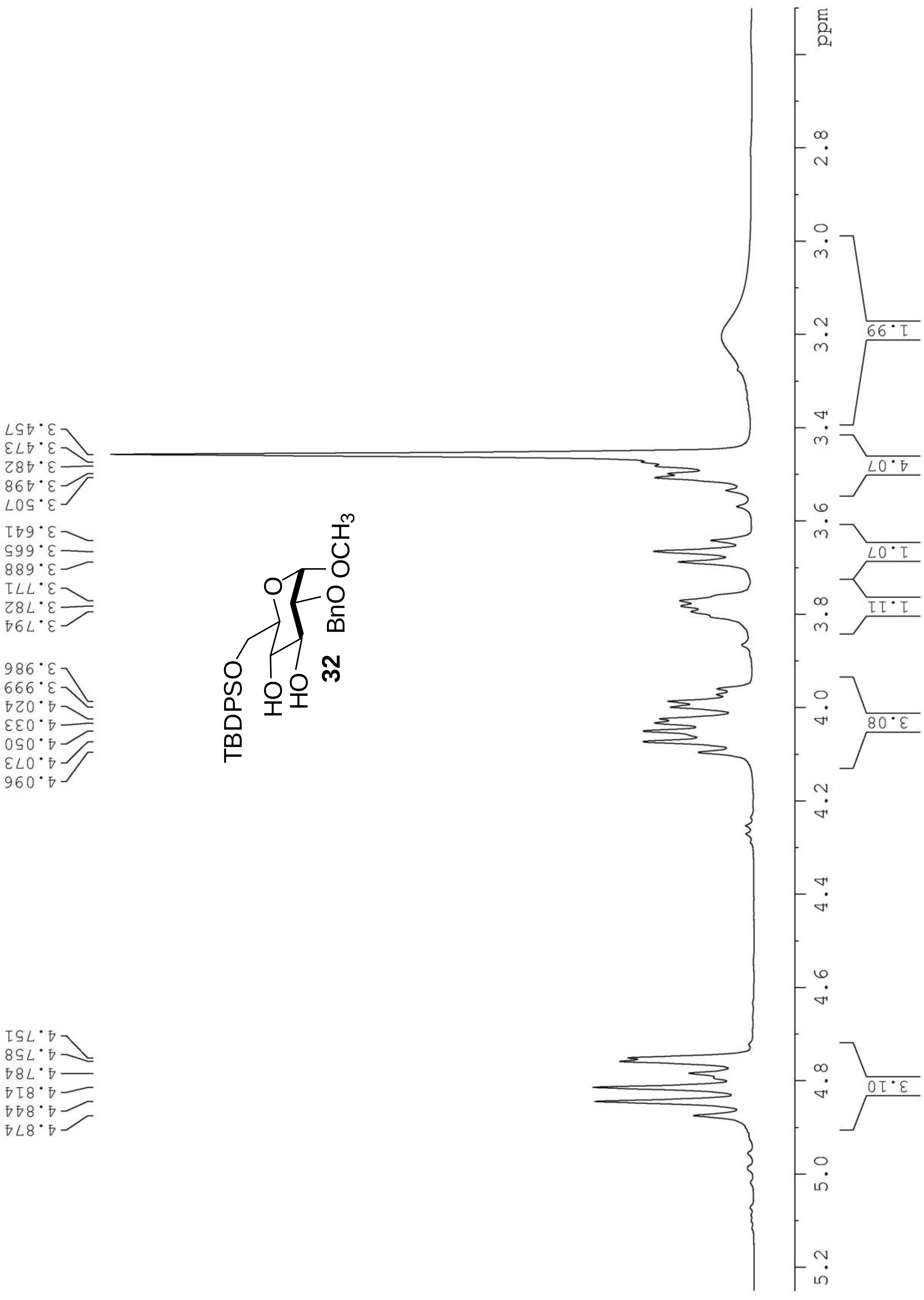

3.457
3.473
3.482
3.498
3.507
3.641
3.665
3.688
3.771
3.782
3.794
3.986
3.999
4.024
4.033
4.050
4.073
4.096
4.751
4.758
4.784
4.814
4.844
4.874
TBDPSO
HO
HO
O
BnO
OCH3
32
1.99
4.07
1.07
1.11
3.08
3.10
ppm
2.8
3.0
3.2
3.4
3.6
3.8
4.0
4.2
4.4
4.6
4.8
5.0
5.2

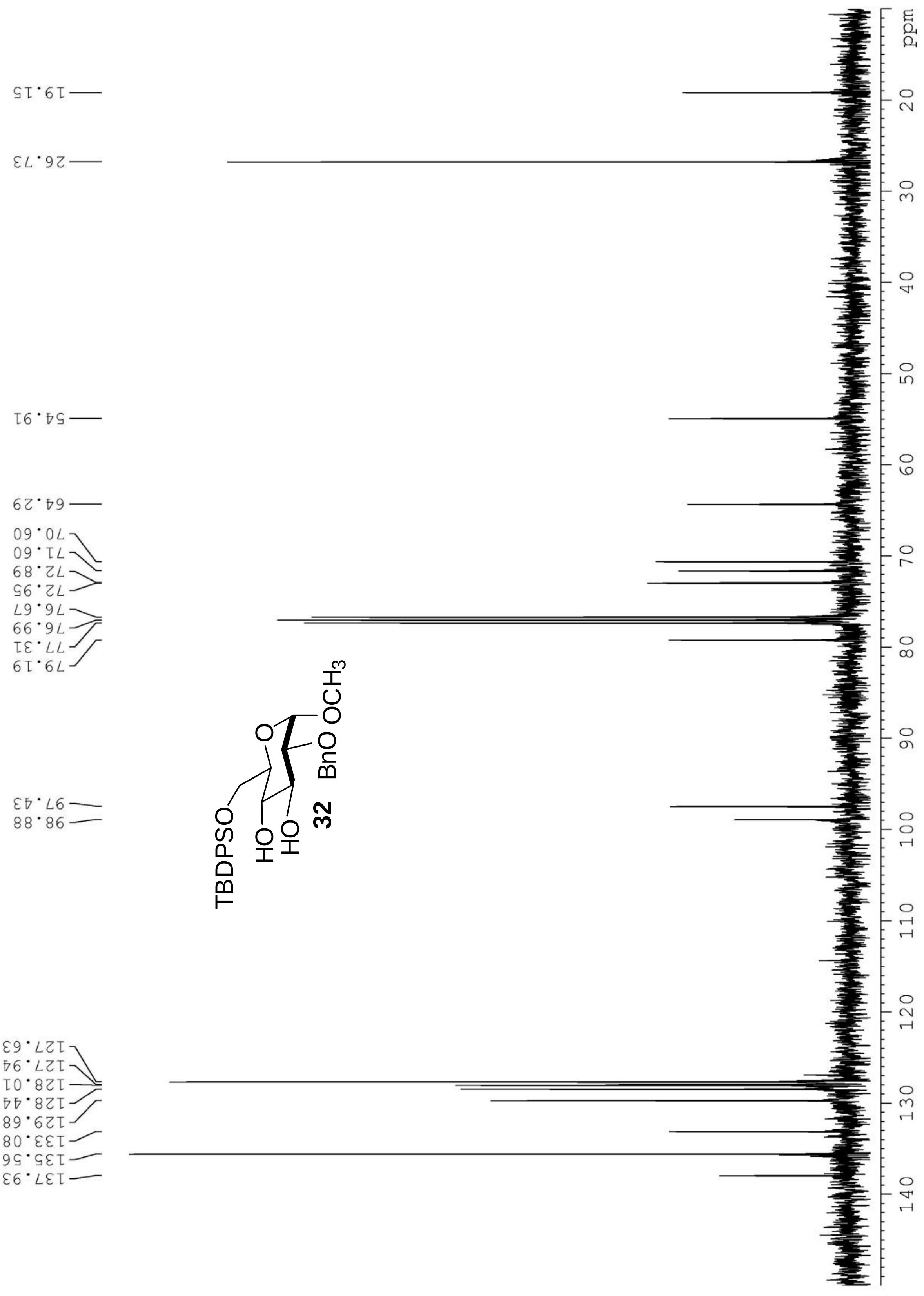

19.15
26.73
54.91
64.29
70.60
71.60
72.89
72.95
76.67
76.99
77.31
79.19
97.43
98.88
127.63
127.94
128.01
128.44
129.68
133.08
135.56
137.93
TBDPSO
HO
HO
BnO
OCH_3
O
32
ppm
20
30
40
50
60
70
80
90
100
110
120
130
140

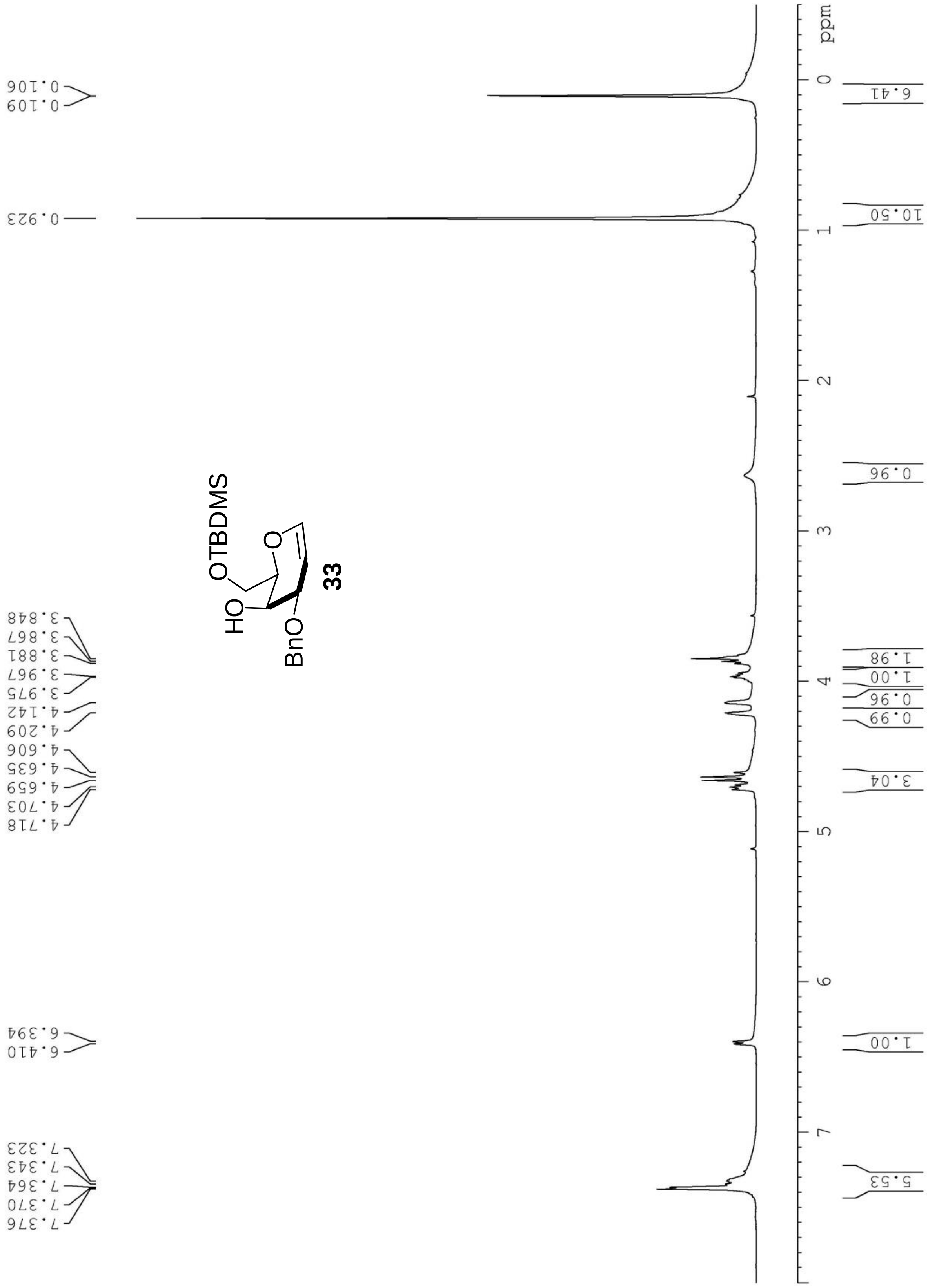
OTBDMS
O
HO
BnO
33
0.106
0.109
0.923
3.848
3.867
3.881
3.967
3.975
4.142
4.209
4.606
4.635
4.659
4.703
4.718
6.394
6.410
7.323
7.343
7.364
7.370
7.376
6.41
10.50
0.96
1.98
1.00
0.96
0.99
3.04
1.00
5.53
ppm
0
1
2
3
4
5
6
7

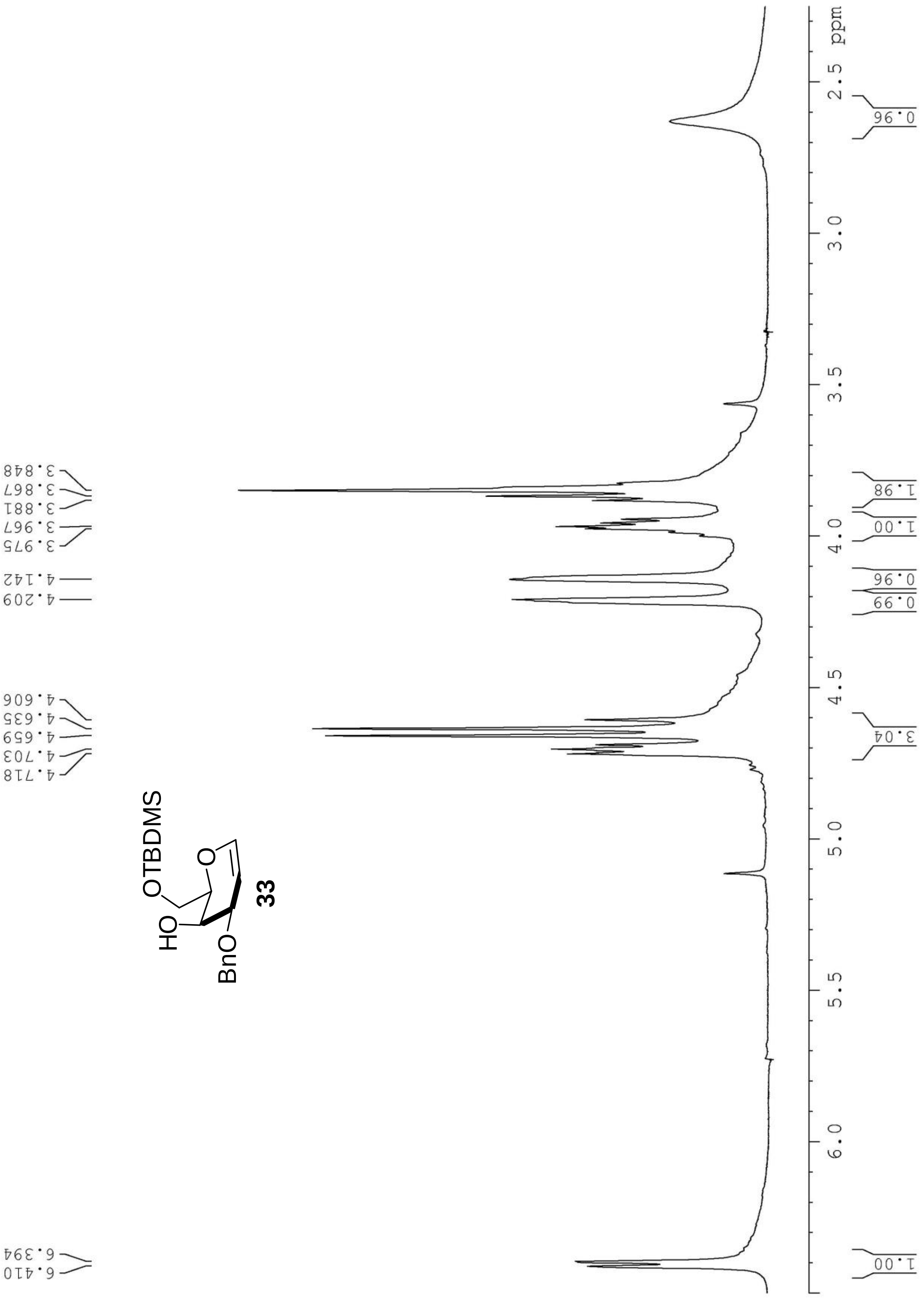

3.848
3.867
3.881
3.967
3.975
4.142
4.209
4.606
4.635
4.659
4.703
4.718
6.394
6.410
OTBDMS
HO
O
BnO
33
0.96
1.98
1.00
0.96
0.99
3.04
1.00
2.5
3.0
3.5
4.0
4.5
5.0
5.5
6.0
ppm

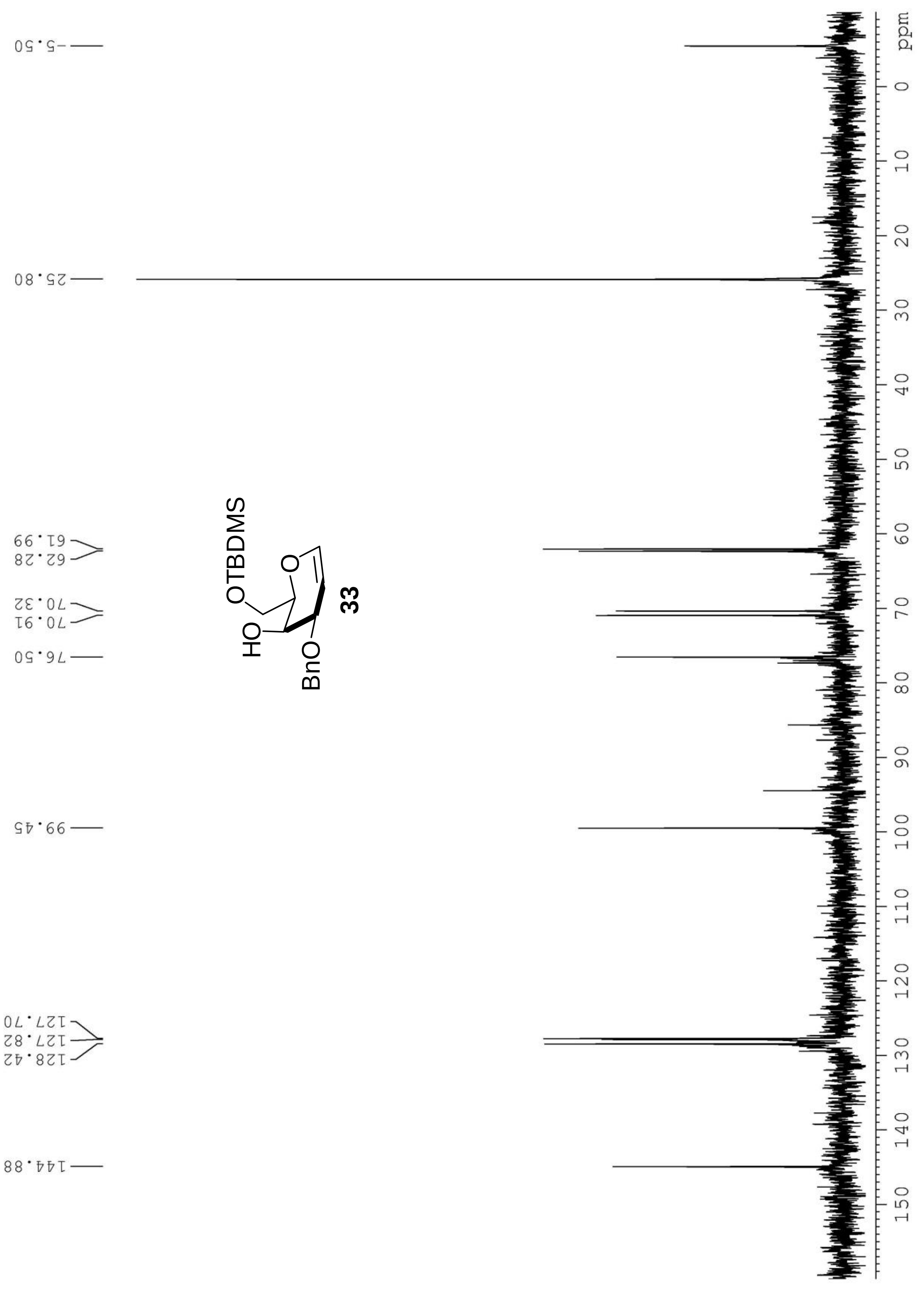
-5.50
25.80
61.99
62.28
70.32
70.91
76.50
99.45
127.70
127.82
128.42
144.88
OTBDMS
O
HO
BnO
33
ppm
0
10
20
30
40
50
60
70
80
90
100
110
120
130
140
150

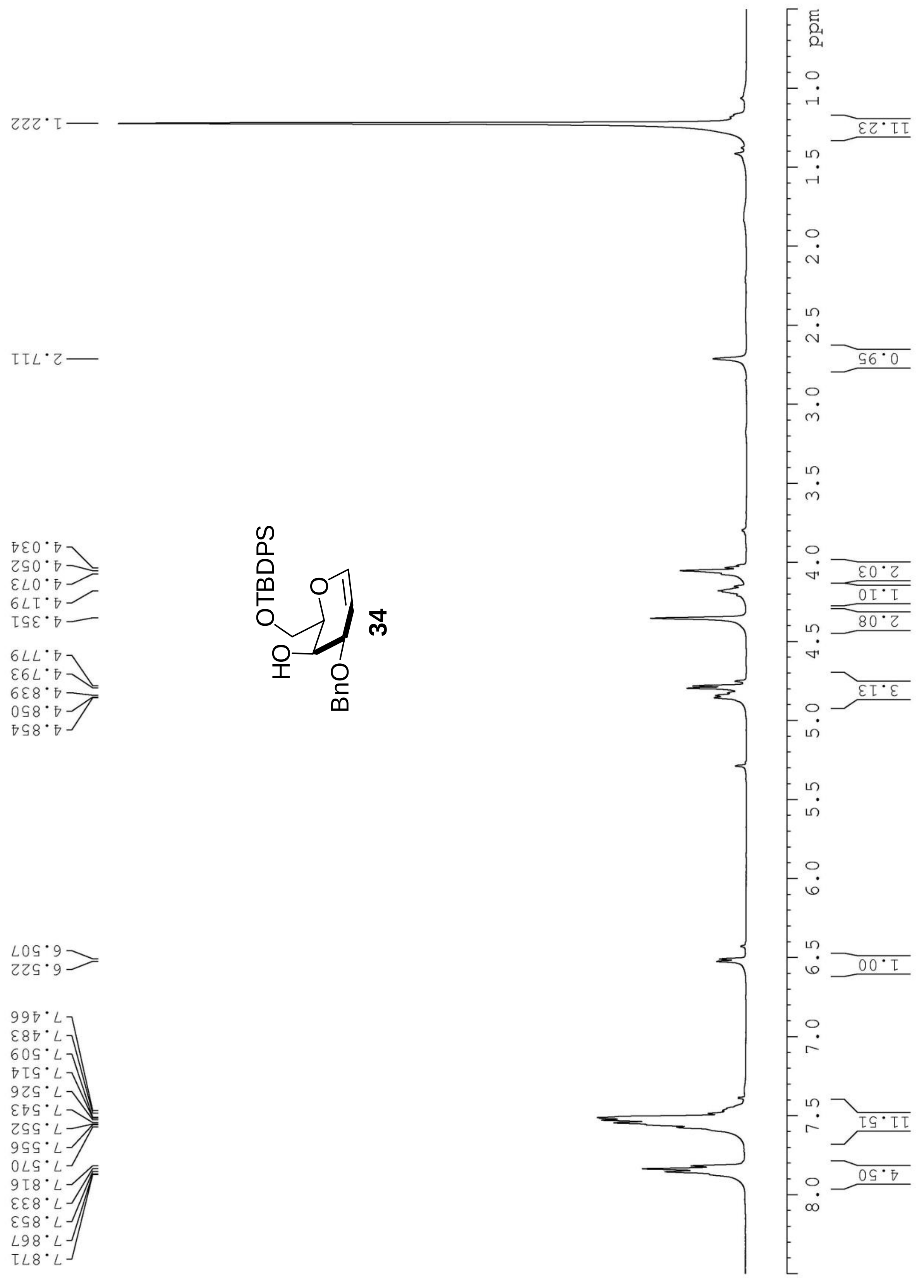

OTBDPS
O
HO
BnO
34
1.222
2.711
4.034
4.052
4.073
4.179
4.351
4.779
4.793
4.839
4.850
4.854
6.507
6.522
7.466
7.483
7.509
7.514
7.526
7.543
7.552
7.556
7.570
7.816
7.833
7.853
7.867
7.871
11.23
0.95
2.03
1.10
2.08
3.13
1.00
11.51
4.50
ppm

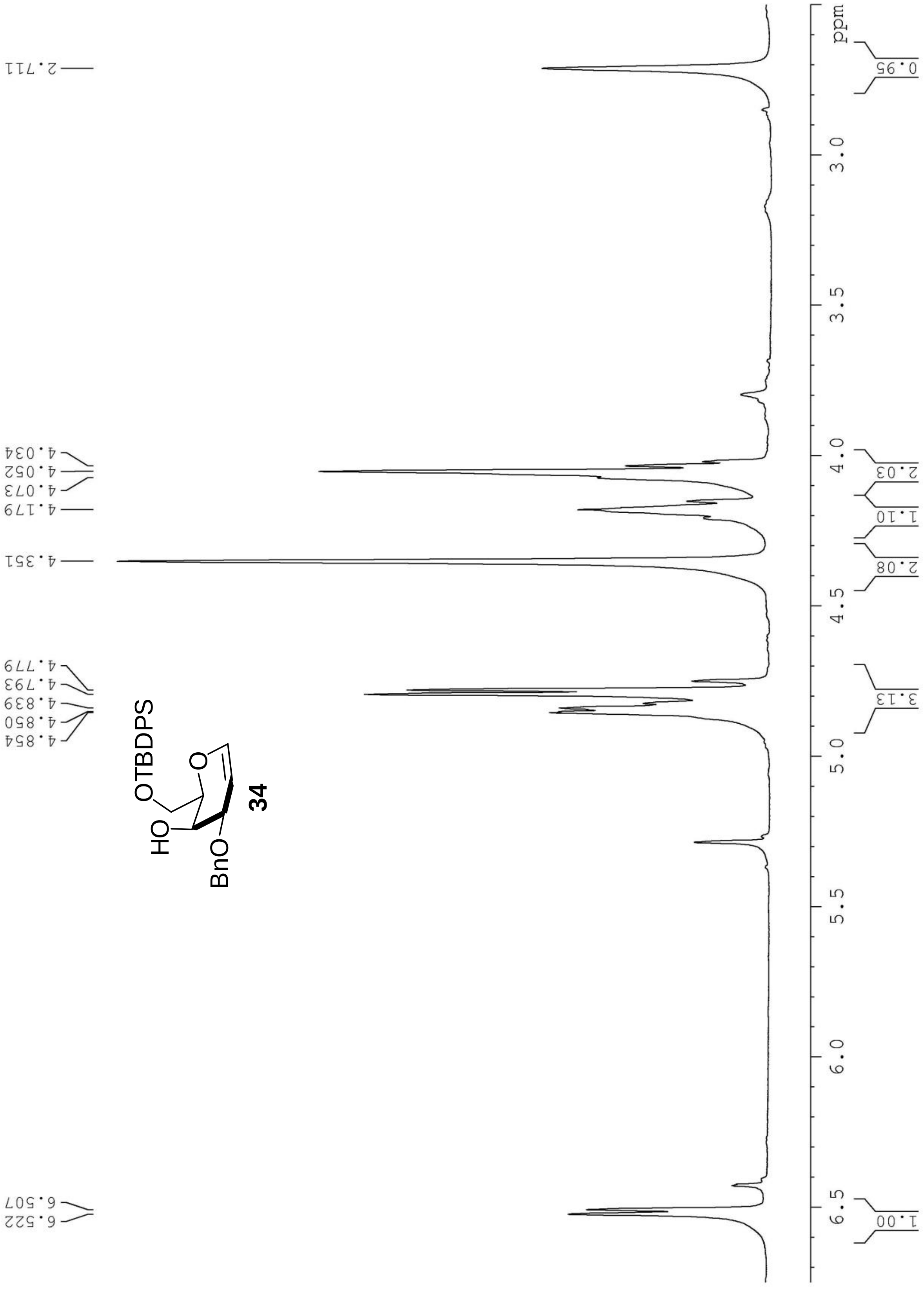

2.711
4.034
4.052
4.073
4.179
4.351
4.779
4.793
4.839
4.850
4.854
6.507
6.522
OTBDPS
HO
O
BnO
34
ppm
3.0
3.5
4.0
4.5
5.0
5.5
6.0
6.5
0.95
2.03
1.10
2.08
3.13
1.00

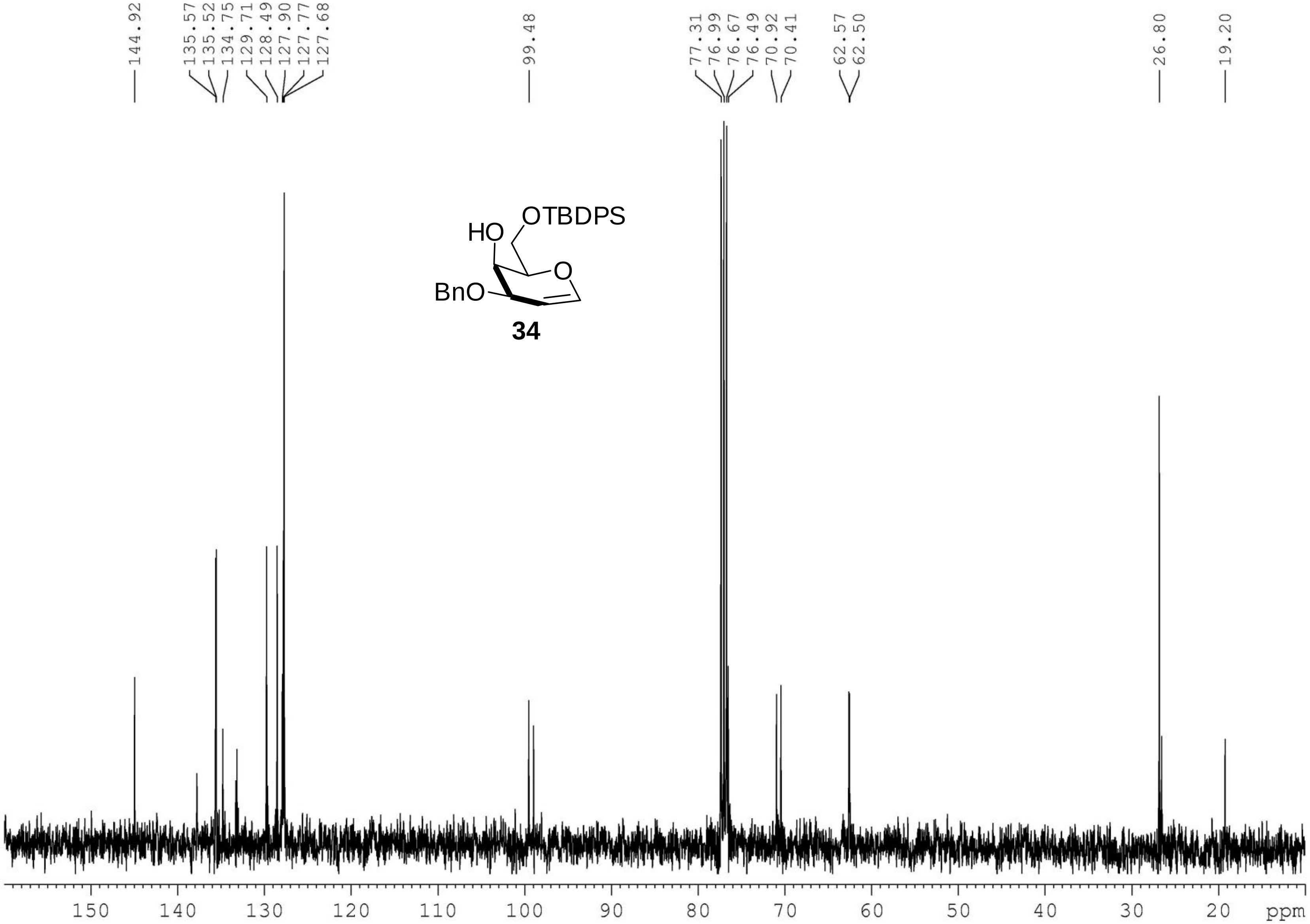
144.92
135.57
135.52
134.75
129.71
128.49
127.90
127.77
127.68
99.48
77.31
76.99
76.67
76.49
70.92
70.41
62.57
62.50
26.80
19.20
OTBDPS
HO
O
BnO
34
150
140
130
120
110
100
90
80
70
60
50
40
30
20
ppm

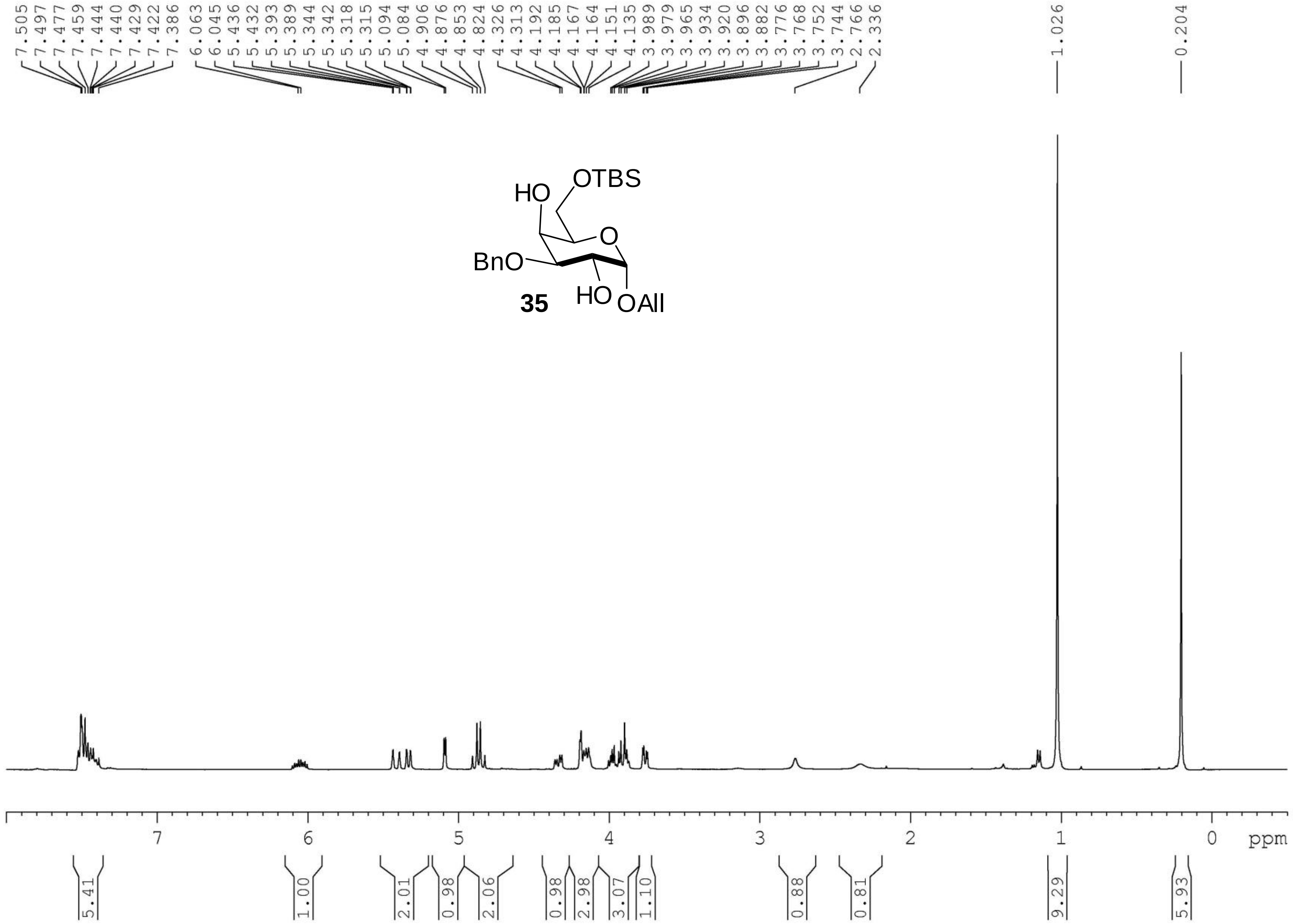
7.505
7.497
7.477
7.459
7.444
7.440
7.429
7.422
7.386
6.063
6.045
5.436
5.432
5.393
5.389
5.344
5.342
5.318
5.315
5.094
5.084
4.906
4.876
4.853
4.824
4.326
4.313
4.192
4.185
4.167
4.164
4.151
4.135
3.989
3.979
3.965
3.934
3.920
3.896
3.882
3.776
3.768
3.752
3.744
2.766
2.336
1.026
0.204
HO
OTBS
O
BnO
35
HO
OAll
7
6
5
4
3
2
1
0
ppm
5.41
1.00
2.01
0.98
2.06
0.98
2.98
3.07
1.10
0.88
0.81
9.29
5.93

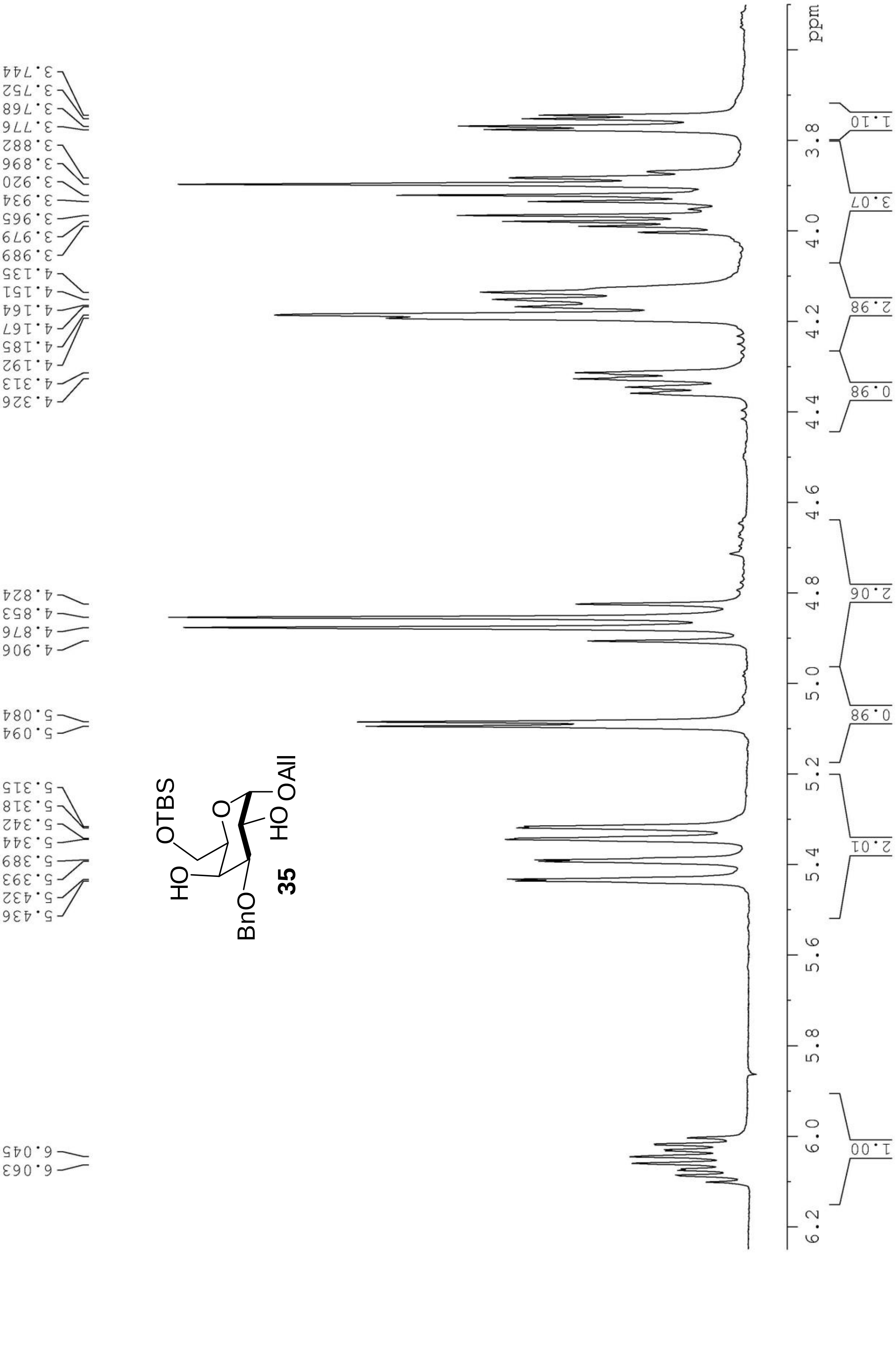
OTBS
HO
O
BnO
HO
OAll
35
3.744
3.752
3.768
3.776
3.882
3.896
3.920
3.934
3.965
3.979
3.989
4.135
4.151
4.164
4.167
4.185
4.192
4.313
4.326
4.824
4.853
4.876
4.906
5.084
5.094
5.315
5.318
5.342
5.344
5.389
5.393
5.432
5.436
6.045
6.063
1.10
3.07
2.98
0.98
2.06
0.98
2.01
1.00
ppm

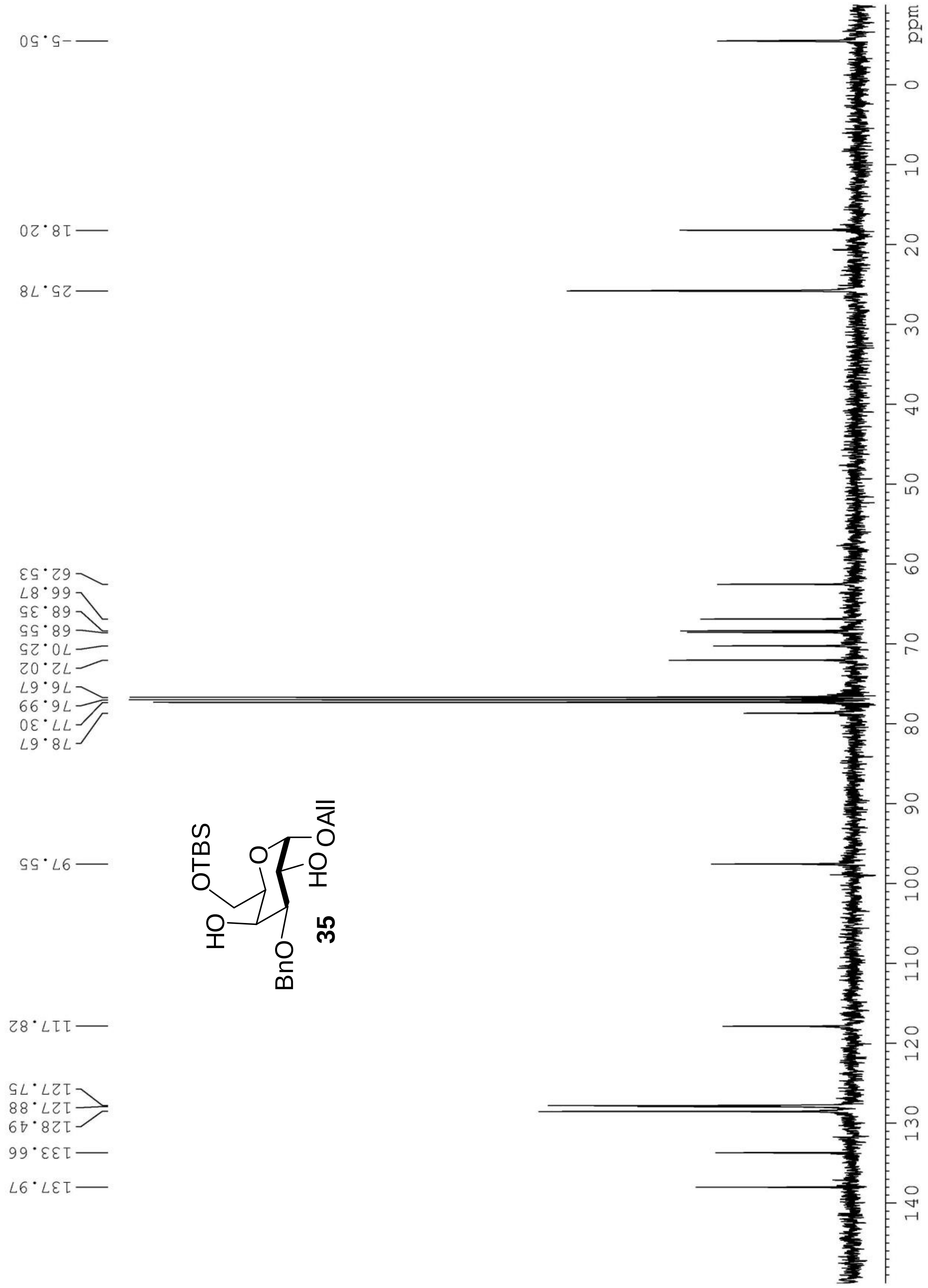
-5.50
18.20
25.78
62.53
66.87
68.35
68.55
70.25
72.02
76.67
76.99
77.30
78.67
97.55
117.82
127.75
127.88
128.49
133.66
137.97
OTBS
HO
O
OAll
HO
BnO
35
ppm
